# Physiological Basis and Transcriptional Profiling of Three Salt-Tolerant Mutant Lines of Rice

**DOI:** 10.3389/fpls.2016.01462

**Published:** 2016-09-28

**Authors:** Concha Domingo, Eric Lalanne, María M. Catalá, Eva Pla, Juan L. Reig-Valiente, Manuel Talón

**Affiliations:** ^1^Genomics Department, Instituto Valenciano de Investigaciones AgrariasValencia, Spain; ^2^Oryzon Genomics Diagnóstico SLCornellà de Llobregat–Barcelona, Spain; ^3^Ebre Field Station, Institut de Recerca i Tecnologia AgroalimentariesAmposta, Spain

**Keywords:** salt tolerance, rice, mutant, microarray hybridization, abiotic stress

## Abstract

Salinity is a complex trait that affects growth and productivity in many crops, including rice. Mutation induction, a useful tool to generate salt tolerant plants, enables the analysis of plants with similar genetic background, facilitating the understanding of the salt tolerance mechanisms. In this work, we generated three salt tolerant mutant lines by irradiation of a salt-sensitive cultivar plants and screened M2 plants at seedling stage in the presence of high salinity. These three lines, *SaT20, SaS62*, and *SaT58*, showed different responses to salinity, but exhibited similar phenotype to wild type plants, except *SaT20* that displayed shorter height when grown in the absence of salt. Under salt conditions, all three mutants and the parental line showed similar reduction in yield, although relevant differences in other physiological parameters, such as Na^+^ accumulation in healthy leaves of *SaT20*, were registered. Microarray analyses of gene expression profiles in roots revealed the occurrence of common and specific responses in the mutants. The three mutants showed up-regulation of responsive genes, the activation of oxido-reduction process and the inhibition of ion transport. The participation of jasmonate in the plant response to salt was evident by down-regulation of a gene coding for a jasmonate O-methyltransferase. Genes dealing with lipid transport and metabolism were, in general, up-regulated except in *SaS62*, that also exhibited down-regulation of genes involved in ion transport and Ca^2+^ signal transduction. The two most tolerant varieties, *SaS62* and *SaT20*, displayed lower levels of transcripts involved in K^+^ uptake. The physiological study and the description of the expression analysis evidenced that the three lines showed different responses to salt: *SaT20* showed a high Na^+^ content in leaves, *SaS62* presented an inhibition of lipid metabolism and ion transport and *SaT58* differs in both features in the response to salinity. The analysis of these salt tolerant mutants illustrates the complexity of this trait evidencing the breadth of the plant responses to salinity including simultaneous cooperation of alternative or complementary mechanisms.

## Introduction

Salinity is an increasing problem that affects an important portion of land worldwide, since it is estimated that ~20% of the irrigated areas are affected by soil salinization (Yeo, 1998). Rice is considered as a salt-sensitive plant. Salinity affects rice seedling growth, produces leaf damage, delays panicle emergence, and reduces yield, as evidenced by a reduction in the length of panicle, the number of spikelets per panicle and, consequently, the final number of grains (Zeng and Shannon, 2000). Tolerance of rice plants to salt stress varies according to the stage of development: Rice is relatively tolerant during germination, tillering and maturing, but sensitive during a short period after germination and at panicle initiation (Zeng and Shannon, 2000). This observation may suggest that the mechanisms of response during these salt several sensitivity stages may be different and that tolerant plants presumably may combine these different kinds of responses (Moradi et al., 2003). Tolerance to salinity is a complex trait due to the multiple effects caused by the presence of high concentration of salt. Most of the salinity effects are due to ion toxicity caused by Cl^−^ and overall by Na^+^ ions that is the main cause of the physiological damage induced by salinity, including the inhibition of a variety of processes such as K^+^ absorption (Tester and Davenport, 2003). Additionally, the presence of Na^+^ induces a rapid osmotic stress that leads to water deficit. Therefore, the different effects observed during salinization, from reduced turgidity and growth to the loss of the cellular structure by disruption of membranes and inhibition of enzyme activity, are the combined result of drought stress, ionic toxicity, and nutritional imbalance. The physiological bases of salt tolerance during vegetative growth are well established (Hasegawa et al., 2000; Ismail et al., 2007): (1) excluding salt or slowing the transport of ions across the membranes to restore and maintain homeostasis; (2) transport and accumulation of Na^+^ in structural or in older tissues; (3) compartmentalization of toxic ions in vacuoles; (4) rapid stomatal closure; (5) induction of antioxidant systems to cope with the stress; (6) and rapid growth of the plant to dilute the salt concentration in tissues. The multiple physiological alterations caused by salt suggest the existence in plants of different responses and mechanisms to cope with all aspects of this adverse condition.

In most of the soils affected by salinity, crop establishment is a major challenge because rice is sensitive to salt stress during seedling growth. Tolerant varieties at seedling stage are needed to enhance and stabilize productivity. Previous physiological and transcriptome studies have investigated the mechanism of response of rice plants to salt at different stages of development, comparing tolerant *vs.* sensitive cultivars with different genetic backgrounds. Several tolerance-associated genes have been identified by such transcriptome studies (Kawasaki et al., 2001; Chao et al., 2005; Cotsaftis et al., 2011). In this study, we induced genetic variation by gamma-rays irradiation in Bahia, a japonica type salt-sensitive cultivar, and screened the resulting M2 progeny in order to obtain salt-tolerant rice plants (Takagi et al., 2015). We identified three mutants with increased tolerance to salt that were further characterized at the physiological, agronomical, and molecular levels. To study the mechanisms of response and the genetic basis of salt tolerance in rice, we compared the root transcript profiles of the tree tolerant mutants and the sensitive parental line and identified the genes that were differentially expressed under high salt conditions.

## Materials and methods

### Plant material and screening of irradiated mutant libraries

To determine the optimum salt concentration for tolerance screening seeds were germinated on a Styrofoam sheet with a nylon net bottom. The sheets were floated on a plastic tray filled with distilled water for 3 days and then with nutrient solution at pH 5.5 for 6 days (12 h light:12 h dark; 24°C and 70% RH). Salt was added to the culture medium to reach final 0, 40, 80, or 120 mM NaCl concentrations. Plants were scored at 6 and 11 days after the onset of salt treatment. The following parameters were determined: Fresh weight, dried weight, and culm length. A total of 20,600 M2 plants, cv. Bahia, from gamma and fast neutron radiation libraries (Domingo et al., 2007), were screened in the presence of 120 mM NaCl for the identification of salt-tolerant lines in two independent experiments. Pre-germinated seeds were sown and grown in the same conditions as described above. After 6 days from sowing, the culture medium was salinized to reach a final concentration 60 mM NaCl and after 4 days, salinity was increased to 120 mM. Seedlings were scored twice, after 1 week and after 2–3 weeks, for increased tolerance, according to the 1–9 scale described in the Standard Evaluation System (SES) for salinity tolerance at seedling stage (International Rice Research Institute, 1996). Individual plants showing increased tolerance were isolated and grown on soil till maturity and seeds were collected. Selected mutant lines were tested in a second analysis in order to confirm the differential behavior in the presence of NaCl. Isolation of homozygous plants from all selected lines was completed at M5 generation.

For ABA sensitivity assay, homozygous mutant and Bahia control seeds were germinated and grown on 1/2 strength MS medium at room temperature (25–28°C) for 3days. Seedlings were transferred to culture boxes containing 1/2 strength MS medium plus 0, 10, or 30μM ABA; each box contained mutant and control Bahia plants. Additionally, another batch of seeds were germinated in the presence of 0, 10, or 30μM ABA and grown under the same conditions. After 7–10days of treatment, the phenotype was recorded. Assays were repeated at least three times.

### Ion content

Plants were grown for 4 weeks in nutrient solution and then for five additional days after adding NaCl to the solution to reach a final concentration of 100 mM NaCl, milder conditions than those used in the screening so that sensitive plants were less affected for analysis. Shoots and roots were washed with deionized water, dried in an oven (70°C) and then ground into fine powder. Na^+^ and K^+^ content were measured by inductively coupled plasma optical emission spectrometry (ICP). Chloride content was estimated according to Gilliam (1971). After saline treatments, leaves and roots were harvested separately and oven-dried for at least 72 h at 70°C. Chloride was extracted from 200 mg dry mass of leaf or root tissue with 0.1 N HNO_3_ in 10% (v/v) glacial acetic acid. Samples were incubated overnight at room temperature and then filtered. Chloride concentration was determined by silver ion titration using a Chloride Analyzer 926 (Corning Ltd. Halstead Essex, UK). At least three independent extractions were performed for each sample.

### Agronomical evaluation

The agronomical evaluation of mutant lines was performed in open field during 2 consecutive years in two different locations with (41.10 dS/m soil saturated extract) and without (4.79 dS/m soil saturated extract) salt in soil (data from soil before flooding) (Figure S5). Trial design consisted of circular blocks of 1 m^2^ randomly distributed, with four replicates, using Bahia as control. Seeds were sown manually by direct seeding in May and harvested in September. Maximum temperatures were due on August, reaching a mean value of 28.8 during the first year and 29.9°C during second year of experimental assays (Table S1). Fields were irrigated by flooding with a water flow of 1.8 ls^−1^ Ha^−1^. Salt concentration in soil was measured before and during the trials. Water conductivity was similar in both locations, ranging from 0.7 to 1.7 dS/m along the growth period.

### Microarray analysis

*SaT20, SaS62, SaT58*, and control Bahia seedlings were grown hydroponically under tightly controlled conditions (12 h light with ~450 μmol quanta m^−2^ s^−1^, 12 h dark; 25 and 21°C during light and dark period, respectively; relative humidity of ~65%). The nutrient solution was based on Yoshida as reported by Yang et al. (1994), with the following modifications: MgSO4 0.18 mM; FeEDTA 1 mM. Plants were germinated and grown in plastic boxes filled with 9 L nutrient solution (protected from light). After a period of 24 days, salt stress was induced by adding fresh nutrient solution containing 100 mM NaCl. The non-stressed control plants received fresh nutrient solution without NaCl. Four days after the start of the treatment, the three upper leaves of the main stem and the whole root system of the plants were harvested (~5 h after onset of the light period) and immediately frozen in liquid nitrogen. Five replicates per line and treatments were taken.

Leaves and root RNA was isolated using the Plant Total RNA Extraction kit (Qiagen). The quality and concentration of the RNA were analyzed using the Agilent 2100 bioanalyzer and NanoDrop ND-1000 (Thermo Scientific). Samples with an RNA integrity number less than six were discarded. Three biological samples and three replicates per biological sample were analyzed. Microarray analysis was performed using a custom rice transcriptomic array (Oryzon Genomics, Barcelona, Spain) containing 3 × 29,450 probes (3 technical replicates per probe) representing ~20,750 genes. Details of microarray construction, probe labeling, and hybridization conditions are presented in Campo et al. (2014). Results were normalized using a self to self-comparison Bahia vs. Bahia and fold change was as calculated as log_2_ of the ratio of relative expression of sample (Smp) vs. Bahia control (Ctr) plants grown in identical conditions. Only genes for which all replicate spots showed differential expression of −0.7 > *log*_2_*(Smp/Ctr)* > 0.7 and *P-value* < 0.01 were considered as differentially expressed.

Bootstrap analysis with Significance Analysis of Microarrays was used to identify differentially expressed genes using a cut-off of 2 (Tusher et al., 2001). Significance Analysis of Microarrays calculates the fold change and significance of differences in expression. Microarray data were validated by quantitative RT-PCR analyses.

### RNA isolation and quantitative real-time PCR

Total RNA was isolated using the RNeasy plant mini kit (QIAGEN), following the manufacturer's instructions. First-strand cDNA was synthesized from 3 μg of total RNA, using the ThermoScript RT-PCR System (Invitrogen, CA), according to the manufacturer's instructions. Synthesized cDNAs were used for real-time quantitative RT-PCR. One-step Real-time PCR assays were performed as previously described (Domingo et al., 2009). The Real-Time PCR procedure involved incubation at 48°C for 30 min, followed by 45 cycles at 95°C for 2 s, 60°C for 8 s, and 72°C for 8 s. The amplification of a fragment of the ACTIN1 gene was used as a standard control. The identities of the amplicons and the specificity of the reaction were verified by melting curve analysis and by sequencing the reaction product. The sequences of the primers, extension times and number of cycles are provided in Table S5.

## Results

### Salt tolerant mutant identification

**Figure 1:**
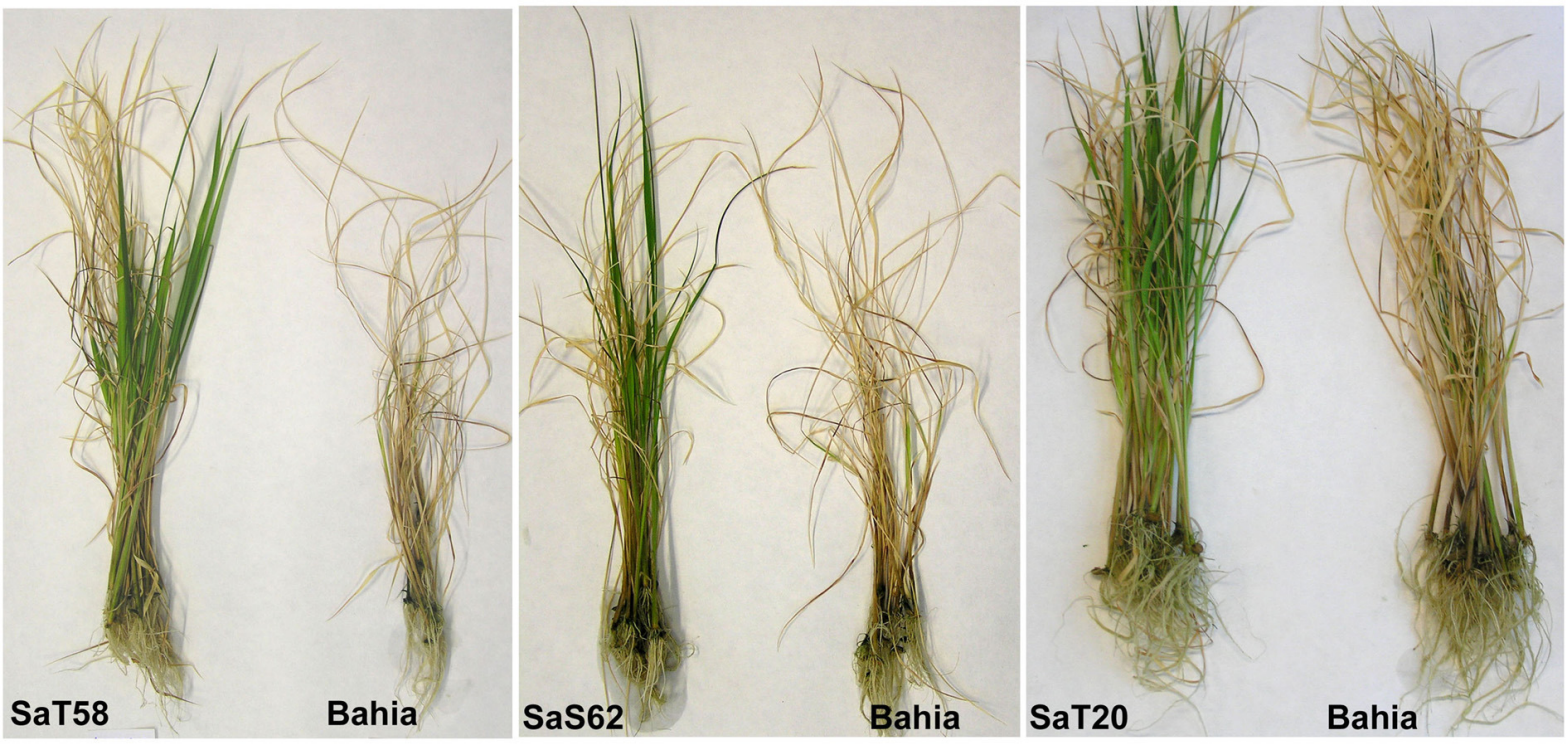
***SaT58*, *SaS62*, and *SaT20* plants grown in hydroponic culture in the presence of 120 mM NaCl during 4 weeks compared to Bahia plants**.

To evaluate the sensitivity of Bahia plants to salinity and to determine the salt tolerance screening conditions, Bahia seedlings were grown in hydroponic culture under different salt conditions by adding NaCl to the culture medium to reach 0, 40, 80, and 120 mM concentration. After 6 and 11 days, fresh weight, dry weight, and culm length were measured. Effects were visible after 6 days of treatments with 80 and 120 mM and plants were clearly affected after 11 days with a decrease in weight and length (Figure S1). Thereafter, 20,600 M2 gamma and fast neutron-induced mutant lines of rice, cv. Bahia were screened at seedling stage in hydroponic culture in the presence of 120 mM NaCl (Figure S2). Plants that survived after 4 weeks of culture were selected and salt tolerance phenotypes were confirmed in the subsequent generation. Sixteen M3 lines showing increased tolerance to the presence of salt were selected and classified at seedling stage according to the SES for salinity tolerance. Three lines with the highest tolerance score were finally selected for further characterization and homozygous plants were obtained by self-pollination (Figure 1, Figure S3). Homozygous *SaT20* and *SaS62* lines, showed high tolerance, while homozygous *SaT58*, showed moderate tolerance to the presence of salt. Plants were grown in pots in the absence of salt in greenhouses under natural daylight condition till maturity. All mutant lines showed a similar phenotype to Bahia plants, except *SaT20* that was 37 cm shorter than Bahia, and displayed similar heading date.

In an attempt to know how ABA sensitivity was affected in these lines, seeds were germinated and grown in the presence or absence of ABA. The results showed that no differences were found in the germination either in the growth parameters of the seedlings compared to wild type Bahia, and therefore ABA pathway regulation does not appeared to be affected in the mutant lines (Figure S4).

### Estimation of total Na^+^ and K^+^ in shoots and roots

Total Na^+^ and K^+^ content analysis in shoots of plants grown in the presence of 100 mM NaCl clearly distinguished *SaT20* plants from the other three genotypes. Salinity stress increased Na^+^ concentration in *SaT20* shoots compared to the other two mutants, reaching a level of 2.6 fold higher than in Bahia plants (Table 1). In contrast, Na^+^ content in *SaS62* shoots was 1.5 fold lower than Bahia. Meanwhile, K^+^ levels in leaves from all mutant lines remained similar or slightly lower than in Bahia plants. As a result, K^+^/Na^+^ ratio was lower in shoots of *SaT20* plants, but on the contrary, higher in *SaS62* plants. When roots were assayed for cation content few differences were found between *SaT20, SaT58*, and Bahia, but *SaS62* roots, as seen in leaves, accumulated lower levels of Na^+^. Cl^−^ content analysis in plants grown under the presence of salt also showed high levels of Cl^−^ in *SaT20* leaves, up to 2.0 fold higher than in Bahia. Furthermore, the other two lines maintained lower levels of Cl^−^ in leaves compared to Bahia. In contrast, all three mutant lines showed moderately higher levels of Cl^−^ in roots.

**Table 1:** **Cation content (as percentage of total cation content of the leaves and roots), and K/Na ratio and Cl^−^ (mg/l) in leaves and roots of *SaT58, SaS62, SaT20*, and Bahia plants grown in the presence of salt for 3 days**.

**Leaves**	**Na (%)**	**K (%)**	**K/Na**
Bahia	2.91 ± 0.10b	4.04 ± 0.04a	1.40 ± 0.04b
SaT58a	2.85 ± 0.07b	4.27 ± 0.09b	1.50 ± 0.01b
SaS62c	1.89 ± 0.04a	4.09 ± 0.00ab	2.17 ± 0.04c
SaT20b	7.63 ± 0.13c	4.22 ± 0.08b	0.55 ± 0.02a
**Roots**	**Na (%)**	**K (%)**	**K/Na**
Bahia	4.12 ± 0.14b	1.21 ± 0.08ab	0.29 ± 0.01b
SaT58a	4.24 ± 0.08b	1.25 ± 0.01b	0.30 ± 0.01b
SaS62c	3.44 ± 0.01a	1.47 ± 0.00c	0.43 ± 0.00c
SaT20b	4.33 ± 0.00b	1.06 ± 0.00a	0.24 ± 0.00a
	**Cl, leaves**		**Cl, roots**
Bahia	62.33 ± 0.67c		46.33 ± 0.33a
SaT58a	50.50 ± 2.63b		50.67 ± 0.67b
SaS62c	41.83 ± 0.31a		51.52 ± 0.58b
SaT20b	125.67 ± 0.92d		51.67 ± 0.88b

Standard error is indicated. Identical letters in each panel show no statistical differences at 0.05 significance level based on Duncan's multiple range test.

### Agronomic characterization

**Figure 2:**
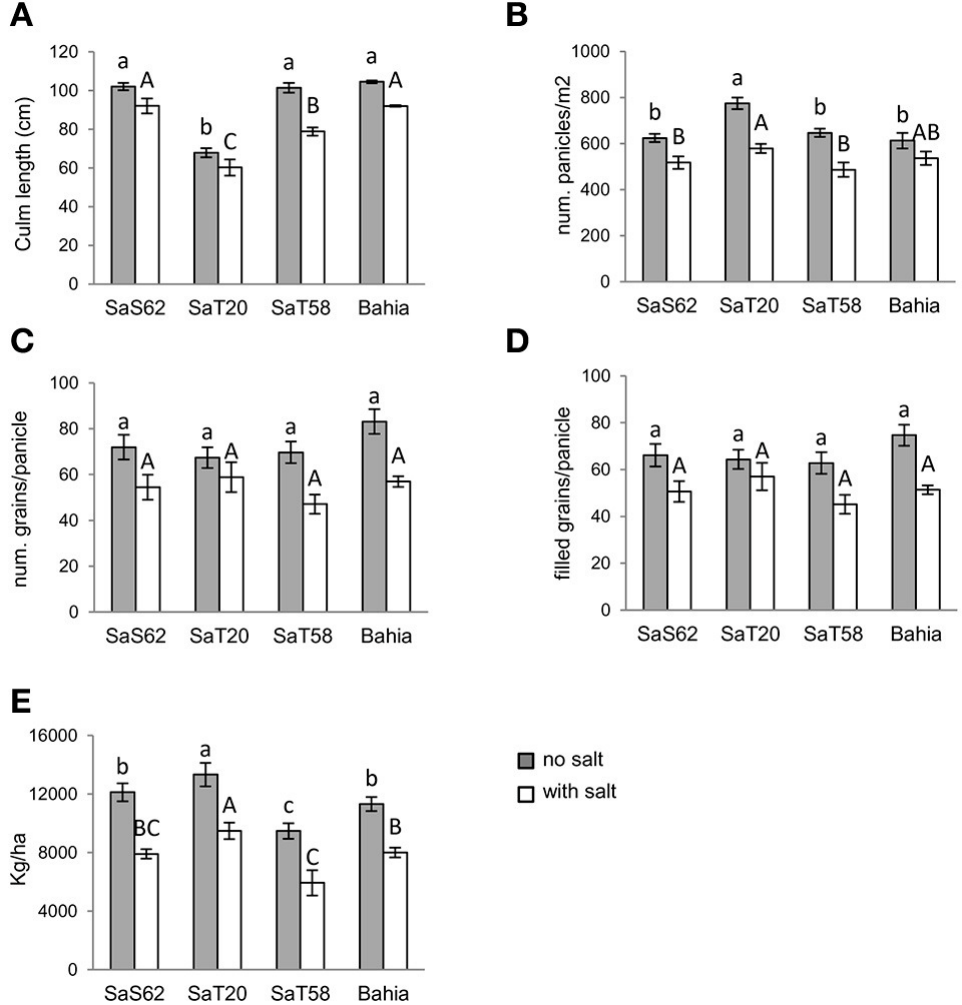
**Mean and standard errors of (A) height, (B) number of panicle per square meter, (C) number of grains per panicle, and (D) number of filled grains per panicle and (E) yield of plants grown in fields under non-salt (solid bars) and salt (open bars) conditions**. The same letter in bars within each genotype indicates no significant difference at 0.05 significance level based on Duncan's multiple range test. Upper case letters refer to non-salt treatment, while lower case letters refer to salt treatment.

Grain yield and other agronomic traits of salt-tolerant lines were analyzed during 2 consecutive years under standard non-saline field condition and natural saline field condition, in a coastal area (Figure S5). Salt-affected soils displayed a high electric conductivity (41.1 dS/m soil saturated extract) that was offset by a low ionic strength of irrigation water (ranging from 0.7 to 1.7 dS/m along the plant growth period). As observed in greenhouse conditions, all mutant lines grown in normal field conditions reached similar height to Bahia, except *SaT20* that was 36.6 cm shorter (Figure 2A). Additionally, *SaT20* produced higher number of panicles than Bahia and the other two lines (Figure 2B). No significant differences could be found among the four genotypes in the total number of grains or number of filled grains per panicle (Figures 2C, D). Furthermore, *SaT20* showed the highest yield when grown under non-saline conditions, displaying 117.8% approximately of wild type Bahia yield. Only *SaT58* line showed lower yield performance than Bahia (Figure 2E).

When grown in saline conditions, all plants showed reduction in plant height, number of panicles, and number of grains per panicle compared to plants grown in soil with low salt concentration (Figure 2). In the presence of high levels of salt, *SaS62, SaT20*, and Bahia, were shorter with a similar reduction in height compared to plants grown under non-saline conditions, while *SaT58* plant height was considerably more reduced (77.3%). In fields under natural salinity conditions the number of panicles was reduced in all lines (Figure 2). In comparison with non-salt conditions, salinized *SaS62* and *SaT58* plants only produced the 82.7 and 75.2% respectively of the expected panicles and, in addition, these contained fewer grains (74.7 and 67.6% respectively). *SaT20* also produced less panicles under salinity conditions (74.7%), but the number of grains in the panicles resulted less affected by the stress (87.2%). The reduction in panicle formation in Bahia plants was moderate in the presence of salt (87.5%), in contrast to a high decrease in the number of grain compared to non-stress conditions (67.7%). Yield was also affected in all lines. *SaT20* and Bahia showed a similar reduction in yield compared to non-stress conditions (70.5% and 70.7%, respectively). Still, *SaT20* had the highest yield of all tested genotypes under salt conditions, showing 118.5% increase referred to Bahia plants.

### Transcriptional changes in root in response to salt stress

**Figure 3:**
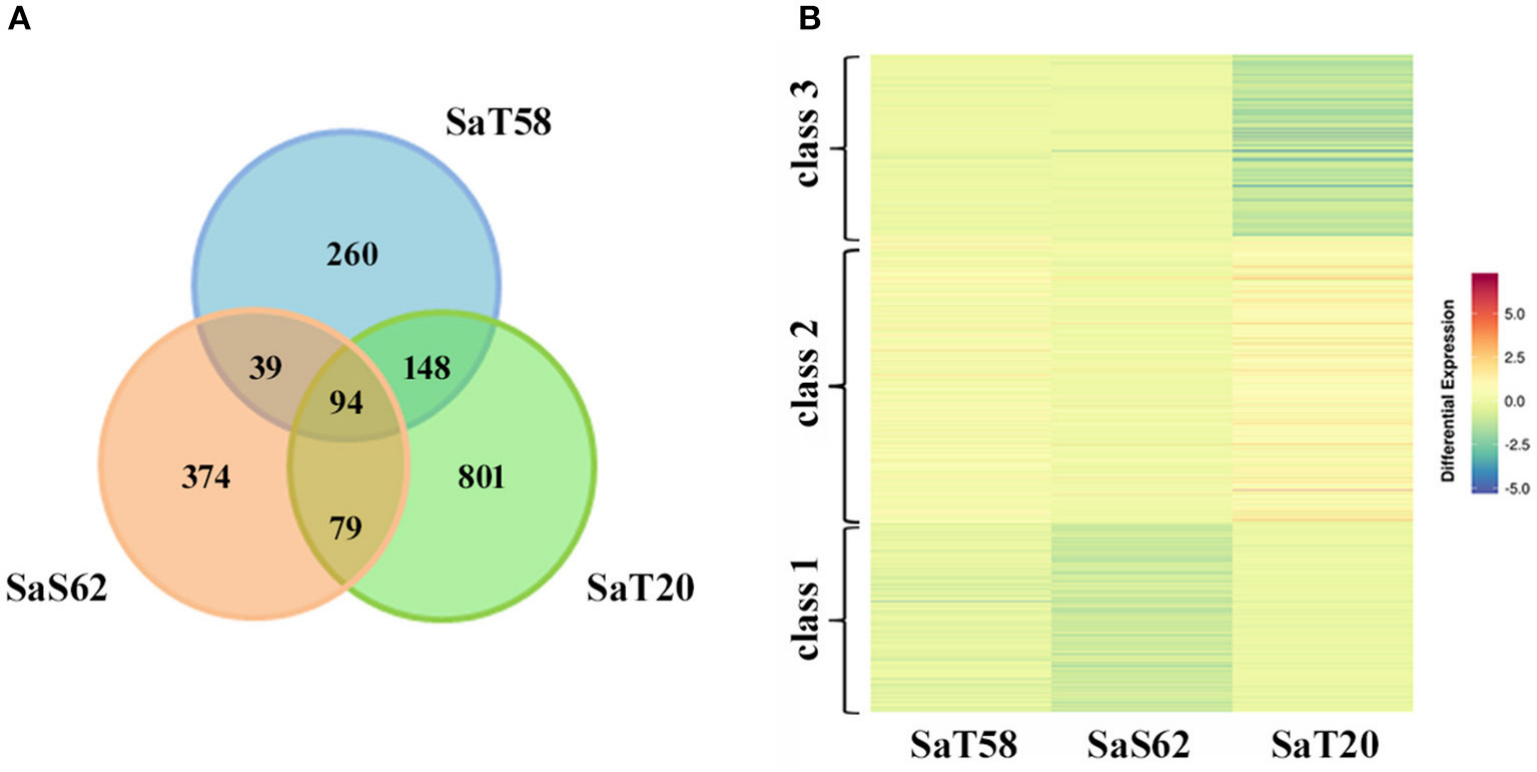
**Venn diagram (A) showing the number of salt-responsive transcripts that changed in roots of *SaT58*, *SaS62*, and *SaT20* compared to parental Bahia**. **(B)** k-means clustering of the expression change pattern across the roots of plants from the *SaT58, SaS62*, and *SaT20* lines treated with 120 mM NaCl.

To identify genes, specific of each mutant line, with altered expression levels due to the ionic stress and avoiding the immediate osmotic effect caused by salt addition, transcriptomic analysis were performed comparing the expression profiles of these independent mutants to control Bahia seedlings grown 4 days in presence of high levels of salt. RNA was extracted from roots and gene expression profiling was analyzed by microarray hybridization using a custom rice transcriptomic arrays. Genes for which all the replicate spots showed differential expression of 1.5 fold (−0.7 > *log*_2_*(Smp/Ctr)* > 0.7 and *P-value* < 0.01) were considered as differentially expressed. The largest number of differentially expressed genes was observed in *SaT20*, with a total of 1122 transcripts of which 801 were specific of *SaT20* (Figure 3A). Ninety-four transcripts were shared between the three mutants (Figure 3).

As an exploratory overview, we first compared the three transcriptomic data and cluster the expression change pattern across the three mutants by k-means clustering implemented in platform CARMO (Comprehensive Annotation of Rice Multi-Omics data) (Wang et al., 2015, http://bioinfo.sibs.ac.cn/carmo/). Differential expressed genes were clustered into three groups (Figure 3B). Functional annotation according to Gene Ontology (GO) and an enrichment analysis, implemented in CARMO, with a significant GO term *p* < 0.05, revealed that class 1 and 3 include genes involved in ion transport and transmembrane transport (Table 2, Table S2). Most of these genes were down-regulated in the mutant plants compared to Bahia plants (Table S3). Class 2 represents stress-responsive genes that are induced by the salinity conditions. Class 2 also comprises genes involved in oxidation-reduction processes as well as lipid and secondary metabolic processes. Most of these genes are up-regulated in the mutant plants in the presence of salt (Table S3).

**Table 2:** **GO term enrichment analysis on differentially expressed genes from the expression change pattern analysis across the roots of plants from the *SaT58, SaS62*, and *SaT20* lines treated with 120 mM NaCl**.

**GO term**	**Biological proccess**	**class 1**	**class2**	**class3**
GO:0055085	Transmembrane transport	14		
GO:0006811	Ion transport	9		9
GO:0006869	Lipid transport		8	
GO:0055114	Oxidation-reduction process		77	
GO:0006629	Lipid metabolic process		40	
GO:0019748	Secondary metabolic process		40	
GO:0006950	Response to stress		144	
GO:0009628	Response to abiotic stimulus		84	
GO:0009651	Response to salt stress	10	19	
GO:0009607	Response to biotic stimulus		48	
GO:0006952	Defense response			14
GO:0006979	Response to oxidative stress		22	9
GO:0046686	Response to cadmium ion	10	20	
GO:0007154	Cell communication		15	

Microarray data from each of the mutants were analyzed individually in detail. Functional classification and GO enrichment analysis of the differential expressed genes were performed using the GO analysis platform AgriGO (Du et al., 2010; http://bioinfo.cau.edu.cn/agriGO) and the Plant GOSlim Assignment of Rice Proteins from the Rice Genome Annotation Project (http://rice.plantbiology.msu.edu). Biological process classification of the differentially expressed genes revealed that the expression profiles differed considerably between the studied genotypes. Several transcripts appeared to be differentially regulated in more than one mutant line and in some cases with different pattern of expression. According to GO assignation, several differentially expressed genes could be included in more than one category corresponding to different biological processes. Transport activity, metabolic process and response to stress appear to be the main biological process categories in the classification of the differential expressed genes (Table 3).

**Table 3:** **Distribution of transcripts significantly regulated in salt-stressed roots of *SaT58, SaS62, SaT20* according to biological process categories**.

**GO term**	**Biological process**	**number of genes involved**	**SaT58 up-**	**SaT58 down-**	**SaS62 up-**	**SaS62 down-**	**SaT20 up-**	**SaT20 down-**
**TRANSPORT**								
GO:0006811	Ion transport	23	1	3	0	12	0	10
GO:0006869	Lipid transport	19	7	1	0	5	9	2
**METABOLIC PROCESS**								
GO:0055114	Oxidation reduction	124	36	5	16	21	50	31
GO:0006629	Lipid metabolic process	66	30	5	5	14	25	12
GO:0019748	Secondary metabolic process	55	26	1	14	7	32	6
**RESPONSE TO STRESS**								
GO:0009628	Response to abiotic stress	151	43	11	17	29	56	35
GO:0009607	Response to biotic stress	85	31	7	4	16	26	19
GO:0006952	Defense response	43	14	1	3	8	14	13
GO:0006979	Response to oxidative stress	38	15	3	3	5	15	9
GO:0006091	generation of precursor metabolites and energy	36	5	3	8	10	12	13

### Transcripts associated with transport activity

**Figure 4:**
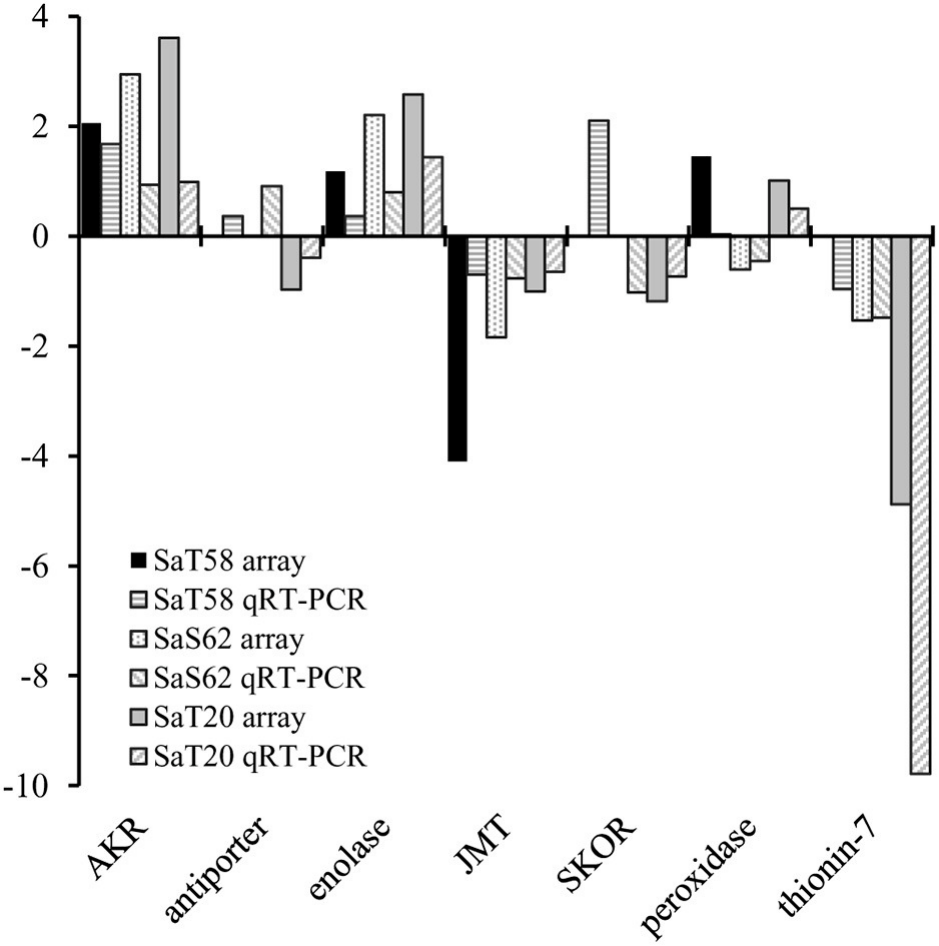
**Comparison between gene expression in *SaT58*, *SaS62*, and *SaT20* roots of plants grown under saline conditions analyzed by qRT-PCR and microarrays and expressed as Log2(mutant/Bahia)**. Genes analyzed were: Monovalent cation:oxidoreductase- aldo/keto reductase family protein (aldo/keto reductase) (AKR) (LOC_Os04g27060), proton antiporter-2 (LOC_Os11g42790), enolase (LOC_Os06g04510), jasmonate O-methyltransferase (JMT) (LOC_Os06g21760), potassium channel SKOR (LOC_Os04g36740), peroxidase precursor (LOC_Os07g48010), thionin-7 (LOC_Os06g32160).

Genes coding for proteins with transport activity include transcripts corresponding to ion transport or lipid-transfer proteins (Table 3). Ion transport showed to be repressed in the three mutants compared to Bahia (Table 4). Several transcripts involved in K^+^ transport were found to be differentially regulated, particularly in *SaT20* and *SaS62*, the two most tolerant lines. *SKOR*, presumably involved in potassium release into the xylem sap toward the shoots (Figure 4) (Gaymard et al., 1998) or *ATCHX*, a member of a gene family predicted to encode Na^+^,K^+^/H^+^ antiporters (Cellier et al., 2004; Sze et al., 2004), were found to be down-regulated in *SaT20* and *SaS62* respectively. In contrast, *AKT1*, encoding a potassium channel involved in the up-take of K^+^ by the cell and probably in nutrition (Lagarde et al., 1996; Li et al., 2014), showed to be up-regulated in *SaT58*. Three other genes coding for other potassium transporters were also down regulated at least in *SaT20* or *SaS62* mutant lines. Only two specific genes coding for sodium/hydrogen exchangers, LOC_Os11g42790 and LOC_Os12g44360, showed differential levels of expression in *SaT20* plants compared to Bahia plants.

**Table 4:** **Differentially regulated genes involved in ion transport and calcium signal transduction, based on microarray analyses, in salt-stressed roots of tolerant mutant lines compared to sensitive parental Bahia**.

	**Putative function**	**SaT58**	**SaS62**	**SaT20**
**ION TRANSPORT**				
LOC_Os04g36740	Potassium channel SKOR			−1.19(6.0E-05)
LOC_Os04g58620	Potasium efflux antiporter protein		−0.97(1.6E-03)	
LOC_Os09g12790	Potassium channel protein		−0.70(2.3E-04)	
LOC_Os12g42200	ATCHX		−0.72(7.1E-05)	
LOC_Os08g10550	Potassium transporter			−0.98(1.2E-04)
LOC_Os01g45990	Potassium channel AKT1	0.78(1.0E-04)		
LOC_Os08g32650	Cation efflux family protein	−0.81(1.0E-04)		
LOC_Os12g44360	Sodium/hydrogen exchanger 7			−1.1(9.5E-04)
LOC_Os11g42790	Transporter, monovalent cation:proton antiporter-2 family			−0.97(7.5E-05)
LOC_Os02g08018	Calcium-transporting ATPase 9, plasma membrane-type	−1.08(2.0E-05)	−0.76(6.5E-05)	
LOC_Os03g17310	Calcium-transporting ATPase, endoplasmic reticulum-type		−1.12(2.8E-05)	
LOC_Os07g33790	Glutamate receptor 3.4 precursor			−1.09(2.6E-03)
LOC_Os02g54760	Cyclic nucleotide-gated ion channel 14		−0.77(2.0E-03)	
LOC_Os01g74110	Similar to Zinc transporter 2 precursor (ZRT/IRT-like protein 2)	−0.74(1.2E-04)	−1.48(2.0E-05)	−0.84(2.2E-04)
LOC_Os06g46310	Metal transporter Nramp6		−0.86(5.1E-05)	
LOC_Os07g15370	Metal transporter Nramp6			−1.19(3.6E-05)
LOC_Os01g68040	CorA-like magnesium transporter protein		−1.37(5.7E-04)	
LOC_Os05g47980	ATP synthase, putative		−2.24(2.5E-06)	
LOC_Os11g06890	Vacuolar ATP synthase, putative		−1.21(1.4E-05)	
LOC_Os04g45520	Integral membrane protein			−1.75(4.7E-06)
LOC_Os04g59020	Integral membrane protein			−0.99(3.0E-05)
LOC_Os01g56420	Ctr copper transporter family protein		−0.71(2.1E-03)	
LOC_Os06g36450	Ferroportin1 domain containing protein			−0.7(9E-05)
**CALCIUM SIGNAL TRANSDUCTION**				
LOC_Os07g48780	OsCam1-2 - Calmodulin		−1.66(1.6E-05)	
LOC_Os11g04560	Calmodulin-like protein 1	0.80(3.8E-05)		0.98(5.3E-05)
LOC_Os08g02420	OsCML7−Calmodulin-related calcium sensor protein			−0.71(9.0E-05)
LOC_Os11g44630	Calmodulin binding protein		−0.89(1.1E-04)	−1.02(1.1E-03)
LOC_Os03g32160	Calmodulin binding protein		−0.70(1.2E-04)	
LOC_Os01g69910	Calmodulin-binding transcription activator		−0.82(8.8E-05)	
LOC_Os01g51840	IQ calmodulin-binding motif family protein			−0.82(1.8E-03)
LOC_Os02g19640	IQ calmodulin-binding motif family protein	0.79(4.3E-05)		
LOC_Os01g67090	IQ calmodulin-binding motif domain containing protein	1.11(3.0E-05)	0.72(1.1E-04)	0.91(5.5E-05)
LOC_Os01g51420	Calcineurin B	−0.99(1.2E-04)	−0.89(5.7E-05)	−0.70(2.4E-04)
LOC_Os03g33570	Calcineurin B-like protein 8			−0.86(1.0E-04)
LOC_Os10g41510	Calcineurin B		−0.81(4.5E-05)	
LOC_Os04g47300	CAMK_CAMK_like.26			1.01(1.2E-03)
LOC_Os03g22050	CAMK_KIN1/SNF1/Nim1_like.16	−0.72(8.0E-05)		
LOC_Os06g35160	CAMK_KIN1/SNF1/Nim1_like.26−SOS2-like			−0.86(6.9E-05)
LOC_Os12g03810	CAMK_KIN1/SNF1/Nim1_like.37			0.95(1.8E-04)
LOC_Os11g03970	CAMK_KIN1/SNF1/Nim1_like.5			1.20(6.4E-05)
LOC_Os01g66890	BTBZ1−Bric-a-Brac, Tramtrack, and Broad Complex BTB domain with TAZ zinc finger and Calmodulin-binding domains			−0.72(4.6E-04)

The expression levels are indicated as log_2_(mutant line/Bahia). P-values are indicated in parentheses.

Cytosolic Ca^2+^ concentration increases rapidly under high salt stress and spontaneously initiates a signaling cascade that gives rise to the plant response (Sanchez-Barrena et al., 2005). Various Ca^2+^ transporters are known to participate actively to maintain the Ca^2+^ cytosolic balance (Singh et al., 2014). The microarray analysis performed in this study revealed the down-regulation in the three mutants of a series of genes possibly involved in Ca^2+^ transport between membranes. Two putative calcium-transporting ATPase coding genes were found to be down-regulated in *SaS62* and *SaT58*. Similarly, a gene encoding a glutamate receptor was down-regulated in *SaT20* plants.

Calcium binding was also affected in the mutants due to the presence of salt. Transcripts coding for calmodulins and calmodulin binding proteins, were found to be down-regulated in the *SaT20* and *SaS62* plants compared to parental plants. Additionally, genes coding for calcium/calmodulin-dependent protein kinase (CAMK) and calcineurin B-like proteins were differentially expressed. CAMKs, with kinase capacity, and calcineurin B-like proteins, with phosphatase capacity, are regulated by the calcium/calmodulin complex. One of the *CAMK* genes, LOC_Os06g35160, that appears to be down-regulated in *SaT20*, corresponds to a protein with similarity to SOS2, a kinase that conferees tolerance to salt (Kim et al., 2013). Additionally, with two exceptions, genes that code for calcineurin B-like proteins, that function in sensing cytosolic Ca^2+^ concentration by binding to Ca^2+^, were found to be down-regulated in the *SaT20* and *SaS62* plants compared to parental plants when grown under saline conditions. Ion transport activity, other than Na^+^ and K^+^ transport, has been described previously to be affected in the salt response of plants (Maathuis, 2006). Several transcripts corresponding to metal transport associated proteins were down-regulated in the mutants compared to sensitive parental particularly, in *SaT20* and *SaS62* plants. Among them *NRAMP* genes in plants encode intracellular metal transporters with capacity to transport both the metal nutrient Fe and the toxic metal cadmium (Thomine et al., 2000; Cailliatte et al., 2009).

Transport group also included 18 differentially expressed genes coding for members of the lipid-transfer protein (LTPL) family, which could be found differentially expressed in plants of the three mutants under salinity treatment. Of all *LTPL* genes found, 11 were up-regulated in *SaT20* and *SaT58* plants. On the contrary, 5 *LTPL* genes were down-regulated in *SaS62* line (Table S4).

### Transcripts associated with metabolic process

Transcripts related to metabolic processes included genes coding for proteins participating in oxidation-reduction process, lipid metabolism, or secondary metabolism. Genes encoding proteins with oxidoreductase activity like peroxidases, cytochrome P450 and oxidoreductase, aldo/keto reductase are included in this group. Genes coding for proteins involved in lipid metabolic process were also abundant, displaying different patterns of expression (Table S4). In *SaT20* and *SaT58*, the number of up-regulated genes related to lipid metabolism was higher than the number of down-regulated genes but on the contrary, it was lower in *SaS62* plants (Table 3). Phospholipids are involved in stress signaling pathways in the response of rice to salt. In salinized *SaS62* plants, transcript levels of phospholipases D, which regulates the formation of phospholipid-based signaling molecules (Kumar et al., 2013), were lower than those found in Bahia plants (Table S4). Secondary metabolic process was also affected, as indicated by the activation of several genes. Up to 17 transcripts coding for glutathione S-transferase and 12 putative cytochrome P450 were differentially expressed, most of them showing higher expression levels in the three mutants than in Bahia plants. This category also comprises genes corresponding to proteins involved in the synthesis of secondary wall, like two laccases, or two caffeoyl-CoA O-methyltransferases, which were also up-regulated.

### Transcripts associated with the stress response

The salt-induced stress in treated plants was clearly reflected in their expression profiles and the group of genes involved in the response to stress was the most abundant (Table 3). Transcripts representing osmoprotectans, molecules involved in detoxification of reactive oxygen species (ROS) and stabilization of quaternary structure of proteins, were also found with different levels of abundance compared to Bahia. Three genes involved in the synthesis of trehalose, a compound that acts as osmoprotectan (Garg et al., 2002), were found to be down-regulated in *SaS62* plants compared to the sensitive parental. Four thaumatin and up to eight glycosyl hydrolase genes were up-regulated in the three mutant plants. Several defense genes were also up-regulated, probably as a general mechanism of defense of the plant despite their function is not directly linked to the abiotic stress response. Furthermore, this defense group includes genes coding for NBS-LRR disease resistance proteins, pathogenesis-related proteins or terpene synthases. One gene coding for a cysteine proteinase inhibitor was also up-regulated (Table S4).

Jasmonic acid (JA) and its methyl ester, methyl jasmonate (MeJA), are known to be primary intracellular transducers of stress response (Ismail et al., 2014). It is remarkable that a gene coding for a 12-oxophytodienoate reductase, probably involved in JA synthesis is up-regulated in *SaT20* and *SaT58*, while another gene corresponding to a jasmonate-O-methyltransferase is down regulated in the three tolerant mutants (Figure 4).

### Ion transport in leaves of *SaT20* plants in response to salt stress

**Figure 5:**
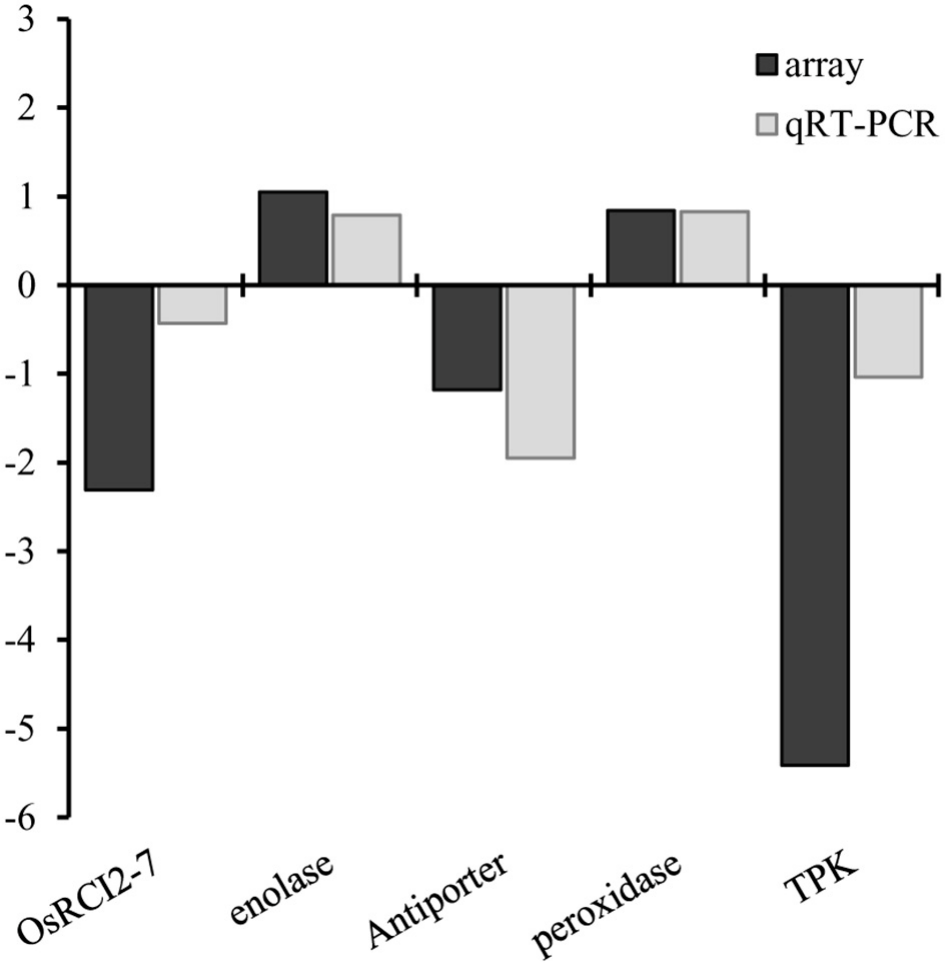
**Comparison between gene expression in *SaT20* leaves of plants grown under saline conditions analyzed by qRT-PCR and microarrays and expressed as Log2(mutant/Bahia)**. Genes analyzed were: OsRCI2-7 (LOC_Os05g03130), enolase (LOC_Os06g04510), monovalent cation:proton antiporter-2 (LOC_Os11g42790), peroxidase precursor (LOC_Os07g48010), and tyrosine protein kinase domain containing protein (TPK) (LOC_Os03g06330).

Accumulation of Na^+^ in *SaT20* leaves when plants were cultured in the presence of high salt concentration led us to investigate the presence of transcripts corresponding to ion transporters in leaves of *SaT20* plants. Expression analysis of leaves from *SaT20* plants grown under salt conditions was performed by microarray hybridization (Figure 5). We found 22 transcripts associated to ion transport, of which 18 were down-regulated, showing the same tendency as in the roots (Table S4). Among these genes, 9 genes could be involved in K^+^ transport (Table 5). Genes coding for OsHKT2;4, a high-affinity Na^+^ and K^+^ transporter (Sassi et al., 2012; Rosas-Santiago et al., 2015) and *SKOR* were found to be down-regulated in leaves of *SaT20* in response to salt. It is remarkable that a gene coding for a chloride transporter was found to be down-regulated in *SaT20* leaves, where Cl^−^ content was found to be high.

**Table 5:** **Differentially regulated genes involved in ion transport in salt-stressed leaves of *SaT20* mutant compared to sensitive parental Bahia**.

**Locus**	**Putative function**	**Folding change**
LOC_Os06g14030	Potassium channel SKOR	−0.97(1.2E-04)
LOC_Os06g48800	OsHKT2;4 - K^+^/Na^+^ transporter	−1.24(3.4E-05)
LOC_Os06g15910	Potassium transporter	−1.48(8.9E-06)
LOC_Os02g49760	Potassium transporter	−0.76(4.6E-05)
LOC_Os04g32920	Potassium transporter	0.85(5.2E-05)
LOC_Os11g42790	Transporter. monovalent cation:proton antiporter-2 family	−1.18(1.6E-05)
LOC_Os07g47100	Transporter. monovalent cation:proton antiporter-2 family	−1.33(1.8E-05)
LOC_Os08g42110	Similar to cation cation antiporter.	0.94(2.1E-04)
LOC_Os03g45370	Sodium/calcium exchanger protein	−0.98(5.7E-05)
LOC_Os03g55100	Cyclic nucleotide-gated ion channel 2, putative	−0.94(1.2E-04)
LOC_Os04g32050	Iron-phytosiderophore transporter	−0.88(2.7E-05)
LOC_Os05g31750	Annexin. putative. expressed	−0.75(5.5E-05)
LOC_Os02g14980	Calcium-binding EF hand family protein	−0.72(1.0E-04)
LOC_Os11g05010	Heavy-metal-associated domain-containing protein	0.77(1.2E-03)
LOC_Os08g10630	Similar to Zinc transporter ZIP1 (Fragment)	1.06(1.4E-04)
LOC_Os01g03914	Cation efflux family protein	−0.72(2.3E-05)
LOC_Os02g38020	Inorganic phosphate transporter 2-1, chloroplast precursor	−0.85(5.0E-05)
LOC_Os03g06520	Sulfate transporter	−1.17(2.1E-05)
LOC_Os03g27040	Heavy metal-associated domain containing protein	−0.82(3.7E-05)
LOC_Os03g48000	CorA-like magnesium transporter protein	−0.83(3.8E-05)
LOC_Os06g48060	ABC transporter, ATP-binding protein	−1.01(3.2E-04)
LOC_Os12g25200	Chloride transporter, chloride channel family	−0.89(2.4E-04)

The expression levels in leaves are indicated as log_2_(SaT20/Bahia). P-values are indicated in parentheses.

## Discussion

From a physiological screen for salt tolerance of gamma-irradiated M2 plants, three rice mutants that showed increased tolerance at seedling stage were identified. Rice plants are considered to be highly sensitive to salt at young seedling stage (Flowers and Yeo, 1981) and from an agronomic point of view, salt tolerance at this moment is of importance in saline environments as crop establishment is fundamentally determined during the earliest stages of development. We first analyzed the response of the newly generated mutant lines in salinized fields. Plants were exposed to salinity during whole life cycle and yield was thus compared to plants grown under non-salt conditions. Among the three mutant lines, *SaT20* stands out for its best yield performance under both salt and non-salt conditions. *SaT20* plants accomplished 118% yield increase over wild type Bahia plants grown in both conditions. In these mutants, yield reduction caused by salinity was similar to that produced in Bahia (71.1 and 70.7%, respectively compared to non-salt conditions) indicating that tolerance at early stage does not necessarily imply an increase in yield. This is in accordance with the effect of salinity on the other two mutant lines, which showed higher reduction in yield than wild type Bahia. Reductions of the two pivotal parameters determining final yield, tillering and number of filled grains per panicle are among the main effects caused by salinity stress during the vegetative stage (Zeng and Shannon, 2000). Salt tolerant mutant lines exhibited an opposite response to sensitive Bahia parental when grown in fields under salt conditions. Mutant lines showed higher reduction in the number of panicles than Bahia plants, but the number of filled grains in these panicles was less affected by salinity. Thus, despite the moderate decrease in the tiller production in Bahia plants, reduction in yield resulted similar to that of the mutant lines.

The response of plants to toxicity and osmotic disturbances caused by Na^+^ includes transcript regulation of numerous genes involved in ion transport. Cation transport contributes to maintain ion homeostasis under salt stress by exclusion, redistribution, and compartmentalization of ions. The response of *SaT20* plants to the presence of high concentration of salt was different to that exhibited by Bahia, *SaS62*, and *SaT58* plants. Among the three induced mutants, *SaT20* plants showed the healthiest aspect when grown in the presence of high concentration of salt, with green leaves and no signs of cellular damage. *SaT20* plants accumulated Na^+^ and Cl^−^ in leaves without signals of injury, an observation that may indicate the occurrence of a strong compartmentalization component in this mutant. Sequestration of Na^+^ into vacuoles is an efficient mechanism of protection of plants against ion toxicity caused by a high accumulation of Na^+^ and Cl^−^ in the cytosol (Hasegawa et al., 2000; Munns and Tester, 2008). In the current work, several molecular factors mostly cation transporters or antiporters were found to be transcriptional regulated in *SaT20* leaves suggesting that some of them could be involved in Na^+^ compartmentalization into the vacuoles. Sequestration of Na^+^ into the vacuoles of the leaf cells, in addition to keep low cytosolic Na^+^, promotes high levels of K^+^, a crucial factor for salt tolerance (Blumwald, 2000; Horie et al., 2012). Under salinity conditions, K^+^ accumulation in the cytosol has been found to be important for normal cellular metabolism. K^+^ up-take has a double function: To maintain ion homeostasis and to contribute to nutrition for adequate plant growth and development (Lagarde et al., 1996). In this sense, K^+^/ Na^+^ ratio can be used as an indicator of the plant response to the stress. *SaS62* showed higher K^+^/ Na^+^ ratio than Bahia as indicative of salt tolerance, while ratio in *SaT58* remained similar to Bahia. In the case of *SaT20*, low K^+^/ Na^+^ ratio found is compatible with the idea that this mutant possesses an effective mechanism for sodium compartmentalization. Cation transport contributes to keep low level of Na^+^ and a favorable K^+^/Na^+^ ratio by redistribution and compartmentalization of ions.

As revealed by the gene expression analyses, ion transport is strongly reduced in the three tolerant mutants grown under salinity conditions. Several transcripts related to potassium transport were found to be down-regulated in roots of *SaT20* and *SaS62* plants in the response to salt compared to Bahia plants. Transcripts corresponding to SKOR, presumably involved in potassium release into the xylem sap toward the shoots (Gaymard et al., 1998), showed lower levels in roots and leaves of *SaT20* than in Bahia. Expression of ATCHX, predicted to encode Na^+^, K^+^/H^+^ antiporters (Cellier et al., 2004; Sze et al., 2004), was found to be down-regulated in *SaS62* roots and *SaT20* leaves. In contrast, expression of potassium channel *AKT1*, implicated in cellular up-take of K^+^ (Li et al., 2014; Lagarde et al., 1996), was up-regulated in *SaT58*, probably mediating K^+^ loading to the xylem or apoplastic space under salinity stress. Thus, this mutant appears to possess an additional mechanism of response to salt compared to *SaT20* and *SaS62* plants.

Molecular factors involved in cellular Na^+^ uptake and translocation system from roots to the shoots have been described previously (Maathuis, 2006). Expression analysis by microarray hybridization revealed few Na^+^ transporters, non-selective cation channels, or membrane-potential modulators such as proton pumps, that were differentially expressed in response to salt stress in tolerant mutants and sensitive Bahia plants. At least two genes putatively involved in sodium/hydrogen exchange showed lower levels of transcripts in *SaT20* plants than in Bahia plants.

A large group that stands out in the microarray analysis includes genes related to transport and metabolic process of lipids. While *SaT58* and *SaT20* showed up-regulation of genes in this group compared to Bahia, *SaS62* showed a general down-regulation. Plant LTPs are a family of proteins with the ability to transfer phospholipids between membranes. As a result of numerous studies, LPTs have been associated with several biological processes as defense and adaptation to environmental stresses (Yeats and Rose, 2008). LTPs are thought to be involved in the transfer of lipids through the extracellular matrix for the formation of cuticular wax and, thus, in accordance with their participation in conditions related to desiccation or water stress in plants, as drought, cold and salt stresses (Cameron et al., 2006). In addition, phospholipids seem to play important structural roles during stress inducing a cytosolic Ca^2+^ peak that acts as a signal to promote the defense response (Kumar et al., 2013). Furthermore, a gene coding for phospholipase D, a regulator of phospholipid-based signaling molecules, was down-regulated in *SaS62*.

Our microarray analysis also revealed that in the three tolerant mutants, salinity induced down-regulation of a gene coding for a jasmonate O-methyltransferase, an enzyme that catalyzes the methylation of JA into MeJA. JA and its methyl ester form, MeJA, are plant volatiles that act as important cellular regulators mediating diverse developmental processes and defense responses and its involvement in salt stress has been previously reported in several species as rice and barley (Kang et al., 2005; Walia et al., 2007). Both compounds, JA and MeJA, have been shown to act as active regulators in an indirect manner. The ectopic expression of *JMT (JA carboxy methyl transferase*) in *Nicotiana attenuate* demonstrated that there is no direct bioactivity of JA and MeJA. The over-expression of *JMT* negatively affected the formation of jasmonoyl-isoleucine (JA-Ile), and the biological activity of MeJA was only apparent when MeJA was converted to JA followed by its conjugation to JA-Ile (Stitz et al., 2011). The down-regulation of *JMT* in the salt tolerant mutant plants in the presence of salt is in accordance with previous reports on other salt tolerant rice genotypes (Cotsaftis et al., 2011).

In conclusion, we compared the response to salt stress of three tolerant mutant lines and their sensitive parental cultivar and observed differences between each individual response. At the physiological level, one of these mutant lines, *SaT20*, showed higher leaf Na^+^ accumulation than Bahia and the other two mutants while displaying the healthiest aspect. The analyses of the transcriptomes reflected two major observations, on one side, identified a profile of common root responses to the presence of high concentration of salt as a part of a general mechanism of tolerance to abiotic stress, in which jasmonate regulation and inhibition of ion transport plays a major role. On the other side, particular and distinctive differences between the responses of the three tolerant mutants were revealed. Transcriptomic data from *SaS62*, a mutant line showing strong salt tolerance phenotype, indicated a down-regulation of K^+^ transport and lipid metabolic process. The analysis of these three salt tolerant mutants, while underlying the differences between the distinct salt tolerant lines, illustrated the complexity of this trait evidencing several alternative mechanisms and plant response to address the challenge of salt stress.

## Author contributions

CD conceived the project, designed research, participated in the generation of mutant lines, data interpretation, and wrote the manuscript. EL performed RNA isolation, microarray hybridization, and expression analysis. MC and EP performed agronomical characterization. JR participated in the expression analysis, manuscript preparation, and revision. MT designed research, participated in manuscript preparation, and revision. All authors read and approved the final manuscript.

### Conflict of interest statement

The authors declare that the research was conducted in the absence of any commercial or financial relationships that could be construed as a potential conflict of interest.

## Abbreviations

CAMK: calcium/calmodulin-dependent protein kinase
JA: jasmonic acid
mRNA: messenger RNA
MeJA: methyl jasmonate
LTPL: lipid-transfer protein
RT-PCR: Real-Time Polymerase Chain Reaction.
