# Supplementary material for: Physiological Basis and Transcriptional Profiling of Three Salt-Tolerant Mutant Lines of Rice
Source: Front Plant Sci. 2016 Sep 28;7:1462. doi: 10.3389/fpls.2016.01462 (PMC5039197; doi:10.3389/fpls.2016.01462)
Supplement: Supplementary file 1 [file Table1.PDF]

Supplementary table S1.- Monthly mean values for maximum and minimum temperature from the meteorological station located at Amposta, near the two experimental fields, during the years 2011 and 2012. (Official data provided by the Institut d'Estadística de Catalunya; http://www.idescat.cat)

		**May**	**June**	**July**	**August**	**September**
**maximum**	**2011**	23.2	25.2	27.2	28.8	27.9
	**2012**	22.2	26.4	27.4	29.9	27.0
**minimum**	**2011**	15.4	18.1	20.3	21.3	18.2
	**2012**	14.8	19.2	19.6	21.5	17.3
