# Supplementary material for: Physiological Basis and Transcriptional Profiling of Three Salt-Tolerant Mutant Lines of Rice
Source: Front Plant Sci. 2016 Sep 28;7:1462. doi: 10.3389/fpls.2016.01462 (PMC5039197; doi:10.3389/fpls.2016.01462)
Supplement: Supplementary file 2 [file Table2.PDF]

Supplementary Table S2.- Number of differentially expressed genes included in the three classes resulted from the transcriptome comparison of roots of plants from the *SaT58*, *SaS62* and *SaT20* lines treated with salt. Number of genes with functional annotation and number of genes that matched GO term with a significance $p<0.05$

	**total genes**	**Annotated genes**	**Matched GO term**
class 1	445	423	100
class 2	678	656	235
class 3	428	425	164
**total genes involved**	**1551**	**1504**	**499**
