# Supplementary material for: Physiological Basis and Transcriptional Profiling of Three Salt-Tolerant Mutant Lines of Rice
Source: Front Plant Sci. 2016 Sep 28;7:1462. doi: 10.3389/fpls.2016.01462 (PMC5039197; doi:10.3389/fpls.2016.01462)
Supplement: Supplementary file 3 [file Table3.PDF]

Supplementary Table S3.- Differentially expressed gene classification according to k-means clustering of the three mutant lines treated with 120 mM NaCl.

Locus	functional annotation	Group	SaT58	SaS62	SaT20
LOC_Os01g01830	OsPOP1 - Putative Prolyl Oligopeptidase homologue	1	-0.71	-0.99	
LOC_Os01g02880	fructose-bisphospate aldolase isozyme, putative	1	-0.84	-1.51	
LOC_Os01g05610	Core histone H2A/H2B/H3/H4 domain containing protein, putative	1	-0.81	-0.82	
LOC_Os01g07760	phospholipase D, putative	1		-0.84	
LOC_Os01g08410	early fruit mRNA, putative	1		-1.14	
LOC_Os01g10210	lachrymatory factor synthase, putative	1		-1.38	
LOC_Os01g10530	expressed protein	1	-0.81		
LOC_Os01g10940	S-adenosylmethionine synthetase 2, putative	1	-0.82		
LOC_Os01g13160	expressed protein	1		-0.72	
LOC_Os01g13560	membrane associated DUF588 domain containing protein, putative	1		-1.23	
LOC_Os01g13680	hypothetical protein	1	-0.95		
LOC_Os01g14950	importin subunit alpha, putative	1	-1.05	-2.55	-0.77
LOC_Os01g15020	lissencephaly type-1-like homology motif, putative	1		-1.09	
LOC_Os01g16010	BCAS2 protein, putative	1		-0.92	
LOC_Os01g19150	CGMC_GSK.3 - CGMC includes CDA, MAPK, GSK3, and CLKC kinases	1		-0.97	
LOC_Os01g31980	MATE efflux family protein, putative	1		-1.29	
LOC_Os01g33160	stress responsive A/B Barrel domain containing protein	1		-0.7	
LOC_Os01g33810	disease resistance protein RPM1, putative	1		-0.78	
LOC_Os01g34610	expressed protein	1		-0.78	
LOC_Os01g35920	conserved hypothetical protein	1		-0.82	
LOC_Os01g36890	DEAD-box ATP-dependent RNA helicase, putative	1		-0.79	
LOC_Os01g36920	DEAD-box ATP-dependent RNA helicase, putative	1		-1.57	
LOC_Os01g37000	carboxyl-terminal peptidase, putative	1	-1.11	-2.86	-1.42
LOC_Os01g38580	beta,beta-carotene 9,10-dioxygenase, putative	1	-0.76		
LOC_Os01g41240	hydrolase, alpha/beta fold family domain containing protein	1		-1.04	
LOC_Os01g46370	lipase class 3 family protein, putative	1		-1.14	
LOC_Os01g47330	ribosomal protein L7/L12 C-terminal domain containing protein	1	-0.75		
LOC_Os01g47430	protein of unknown function DUF1296 domain containing protein	1		-0.85	
LOC_Os01g48330	protein kinase domain containing protein	1		-0.75	
LOC_Os01g48530	expressed protein	1		-0.7	
LOC_Os01g48930	splicing factor-related, putative	1		-1,00	
LOC_Os01g51410	glycine dehydrogenase, putative	1		-1.71	
LOC_Os01g51420	calcineurin B, putative	1	-0.99	-0.89	-0.7
LOC_Os01g51634	myosin-2 heavy chain, non muscle, putative	1		-0.77	-0.76
LOC_Os01g52110	RING finger and CHY zinc finger domain-containing protein 1, putative	1	-0.92		
LOC_Os01g54600	OsWRKY13 - Superfamily of TFs having WRKY and zinc finger domains	1	-0.97		
LOC_Os01g56420	ctr copper transporter family protein, putative	1		-0,71	
LOC_Os01g56910	expressed protein	1		-0.83	
LOC_Os01g58240	OsSub6 - Putative Subtilisin homologue	1		-0.87	-0.81
LOC_Os01g58740	glycerol-3-phosphate dehydrogenase, putative	1		-1.04	
LOC_Os01g59850	GTPase-activating protein, putative	1		-0.79	
LOC_Os01g60340	NTMC2Type1.1 protein, putative	1		-0.73	
LOC_Os01g60360	ubiquitin-conjugating enzyme, putative	1		-0.87	
LOC_Os01g63690	hs1, putative	1	-1.14		
LOC_Os01g65140	peptide transporter PTR2, putative	1	-1.33		
LOC_Os01g65510	OsFBX31 - F-box domain containing protein	1		-0.83	
LOC_Os01g68040	CorA-like magnesium transporter protein, putative	1		-1.37	
LOC_Os01g68740	keratin, type I cytoskeletal 9, putative	1		-1.01	
LOC_Os01g68810	expressed protein	1		-0.79	
LOC_Os01g69200	regulatory protein, putative	1		-0.98	-0.88

LOC_Os01g69270	OsFBO2 - F-box and other domain containing protein	1	-0.85		
LOC_Os01g69910	calmodulin-binding transcription activator, putative	1		-0.82	
LOC_Os01g70960	cytochrome c1-1, heme protein, mitochondrial precursor, putative	1		-1.35	
LOC_Os01g71400	glycosyl hydrolases family 17, putative	1	-1.53	-0.92	
LOC_Os01g73020	mitochondrial import inner membrane translocase subunit Tim16, putative	1		-0.72	
LOC_Os01g73710	RCLEA3 - Root cap and Late embryogenesis related family protein precursor, putative	1		-0.89	
LOC_Os01g73730	RCLEA5 - Root cap and Late embryogenesis related family protein precursor	1		-0.81	
LOC_Os01g74110	metal cation transporter, putative	1	-0.74	-1.48	-0.84
LOC_Os01g74410	MYB family transcription factor, putative	1	-0.72		
LOC_Os02g01160	skp1 family, tetramerisation domain containing protein	1		-0.81	
LOC_Os02g01170	HECT-domain domain containing protein	1	-1.27	-1.85	-0.85
LOC_Os02g01760	diphosphomevalonate decarboxylase family protein	1		-1.03	
LOC_Os02g01770	hypothetical protein	1	-0.71		
LOC_Os02g02320	OsSCP6 - Putative Serine Carboxypeptidase homologue	1		-0.86	
LOC_Os02g02780	protein kinase family protein, putative	1	-0.8		
LOC_Os02g03080	eukaryotic translation initiation factor 3 subunit C, putative	1	-1.28	-0.93	
LOC_Os02g03730	SWIB/MDM2 domain containing protein	1		-0.83	
LOC_Os02g05410	splicing factor 3B subunit 1, putative	1		-1.11	
LOC_Os02g05450	Homeobox domain containing protein	1		-1.26	
LOC_Os02g05480	CGMC_MAPKCMGC_2_SLT2y_ERK.1 - CGMC includes CDA, MAPK, GSK3, and CLKC kinases	1		-0.92	
LOC_Os02g05660	DEAD-box ATP-dependent RNA helicase 35A, putative	1		-1.07	
LOC_Os02g05790	expressed protein	1		-1.06	
LOC_Os02g06290	rhodanese-like domain containing protein, putative	1		-0.85	
LOC_Os02g07790	serine/threonine-protein kinase HT1, putative	1		-0.8	
LOC_Os02g08018	calcium-transporting ATPase 9, plasma membrane-type, putative	1	-1.08	-0.76	
LOC_Os02g09220	cytochrome P450, putative	1		-0.88	
LOC_Os02g10070	citrate synthase, putative	1		-0.85	
LOC_Os02g11060	WD domain, G-beta repeat domain containing protein	1	-0.85		
LOC_Os02g11740	plastidic ATP/ADP-transporter, putative	1	-1.49	-1.5	
LOC_Os02g11820	GTPase-activating protein, putative	1		-0.94	
LOC_Os02g13850	leucine-rich repeat-containing protein kinase family protein, putative	1		-0.72	
LOC_Os02g16730	expansin precursor, putative	1		-0.92	
LOC_Os02g17850	TOM2B, putative	1		-0.89	
LOC_Os02g20280	expressed protein	1		-1,00	
LOC_Os02g20940	CHCH domain containing protein	1		-0.96	
LOC_Os02g20970	C2 domain containing protein, putative	1		-1.1	
LOC_Os02g21900	40S ribosomal protein S7, putative	1		-0.8	
LOC_Os02g24134	Sec1 family transport protein, putative	1		-0.86	
LOC_Os02g30320	drought-induced protein 1, putative	1		-0.82	
LOC_Os02g30410	expressed protein	1		-0.72	
LOC_Os02g32770	cytochrome P450, putative	1		-0.86	
LOC_Os02g36414	transporter family protein, putative	1		-1.03	
LOC_Os02g39160	hydroxymethylbutenyl 4-diphosphate synthase, putative	1		-0.72	
LOC_Os02g39370	tRNA methyltransferase, putative	1		-0.7	
LOC_Os02g39550	calcium-binding mitochondrial protein anon-60Da, putative	1		-0.91	
LOC_Os02g39790	CPuORF9 - conserved peptide uORF-containing transcript	1		-1.22	
LOC_Os02g39970	regulatory subunit, putative	1		-1.39	
LOC_Os02g40730	ammonium transporter protein, putative	1		-1.45	
LOC_Os02g41904	DEF7 - Defensin and Defensin-like DEFL family	1		-0.96	
LOC_Os02g43090	myristoyl-acyl carrier protein thioesterase, chloroplast precursor, putative	1		-0.78	
LOC_Os02g43519	expressed protein	1		-0.75	
LOC_Os02g44220	peroxisomal biogenesis factor 19, putative	1		-0.73	
LOC_Os02g44599	expressed protein	1	-0.74		

LOC_Os02g44730	tetracycline transporter protein, putative	1	-0.82	-1.16	-0.75
LOC_Os02g45240	zinc finger family protein, putative	1		-1.15	
LOC_Os02g47120	region found in RelA/SpoT proteins containing protein	1		-1.01	
LOC_Os02g47220	CPuORF20 - conserved peptide uORF-containing transcript	1		-1.43	
LOC_Os02g48010	nuclear matrix constituent protein 1-like, putative	1		-1.43	
LOC_Os02g48720	mitochondrial carrier protein, putative	1	-1.07	-2.29	
LOC_Os02g50330	RNA-dependent RNA polymerase, putative	1		-1.87	
LOC_Os02g51610	CRAL/TRIO domain containing protein	1		-0.99	
LOC_Os02g53700	DENN domain containing protein	1		-0.9	
LOC_Os02g54760	cyclic nucleotide-gated ion channel 14, putative	1		-0.77	
LOC_Os02g55150	formin, putative	1		-0.84	
LOC_Os02g56600	no apical meristem protein, putative	1		-1.23	-1.04
LOC_Os02g56720	cinnamoyl CoA reductase, putative	1		-0.74	
LOC_Os02g56850	glutathione reductase, putative	1		-1.28	
LOC_Os02g57260	3-ketoacyl-CoA thiolase, peroxisomal precursor, putative	1		-1.57	
LOC_Os03g01910	transcription factor BTF3, putative	1		-0.74	
LOC_Os03g02080	expressed protein	1		-0.84	
LOC_Os03g05640	inorganic phosphate transporter, putative	1	-0.81		
LOC_Os03g10700	expressed protein	1	-1.33		
LOC_Os03g11240	nucleotidyltransferase, putative	1		-0.75	
LOC_Os03g11890	potyvirus VPg interacting protein, putative	1		-0.79	
LOC_Os03g11970	tubulin/FtsZ domain containing protein, putative	1	-1.29	-0.76	
LOC_Os03g12160	leucine-rich repeat family protein, putative	1		-0.87	
LOC_Os03g12670	expressed protein	1	-1.22	-0.95	
LOC_Os03g13560	hydroxyproline-rich glycoprotein family protein, putative	1		-0.74	
LOC_Os03g13790	myb/SANT domain protein, putative	1		-0.96	
LOC_Os03g13870	expressed protein	1	-0.71		
LOC_Os03g14860	G-patch domain containing protein	1	-0.72		
LOC_Os03g15050	phosphoenolpyruvate carboxykinase, putative	1	-0.8	-1.24	
LOC_Os03g15110	expressed protein	1		-0.94	
LOC_Os03g16110	Ser/Thr protein phosphatase family protein, putative	1		-0.96	
LOC_Os03g17120	arginine biosynthesis bifunctional protein argJ 1, putative	1		-1.22	
LOC_Os03g17310	calcium-transporting ATPase, endoplasmic reticulum-type, putative	1		-1.12	
LOC_Os03g17870	metallothionein, putative	1		-1.69	-1.11
LOC_Os03g18220	pyruvate decarboxylase isozyme 2, putative	1	-0.89	-1.86	
LOC_Os03g19340	WD domain, G-beta repeat domain containing protein	1		-0.76	
LOC_Os03g19420	nicotianamine synthase, putative	1	-0.85		
LOC_Os03g19870	ATP binding protein, putative	1		-0.79	
LOC_Os03g20420	alpha-N-arabinofuranosidase A, putative	1		-0.98	
LOC_Os03g21900	uroporphyrinogen decarboxylase, putative	1		-0.73	
LOC_Os03g22050	CAMK_KIN1/SNF1/Nim1_like.16 - CAMK includes calcium/calmodulin depedent protein kinases	1	-0.72		
LOC_Os03g22730	nucleolar protein NOP5-1, putative	1		-0.72	
LOC_Os03g23909	vegetative incompatibility protein HET-E-1, putative	1		-0.76	
LOC_Os03g24220	villin protein, putative	1		-1.09	
LOC_Os03g25030	GDSL-like lipase/acylhydrolase, putative	1	-0.75	-1.54	-0.87
LOC_Os03g25330	peroxidase precursor, putative	1		-0.86	
LOC_Os03g25360	peroxidase precursor, putative	1		-0.8	
LOC_Os03g25440	expressed protein	1		-0.92	
LOC_Os03g25620	LTV1, putative	1		-0.9	
LOC_Os03g27090	MYB family transcription factor, putative	1		-0.76	
LOC_Os03g27840	splicing factor, arginine/serine-rich 16, putative	1		-0.8	
LOC_Os03g31134	retrotransposon protein, putative, Ty1-copia subclass	1		-0.87	
LOC_Os03g31230	MYB family transcription factor, putative	1		-0.86	
LOC_Os03g38740	Dicer, putative	1		-1.01	-0.84

LOC_Os03g39380	expressed protein	1		-0.97	
LOC_Os03g40010	DEK C terminal domain containing protein	1		-0.97	
LOC_Os03g43684	KIP1, putative	1		-0.88	
LOC_Os03g43720	transporter family protein, putative	1		-1.41	
LOC_Os03g44484	DNA-directed RNA polymerase II subunit RPB2, putative	1		-1.08	
LOC_Os03g46710	expressed protein	1	-1.12	-0.99	
LOC_Os03g47790	transposon protein, putative, unclassified	1		-0.84	
LOC_Os03g47830	argonaute, putative	1		-1.07	
LOC_Os03g48560	N-acetylglucosaminyltransferase, putative	1		-1.29	
LOC_Os03g51350	expressed protein	1	-0.81		
LOC_Os03g51970	growth-regulating factor, putative	1		-1,00	
LOC_Os03g52830	FATC domain containing protein	1		-0.98	
LOC_Os03g54220	expressed protein	1		-1.05	
LOC_Os03g55640	OsSigP2 - Putative Type I Signal Peptidase homologue; employs a putative Ser/Lys catalytic dyad	1		-0.87	
LOC_Os03g57110	expressed protein	1		-0.97	
LOC_Os03g57760	25.3 kDa vesicle transport protein, putative	1		-0.94	
LOC_Os03g58030	acetyltransferase, GNAT family, putative	1	-1.05		
LOC_Os03g58700	vacuolar protein sorting-associated protein 35, putative	1		-1.51	
LOC_Os03g60500	conserved hypothetical protein	1	-0.74		
LOC_Os03g61240	expressed protein	1	-0.78		
LOC_Os03g61730	heat shock protein DnaJ, putative	1		-0.84	
LOC_Os03g61760	OsSPL6 - SBP-box gene family member	1		-1.39	
LOC_Os03g63430	proteasome subunit, putative	1		-1.83	
LOC_Os03g64210	T-complex protein, putative	1	-0.79	-2.15	-0.7
LOC_Os04g02070	expressed protein	1		-0.79	
LOC_Os04g04020	protein transport protein Sec24-like, putative	1		-1.05	
LOC_Os04g05570	transposon protein, putative, CACTA, En/Spm sub-class	1	-0.96	-0.89	-0.73
LOC_Os04g10750	inorganic phosphate transporter, putative	1	-0.73		
LOC_Os04g24469	jasmonate-induced protein, putative	1		-0.96	
LOC_Os04g27980	glycosyl hydrolase, putative	1		-1.12	
LOC_Os04g28480	retrotransposon protein, putative, unclassified	1		-1.13	
LOC_Os04g33860	expressed protein	1	-1.11		
LOC_Os04g35250	MONOCULM 1, putative	1	-0.71		
LOC_Os04g35750	expressed protein	1	-0.83	-1.22	
LOC_Os04g35860	T-complex protein 11, putative	1		-1.03	
LOC_Os04g37904	protein phosphatase 2C, putative	1		-1.57	
LOC_Os04g40090	zinc finger, ZZ type family protein	1		-0.77	
LOC_Os04g40500	glycosyl hydrolase family 5 protein, putative	1		-1.05	
LOC_Os04g42520	phosphoribosyl transferase, putative	1		-0.8	
LOC_Os04g42600	polyadenylate-binding protein, putative	1		-0.72	
LOC_Os04g43340	disease resistance RPP13-like protein 1, putative	1		-0.71	
LOC_Os04g44030	PPR repeat domain containing protein, putative	1	-0.89	-1.18	-1.15
LOC_Os04g44110	hydrolase, putative	1		-1.3	
LOC_Os04g46820	LTPL121 - Protease inhibitor/seed storage/LTP family protein precursor, putative	1		-1.08	
LOC_Os04g47180	expressed protein	1		-0.72	
LOC_Os04g49920	sensitivity to red light reduced protein 1, putative	1		-0.77	
LOC_Os04g52354	ribosomal protein S17, putative	1	-1.14	-1.3	-0.84
LOC_Os04g52940	SIT4 phosphatase-associated protein domain containing protein	1		-0.83	
LOC_Os04g53350	expressed protein	1		-1.44	
LOC_Os04g55710	transposon protein, putative, unclassified	1	-0.99		
LOC_Os04g58620	potasium efflux antiporter protein, putative	1		-0.97	
LOC_Os04g59630	prenylcysteine oxidase 1 precursor, putative	1	-0.78	-0.98	
LOC_Os05g03550	MYB family transcription factor, putative	1	-0.79	-1.51	
LOC_Os05g03810	trehalose phosphatase, putative	1		-1,00	

LOC_Os05g04340	CGMC_GSK.6 - CGMC includes CDA, MAPK, GSK3, and CLKC kinases	1		-1.79	
LOC_Os05g05860	retrotransposon protein, putative, unclassified	1	-1.28		
LOC_Os05g07670	solute carrier family 35 member E3, putative	1		-0.75	
LOC_Os05g10754	expressed protein	1		-0.93	
LOC_Os05g12300	expressed protein	1		-0.97	
LOC_Os05g12680	retrotransposon protein, putative, unclassified	1		-0.8	
LOC_Os05g13390	expressed protein	1		-1.12	-0.81
LOC_Os05g13940	retrotransposon protein, putative, unclassified	1		-0.88	
LOC_Os05g14940	transposon protein, putative, unclassified	1		-0.74	
LOC_Os05g16930	protein kinase domain containing protein	1		-0.88	
LOC_Os05g20304	expressed protein	1		-0.75	
LOC_Os05g25180	zinc finger, C3HC4 type family protein	1		-0.75	
LOC_Os05g25450	TKL_IRAK_CrRLK1L-1.3 - The CrRLK1L-1 subfamily has homology to the CrRLK1L homolog	1		-0.72	
LOC_Os05g26914	chaperone protein dnaJ, putative	1		-0.89	
LOC_Os05g26980	hypothetical protein	1		-0.99	
LOC_Os05g27870	expressed protein	1		-0.71	
LOC_Os05g28290	ranBP1 domain containing protein	1		-1.3	
LOC_Os05g33730	gibberellin receptor GID1L2, putative	1	-1.52	-1.14	-1.17
LOC_Os05g34110	homeodomain-related, putative	1		-0.79	
LOC_Os05g34210	expressed protein	1		-1.06	
LOC_Os05g34260	transposon protein, putative, Ac/Ds sub-class	1		-1.54	
LOC_Os05g37170	transcription factor, putative	1	-0.77		
LOC_Os05g38150	amino acid kinase, putative	1		-1.39	
LOC_Os05g39770	aminotransferase, putative	1	-0.7	-1.27	
LOC_Os05g41120	endoplasmic reticulum-Golgi intermediate compartment protein 3, putative	1		-0.81	
LOC_Os05g41550	expressed protein	1	-1.29	-1.92	-0.89
LOC_Os05g43280	MATH domain containing protein	1		-1.26	
LOC_Os05g45460	POEI52 - Pollen Ole e I allergen and extensin family protein precursor	1		-0.75	
LOC_Os05g47660	lipid phosphatase protein, putative	1		-0.75	
LOC_Os05g47670	zinc finger, C3HC4 type domain containing protein	1		-0.94	
LOC_Os05g47940	RCLEA8 - Root cap and Late embryogenesis related family protein precursor	1		-1.66	-0.75
LOC_Os05g47980	ATP synthase, putative	1		-2.24	
LOC_Os05g48410	50S ribosomal protein L21, chloroplast precursor, putative	1		-0.74	
LOC_Os05g48760	protein of unknown function DUF1421 domain containing protein	1		-0.78	
LOC_Os05g48840	expressed protein	1		-1.15	
LOC_Os05g49320	ribosomal protein L7/L12 C-terminal domain containing protein	1	-0.87		
LOC_Os05g49840	phospholipase, putative	1		-0.76	
LOC_Os05g50370	dnaJ domain containing protein	1		-0.88	
LOC_Os05g50550	polyprenyl synthetase, putative	1		-0.73	
LOC_Os05g51150	RNA polymerase sigma factor, putative	1		-0.99	
LOC_Os06g01660	integral membrane protein DUF6 domain containing protein	1	-0.79	-1.12	
LOC_Os06g04690	OsFBX184 - F-box domain containing protein	1		-0.97	
LOC_Os06g04880	serine threonine kinase, putative	1		-0.86	
LOC_Os06g07000	OsFBL28 - F-box domain and LRR containing protein	1		-0.71	
LOC_Os06g15690	no apical meristem protein, putative	1		-1.08	
LOC_Os06g16060	zinc finger, C3HC4 type domain containing protein	1	-1.27		
LOC_Os06g16640	carboxyl-terminal peptidase, putative	1	-1.29	-2.7	-1.62
LOC_Os06g19960	aconitate hydratase protein, putative	1		-1,00	
LOC_Os06g21760	jasmonate O-methyltransferase, putative	1	-4.1	-1.84	-1.01
LOC_Os06g22070	mitochondrial glycoprotein, putative	1		-0.91	
LOC_Os06g25294	60S ribosomal protein L18a-1, putative	1		-0.83	
LOC_Os06g40460	expressed protein	1		-1.07	
LOC_Os06g41990	M16 domain containing zinc peptidase, putative	1		-1.15	
LOC_Os06g43270	protein kinase domain containing protein	1		-0.82	

LOC_Os06g43410	cytochrome P450, putative	1		-1.61	
LOC_Os06g43670	Leucine Rich Repeat family protein	1		-0.77	
LOC_Os06g43790	transcription initiation factor TFIID subunit 1, putative	1	-0.73	-0.85	
LOC_Os06g45360	peptidase, M24 family protein, putative	1		-1.73	
LOC_Os06g46310	metal transporter Nramp6, putative	1		-0.86	
LOC_Os06g47580	REV1, putative	1		-0.79	
LOC_Os06g47590	AP2 domain containing protein	1	-1.37		
LOC_Os06g48950	auxin response factor 19, putative	1		-0.79	
LOC_Os06g50679	expressed protein	1	-0.74	-0.9	-0.82
LOC_Os06g51380	ROOT HAIRLESS 1, putative	1		-1.03	
LOC_Os07g02140	flavin-containing monooxygenase family protein, putative	1	-0.88		
LOC_Os07g05390	expressed protein	1		-0.97	
LOC_Os07g06950	ubiquitin carboxyl-terminal hydrolase, putative	1		-0.75	
LOC_Os07g07000	syntaxin, putative	1		-1.78	
LOC_Os07g07860	LTPL76 - Protease inhibitor/seed storage/LTP family protein precursor	1		-0.99	
LOC_Os07g10250	DEAD-box ATP-dependent RNA helicase 52B, putative	1	-1.3	-1.05	
LOC_Os07g10610	expressed protein	1		-0.76	
LOC_Os07g15020	conserved hypothetical protein	1		-0.95	
LOC_Os07g25460	ankyrin repeat domain containing protein	1		-0.8	
LOC_Os07g25680	protein kinase domain containing protein	1	-0.77	-1.58	
LOC_Os07g26550	expressed protein	1		-0.92	
LOC_Os07g27140	AT hook motif family protein	1		-0.8	
LOC_Os07g28940	ATGRIP/GRIP, putative	1		-0.96	
LOC_Os07g29560	conserved hypothetical protein	1	-1.87		
LOC_Os07g30640	ubiquitin fusion protein, putative	1	-1.05		
LOC_Os07g31460	peptide-Nasparagine amidase, putative	1		-0.82	
LOC_Os07g33560	cytochrome P450, putative	1		-0.85	
LOC_Os07g33860	60S ribosomal protein L44, putative	1		-0.76	
LOC_Os07g33997	60S ribosomal protein L44, putative	1		-0.77	
LOC_Os07g36940	eukaryotic translation initiation factor 4G, putative	1		-0.82	
LOC_Os07g37290	hypothetical protein	1	-0.91		
LOC_Os07g37780	ribosomal protein S17, putative	1	-1.28	-0.98	-0.73
LOC_Os07g38360	expressed protein	1	-1.14	-1.15	
LOC_Os07g38570	expressed protein	1	-0.73		
LOC_Os07g38860	OsGH3.10 - Probable indole-3-acetic acid-amido synthetase	1	-1.02		
LOC_Os07g38910	histidine kinase, putative	1		-1.06	
LOC_Os07g39290	ruvB-like, putative	1		-0.78	
LOC_Os07g42220	expressed protein	1	-1.35		
LOC_Os07g42960	phospho-2-dehydro-3-deoxyheptonate aldolase, chloroplast precursor, putative	1		-0.85	
LOC_Os07g43510	40S ribosomal protein S9, putative	1		-1.28	
LOC_Os07g43670	ribonuclease T2 family domain containing protein	1		-0.96	
LOC_Os07g44110	cytochrome P450 72A1, putative	1	-0.76	-1.26	-0.82
LOC_Os07g44780	GDSL-like lipase/acylhydrolase, putative	1		-0.89	
LOC_Os07g45080	expressed protein	1	-0.99	-1.82	-0.72
LOC_Os07g45330	expressed protein	1		-0.93	
LOC_Os07g48410	RNA-binding zinc finger protein, putative	1		-0.73	
LOC_Os07g48450	no apical meristem protein, putative	1	-0.83		
LOC_Os07g48460	stress responsive protein, putative	1	-1.3		
LOC_Os07g48780	OsCam1-2 - Calmodulin	1		-1.66	
LOC_Os07g49200	membrane associated DUF588 domain containing protein, putative	1	-0.86		
LOC_Os08g01610	DUF250 domain containing protein, putative	1		-0.99	
LOC_Os08g01930	KH domain-containing protein, putative	1	-0.8	-1.07	
LOC_Os08g03310	zinc finger family protein, putative	1	-0.99	-0.81	-0.74
LOC_Os08g05280	oxidoreductase family, NAD-binding Rossmann fold containing protein	1	-0.92		

LOC_Os08g06840	expressed protein	1	-0.77		
LOC_Os08g07370	RGH1A, putative	1		-0.77	
LOC_Os08g07380	retrotransposon protein, putative, unclassified	1		-1.86	
LOC_Os08g07774	disease resistance protein RPM1, putative	1		-1.03	
LOC_Os08g08050	retrotransposon protein, putative, unclassified	1		-0.73	
LOC_Os08g08586	expressed protein	1		-0.79	
LOC_Os08g08970	Cupin domain containing protein	1	-1.15	-2,00	
LOC_Os08g08980	cupin domain containing protein	1	-0.92	-1.81	
LOC_Os08g08990	Cupin domain containing protein	1		-1.21	0.77
LOC_Os08g11720	expressed protein	1		-0.93	
LOC_Os08g16914	expressed protein	1		-0.84	
LOC_Os08g17400	WRKY DNA-binding domain containing protein	1		-1.12	
LOC_Os08g19480	retrotransposon protein, putative, Ty3-gypsy subclass	1		-1.01	
LOC_Os08g23780	glycosyl transferase 8 domain containing protein, putative	1	-0.77		
LOC_Os08g25864	expressed protein	1		-1.39	
LOC_Os08g30930	expressed protein	1	-1.26		
LOC_Os08g32100	transposon protein, putative, CACTA, En/Spm sub-class	1		-1.71	-0.96
LOC_Os08g32650	cation efflux family protein, putative	1	-0.81		
LOC_Os08g32870	aldehyde dehydrogenase, putative	1		-1,00	
LOC_Os08g35050	ARID/BRIGHT DNA-binding domain containing protein	1		-0.77	
LOC_Os08g35720	retrotransposon protein, putative, unclassified	1		-1.04	
LOC_Os08g35860	cytokinin dehydrogenase precursor, putative	1	-0.85		
LOC_Os08g37610	expressed protein	1		-1.12	
LOC_Os08g39100	protein phosphatase 2C, putative	1		-0.78	
LOC_Os08g41960	OsMADS37 - MADS-box family gene with MIKC* type-box	1		-0.87	
LOC_Os08g42198	auxin responsive protein, putative	1		-0.83	-0.75
LOC_Os08g42420	expressed protein	1		-1.1	
LOC_Os08g42430	membrane associated DUF588 domain containing protein, putative	1	-0.82		
LOC_Os08g42440	CCT/B-box zinc finger protein, putative	1		-1,00	
LOC_Os08g44270	vignain precursor, putative	1		-0.98	
LOC_Os08g44340	monodehydroascorbate reductase, putative	1		-0.74	
LOC_Os09g04300	expressed protein	1		-0.81	
LOC_Os09g07440	retrotransposon protein, putative, unclassified	1		-1.14	
LOC_Os09g12240	serine/threonine-protein kinase BRI1-like 1 precursor, putative	1		-1.19	
LOC_Os09g12790	potassium channel protein, putative	1		-0.7	
LOC_Os09g14600	peroxisomal membrane protein-related, putative	1		-0.71	
LOC_Os09g20990	trehalose-6-phosphate synthase, putative	1		-2.37	
LOC_Os09g24350	THION32 - Plant thionin family protein precursor	1		-0.78	
LOC_Os09g24370	expressed protein	1		-1.64	0.84
LOC_Os09g25000	spotted leaf 11, putative	1	-0.81		
LOC_Os09g26390	AT-rich interaction region, putative	1		-1.34	
LOC_Os09g32300	KH domain-containing protein, putative	1		-0.78	
LOC_Os09g33830	solute carrier family 35 member F1, putative	1		-1.79	
LOC_Os09g34860	hydrolase, alpha/beta fold family domain containing protein	1		-1.07	
LOC_Os09g37860	NOL1/NOP2/sun family protein, putative	1		-1.05	
LOC_Os09g38530	transmembrane 9 superfamily member, putative	1		-1.67	
LOC_Os09g39070	thiol protease SEN102 precursor, putative	1		-1.28	
LOC_Os09g39400	histidine-containing phosphotransfer protein, putative	1		-1.05	
LOC_Os10g01380	CW7, putative	1		-0.78	
LOC_Os10g08319	cytochrome P450, putative	1	1.11	-0.87	
LOC_Os10g10990	transcription initiation factor IIF, alpha subunit domain containing protein	1	-0.72	-1.47	
LOC_Os10g12760	retrotransposon protein, putative, unclassified	1		-0.74	
LOC_Os10g14814	ATFMN/FHY, putative	1		-0.79	
LOC_Os10g15390	ribosomal protein S7, putative	1		-0.93	

LOC_Os10g22730	expressed protein	1	-0.8	-0.73	
LOC_Os10g25130	aminotransferase, classes I and II, domain containing protein	1	-0.88	-1.79	
LOC_Os10g25180	phosphoinositide phosphatase family protein, putative	1		-0.71	
LOC_Os10g26850	hypothetical protein	1		-1.16	
LOC_Os10g30770	inorganic phosphate transporter, putative	1	-0.96		
LOC_Os10g31000	ubiquitin-conjugating enzyme, putative	1		-1.31	
LOC_Os10g31730	retrotransposon protein, putative, unclassified	1	-1.69	-1.53	-0.98
LOC_Os10g31970	SNF2 family N-terminal domain containing protein	1		-1.02	
LOC_Os10g34400	expressed protein	1		-1.08	
LOC_Os10g34660	expressed protein	1		-0.77	
LOC_Os10g35010	ATTIC110/TIC110, putative	1		-1.2	
LOC_Os10g35090	Rf1, mitochondrial precursor, putative	1		-1.17	
LOC_Os10g36370	heat shock protein DnaJ, putative	1		-1.02	
LOC_Os10g36680	ubiquitin carboxyl-terminal hydrolase, family 1, putative	1		-0.86	
LOC_Os10g37730	pollen ankyrin, putative	1		-1.18	
LOC_Os10g38090	cytochrome P450, putative	1		-0.84	
LOC_Os10g38276	chloroplast ATP synthase a chain precursor, putative	1		-1.65	-1.13
LOC_Os10g38314	glutathione S-transferase, N-terminal domain containing protein	1		-1.69	-1.12
LOC_Os10g38640	glutathione S-transferase, putative	1	-0.8		
LOC_Os10g39440	transporter family protein, putative	1		-0.85	
LOC_Os10g40430	LTPL139 - Protease inhibitor/seed storage/LTP family protein precursor	1		-0.72	
LOC_Os10g40480	LTPL143 - Protease inhibitor/seed storage/LTP family protein precursor	1		-0.84	
LOC_Os10g40520	LTPL145 - Protease inhibitor/seed storage/LTP family protein precursor	1		-0.82	
LOC_Os10g41510	calcineurin B, putative	1		-0.81	
LOC_Os10g42439	heat shock protein DnaJ, putative	1		-0.86	
LOC_Os10g42710	RCD1, putative	1		-1.92	
LOC_Os11g02190	expressed protein	1		-0.76	
LOC_Os11g03900	glucosamine--fructose-6-phosphate aminotransferase 1, putative	1		-1.03	
LOC_Os11g04010	ICE-like protease p20 domain containing protein, putative	1		-0.92	
LOC_Os11g04409	expressed protein	1	-0.85		
LOC_Os11g04950	splicing factor, putative	1		-0.76	
LOC_Os11g05400	Ser/Thr protein phosphatase family protein, putative	1		-0.95	
LOC_Os11g06750	ribosomal protein L3, putative	1	-0.86	-1.73	
LOC_Os11g06890	vacuolar ATP synthase, putative	1		-1.21	
LOC_Os11g08180	aspartic proteinase Asp1 precursor, putative	1		-0.82	
LOC_Os11g09329	VHS and GAT domain containing protein	1		-0.97	
LOC_Os11g10480	dehydrogenase, putative	1		-1.17	
LOC_Os11g11880	retrotransposon protein, putative, LINE subclass	1		-0.84	
LOC_Os11g12240	retrotransposon protein, putative, unclassified	1		-1.13	
LOC_Os11g12300	NBS-LRR disease resistance protein, putative	1		-0.82	
LOC_Os11g14220	tubulin/FtsZ domain containing protein, putative	1	-0.87		
LOC_Os11g15040	S-adenosyl-L-methionine:benzoic acid/salicylic acid carboxyl methyltransferase, putative	1	-0.72		
LOC_Os11g15340	SAM dependent carboxyl methyltransferase family protein, putative	1		-0.84	
LOC_Os11g16960	carnitine racemase like protein, putative	1		-0.71	
LOC_Os11g18880	UBX domain-containing protein, putative	1		-0.84	
LOC_Os11g32110	auxin response factor, putative	1		-0.89	
LOC_Os11g32930	hypothetical protein	1	-0.95	-0.93	-0.73
LOC_Os11g34190	expressed protein	1		-0.91	
LOC_Os11g38040	expressed protein	1	-0.81		
LOC_Os11g39640	zinc finger, C3HC4 type domain containing protein	1	-0.74		
LOC_Os11g47930	alpha-hemolysin, putative	1		-1,00	
LOC_Os12g01744	expressed protein	1		-1.32	
LOC_Os12g04204	expressed protein	1		-1.29	
LOC_Os12g06490	STE_PAK_Ste20_Slob_Wnk.6 - STE kinases include homologs to sterile 7, sterile 11	1	-0.99	-1.18	-1.2

	and sterile 20 from yeast				
LOC_Os12g07180	transposon protein, putative, Pong sub-class	1		-0.73	
LOC_Os12g13290	expressed protein	1		-1.2	
LOC_Os12g15420	nucampholin, putative	1		-0.97	
LOC_Os12g16220	nmrA-like family domain containing protein	1		-0.86	
LOC_Os12g25710	bifunctional aminoacyl-tRNA synthetase, putative	1		-1.76	
LOC_Os12g30540	ubiquitin carboxyl-terminal hydrolase, putative	1		-0.7	
LOC_Os12g32130	trehalose phosphatase, putative	1		-1.02	
LOC_Os12g35570	tRNA synthetase, putative	1	-0.77	-1.4	
LOC_Os12g35910	conserved hypothetical protein	1		-0.75	
LOC_Os12g37570	protein kinase family protein, putative	1		-0.89	
LOC_Os12g38051	metallothionein, putative	1		-1.45	
LOC_Os12g38051	metallothionein, putative	1		-1.15	
LOC_Os12g38300	metallothionein, putative	1		-0.98	
LOC_Os12g39420	nucleobase-ascorbate transporter, putative	1		-1.42	
LOC_Os12g40279	protein kinase domain containing protein	1	-1.05		
LOC_Os12g40490	LIM domain-containing protein, putative	1		-1.25	
LOC_Os12g41840	protein transport protein Sec61 subunit alpha, putative	1	-0.95	-0.91	-0.72
LOC_Os12g42200	ATCHX, putative	1		-0.72	
LOC_Os01g01450	stress responsive protein, putative	2	0.71		
LOC_Os01g01660	isoflavone reductase, putative	2	1.17		
LOC_Os01g01710	1-deoxy-D-xylulose 5-phosphate reductoisomerase, chloroplast precursor, putative	2	0.85		
LOC_Os01g02010	expressed protein	2	0.9		0.99
LOC_Os01g03630	multicopper oxidase domain containing protein	2			2.47
LOC_Os01g03640	multicopper oxidase domain containing protein	2	0.89		
LOC_Os01g03680	BBTI8 - Bowman-Birk type bran trypsin inhibitor precursor	2			3.53
LOC_Os01g04040	BBTI11 - Bowman-Birk type bran trypsin inhibitor precursor, putative	2	0.77		
LOC_Os01g04050	BBTI12 - Bowman-Birk type bran trypsin inhibitor precursor	2	0.77		0.86
LOC_Os01g04630	muconate cycloisomerase, putative	2			2.43
LOC_Os01g04640	reticulon domain containing protein, putative	2			1.89
LOC_Os01g05064	expressed protein	2			0.73
LOC_Os01g05140	expressed protein	2			0.75
LOC_Os01g05530	expressed protein	2			1.3
LOC_Os01g06220	gibberellin receptor GID1L2, putative	2	0.89	1.01	1.7
LOC_Os01g06280	TKL_IRAK_CrRLK1L-1.4 - The CrRLK1L-1 subfamily has homology to the CrRLK1L homolog	2	0.72		
LOC_Os01g06580	fasciclin domain containing protein	2	1.11		
LOC_Os01g06600	glutaryl-CoA dehydrogenase, mitochondrial precursor, putative	2			2.58
LOC_Os01g07370	KIP1, putative	2			1.8
LOC_Os01g09620	zinc finger/CCCH transcription factor, putative	2			0.76
LOC_Os01g11010	peptide-N4-asparagine amidase A, putative	2	1.03		1.21
LOC_Os01g11160	amino acid permease family protein, putative	2	1.06		
LOC_Os01g11620	GDSL-like lipase/acylhydrolase, putative	2	1,00		
LOC_Os01g12070	endoglucanase precursor, putative	2	0.98		
LOC_Os01g12940	phosphorylase domain containing protein, putative	2	0.84		
LOC_Os01g13030	OsIAA3 - Auxin-responsive Aux/IAA gene family member	2			2.67
LOC_Os01g13610	isoflavone reductase homolog IRL, putative	2	0.9		1.53
LOC_Os01g14670	Cupin domain containing protein	2	0.84		
LOC_Os01g14990	GCRP6 - Glycine and cysteine rich family protein precursor, putative	2	1.21		1.08
LOC_Os01g16170	PQ loop repeat domain containing protein	2			1.13
LOC_Os01g16960	pyruvate kinase, putative	2			0.71
LOC_Os01g19290	integral membrane protein DUF6 containing protein	2	0.99		1.46
LOC_Os01g21135	retrotransposon protein, putative, unclassified	2			1.7
LOC_Os01g21250	late embryogenesis abundant protein, putative	2	0.76		
LOC_Os01g22336	peroxidase precursor, putative	2			1.13

LOC_Os01g24710	jacalin-like lectin domain containing protein	2			0.84
LOC_Os01g25100	glutathione S-transferase, putative	2			0.79
LOC_Os01g27230	12-oxophytodienoate reductase, putative	2	0.81		0.97
LOC_Os01g32080	thiamine pyrophosphate enzyme, C-terminal TPP binding domain containing protein	2			0.79
LOC_Os01g32670	expressed protein	2			1.43
LOC_Os01g36240	peroxidase precursor, putative	2		0.76	
LOC_Os01g37590	peptide transporter PTR2, putative	2			1.07
LOC_Os01g37630	esterase, putative	2			0.95
LOC_Os01g38229	peptidyl-prolyl isomerase, putative	2	0.77		
LOC_Os01g38359	peptidyl-prolyl cis-trans isomerase, FKBP-type, putative	2	1.18		
LOC_Os01g39060	expressed protein	2			0.73
LOC_Os01g39330	helix-loop-helix DNA-binding domain containing protein	2	0.82		
LOC_Os01g39790	esterase/lipase/thioesterase, putative	2		0.88	
LOC_Os01g40290	expressed protein	2	0.74		0.77
LOC_Os01g41190	glycine-rich protein, putative	2	1.17		0.79
LOC_Os01g41430	UDP-glucoronosyl and UDP-glucosyl transferase, putative	2			1,00
LOC_Os01g42200	expressed protein	2			1.19
LOC_Os01g42380	pleiotropic drug resistance protein, putative	2	0.7		0.71
LOC_Os01g43580	kinesin motor domain containing protein, putative	2	0.77		
LOC_Os01g43710	cytochrome P450 72A1, putative	2		0.84	1.46
LOC_Os01g43890	OsSCP4 - Putative Serine Carboxypeptidase homologue	2			0.79
LOC_Os01g44960	hydrolase, alpha/beta fold family domain containing protein	2	0.71		
LOC_Os01g45110	anthocyanin 3-O-beta-glucosyltransferase, putative	2		0.74	
LOC_Os01g45250	DUF1645 domain containing protein, putative	2			0.88
LOC_Os01g45470	expressed protein	2	0.92		
LOC_Os01g45730	zinc finger C-x8-C-x5-C-x3-H type family protein	2			0.76
LOC_Os01g45900	OsFBX20 - F-box domain containing protein	2			0.85
LOC_Os01g45990	potassium channel AKT1, putative	2	0.78		
LOC_Os01g47780	fasciclin domain containing protein	2			0.76
LOC_Os01g48130	no apical meristem protein, putative	2			0.79
LOC_Os01g48360	expressed protein	2			0.92
LOC_Os01g48390	expressed protein	2			0.71
LOC_Os01g49320	glycosyl hydrolase, putative	2	0.77		
LOC_Os01g49710	glutathione S-transferase, putative	2		1.43	1.76
LOC_Os01g49720	glutathione S-transferase, putative	2	1.03	2,00	2.2
LOC_Os01g50100	ABC transporter, ATP-binding protein, putative	2	0.84	0.76	1.07
LOC_Os01g50170	eukaryotic aspartyl protease domain containing protein	2	0.94		
LOC_Os01g51570	glycosyl hydrolases family 17, putative	2	1.56		
LOC_Os01g51870	methyltransferase, putative	2			0.97
LOC_Os01g53350	anthocyanidin 5,3-O-glucosyltransferase, putative	2	0.7	0.76	1.3
LOC_Os01g54670	coiled-coil domain-containing protein 25, putative	2			1.07
LOC_Os01g55720	expressed protein	2			1.11
LOC_Os01g57250	expressed protein	2			7.01
LOC_Os01g57880	plastocyanin-like domain containing protein, putative	2			0.83
LOC_Os01g58130	expressed protein	2			0.78
LOC_Os01g58280	OsSub8 - Putative Subtilisin homologue	2	0.9	0.72	1.35
LOC_Os01g58290	OsSub9 - Putative Subtilisin homologue	2	1,00		
LOC_Os01g58320	expressed protein	2			0.88
LOC_Os01g58690	endonuclease/exonuclease/phosphatase family domain containing protein	2			0.8
LOC_Os01g58960	cytochrome P450, putative	2		0.83	
LOC_Os01g59160	UBA/TS-N domain containing protein	2			0.78
LOC_Os01g59180	OsFBX27 - F-box domain containing protein	2	0.73		
LOC_Os01g60020	NAC domain transcription factor, putative	2		0.72	
LOC_Os01g60730	RING-H2 finger protein, putative	2			0.89

LOC_Os01g61940	white-brown complex homolog protein, putative	2	1,00		0.83
LOC_Os01g62360	expressed protein	2			0.72
LOC_Os01g62420	triosephosphate isomerase, cytosolic, putative	2		0.92	0.96
LOC_Os01g62610	peptidyl-prolyl cis-trans isomerase, FKBP-type, putative	2			1.11
LOC_Os01g62630	aspartic proteinase nepenthesin precursor, putative	2	0.9		
LOC_Os01g62870	oxidoreductase, aldo/keto reductase family protein, putative	2			0.91
LOC_Os01g63210	SOUL heme-binding protein, putative	2	1.05	1.7	2.16
LOC_Os01g63270	alpha-glucan phosphorylast isozyme, putative	2			0.72
LOC_Os01g63540	cytochrome P450, putative	2			1.82
LOC_Os01g64000	bZIP transcription factor, putative	2	0.73		
LOC_Os01g64100	glycosyl hydrolase, putative	2			1.64
LOC_Os01g65440	universal stress protein domain containing protein, putative	2			1.09
LOC_Os01g66030	OsMADS2 - MADS-box family gene with MIKCc type-box	2			1.1
LOC_Os01g67090	IQ calmodulin-binding motif domain containing protein	2	1.11	0.72	0.91
LOC_Os01g68720	keratin, type I cytoskeletal 9, putative	2			0.71
LOC_Os01g70190	exostosin family domain containing protein	2	0.75		
LOC_Os01g70850	esterase, putative	2	1.46	2.56	2.97
LOC_Os01g70860	esterase, putative	2		0.71	
LOC_Os01g71340	glycosyl hydrolases family 17, putative	2	1.63		
LOC_Os01g71650	glycosyl hydrolases family 17, putative	2	1.44		
LOC_Os01g71710	amino acid permease family protein, putative	2	0.72		0.77
LOC_Os01g71740	amino acid permease family protein, putative	2	0.71		
LOC_Os01g72140	glutathione S-transferase, putative	2			0.71
LOC_Os01g72150	glutathione S-transferase, putative	2		0.91	1.6
LOC_Os01g74152	haloacid dehalogenase-like hydrolase family protein, putative	2			0.74
LOC_Os01g74300	metallothionein, putative	2	1.53		0.98
LOC_Os02g02340	glycerol-3-phosphate acyltransferase, putative	2	0.81		
LOC_Os02g02400	catalase isozyme A, putative	2	1.04		
LOC_Os02g03430	expressed protein	2			1.65
LOC_Os02g05980	phytosulfokine receptor precursor, putative	2			1.03
LOC_Os02g07340	HEAT repeat family protein, putative	2			1.26
LOC_Os02g08330	gp176, putative	2			1.03
LOC_Os02g08490	chaperone protein clpB 1, putative	2			0.97
LOC_Os02g12080	expressed protein	2			0.77
LOC_Os02g13400	expressed protein	2			2.41
LOC_Os02g13980	hypothetical protein	2	0.72		
LOC_Os02g15540	expressed protein	2	0.83		0.73
LOC_Os02g15700	expressed protein	2			0.82
LOC_Os02g16909	dynein light chain type 1 domain containing protein	2			1.13
LOC_Os02g17620	isochorismatase family protein, putative	2	0.85		0.78
LOC_Os02g19640	IQ calmodulin-binding motif family protein, putative	2	0.79		
LOC_Os02g20310	hypothetical protein	2			1.15
LOC_Os02g21300	transposon protein, putative, unclassified	2	0.82	1.7	1.87
LOC_Os02g25960	ankyrin repeat family protein, putative	2	0.74		
LOC_Os02g28170	transferase family protein, putative	2			0.78
LOC_Os02g30100	cytochrome P450, putative	2			0.8
LOC_Os02g30240	expressed protein	2	0.71		
LOC_Os02g31840	expressed protein	2	0.73		
LOC_Os02g32970	hydrolase, alpha/beta fold family protein, putative	2	0.84		
LOC_Os02g32980	Cupin domain containing protein	2	0.78		
LOC_Os02g33070	expressed protein	2			1.05
LOC_Os02g33390	pectinesterase inhibitor domain containing protein, putative	2	0.73		
LOC_Os02g33820	abscisic stress-ripening, putative	2			0.7
LOC_Os02g35329	RING-H2 finger protein ATL3F, putative	2	0.73	0.86	1.47

LOC_Os02g35347	RING-H2 finger protein ATL3F, putative	2		0.71	1.32
LOC_Os02g35365	RING-H2 finger protein ATL3F, putative	2			1.12
LOC_Os02g35440	RING-H2 finger protein ATL4O precursor, putative	2			1.33
LOC_Os02g36110	cytochrome P450, putative	2	1.25	0.87	1.39
LOC_Os02g36140	terpene synthase, putative	2	1.61	1.06	1,00
LOC_Os02g36190	cytochrome P450, putative	2	1.39	1,00	1.01
LOC_Os02g36210	ent-kaurene synthase, chloroplast precursor, putative	2	1.04	0.74	1.07
LOC_Os02g36280	cytochrome P450, putative	2			0.82
LOC_Os02g36530	hypothetical protein	2			1.05
LOC_Os02g36830	cytokinin-O-glucosyltransferase 2, putative	2	0.76		0.8
LOC_Os02g37000	mitochondrial prohibitin complex protein 1, putative	2		0.78	0.85
LOC_Os02g37260	hypothetical protein	2			1.88
LOC_Os02g37320	heavy metal associated domain containing protein	2	0.8		
LOC_Os02g38120	BTBN3 - Bric-a-Brac, Tramtrack, Broad Complex BTB domain with non-phototropic hypocotyl 3 NPH3 domain	2	0.74		
LOC_Os02g39570	ACT domain containing protein	2	0.86		
LOC_Os02g41780	transporter-related, putative	2	0.75		
LOC_Os02g43660	plastocyanin-like domain containing protein, putative	2	0.97		0.8
LOC_Os02g44108	expansin precursor, putative	2	0.79		
LOC_Os02g44310	LTPL112 - Protease inhibitor/seed storage/LTP family protein precursor	2			0.93
LOC_Os02g44654	cytochrome P450, putative	2	0.73		
LOC_Os02g44990	OsFBDUF13 - F-box and DUF domain containing protein	2			0.89
LOC_Os02g47780	hydrolase, alpha/beta fold family domain containing protein	2	0.97		
LOC_Os02g47790	monodehydroascorbate reductase, putative	2			0.76
LOC_Os02g48450	xylem cysteine proteinase 2 precursor, putative	2			1.11
LOC_Os02g48710	expressed protein	2	1.47	0.74	
LOC_Os02g48730	rho GDP-dissociation inhibitor 1, putative	2			0.72
LOC_Os02g48870	aspartic proteinase nepenthesin-2 precursor, putative	2	0.71		
LOC_Os02g49140	glycosyltransferase, putative	2	0.83		0.73
LOC_Os02g49520	armadillo/beta-catenin repeat family protein, putative	2	0.85		
LOC_Os02g49850	plastocyanin-like domain containing protein, putative	2			0.73
LOC_Os02g50000	GDSL-like lipase/acylhydrolase, putative	2			1.25
LOC_Os02g52150	heat shock 22 kDa protein, mitochondrial precursor, putative	2		1.16	1.94
LOC_Os02g53130	nitrate reductase, putative	2		0.88	
LOC_Os02g55600	expressed protein	2			1.11
LOC_Os03g01300	LTPL114 - Protease inhibitor/seed storage/LTP family protein precursor	2	1.1		
LOC_Os03g01300	LTPL114 - Protease inhibitor/seed storage/LTP family protein precursor	2	1.29		
LOC_Os03g01320	LTPL116 - Protease inhibitor/seed storage/LTP family protein precursor	2	0.79		1.63
LOC_Os03g01940	expressed protein	2			0.91
LOC_Os03g02550	OsFBX76 - F-box domain containing protein	2			0.71
LOC_Os03g03320	expressed protein	2	0.75		
LOC_Os03g04060	CHIT16 - Chitinase family protein precursor	2	0.81		
LOC_Os03g04240	glutathione S-transferase, putative	2	0.85		
LOC_Os03g05700	expressed protein	2	1.97	2.82	3.15
LOC_Os03g06170	hsp20/alpha crystallin family protein, putative	2			1.49
LOC_Os03g06350	DUF623 domain containing protein	2			1.06
LOC_Os03g07270	glycine-rich cell wall protein, putative	2			2.49
LOC_Os03g07590	hypothetical protein	2			1.03
LOC_Os03g08170	protein kinase APK1B, chloroplast precursor, putative	2			0.97
LOC_Os03g08580	expressed protein	2			0.78
LOC_Os03g08600	glycosyl transferase, putative	2			0.72
LOC_Os03g09020	dehydrogenase, putative	2	1.23		
LOC_Os03g09220	stage II sporulation protein E, putative	2			0.74
LOC_Os03g09880	AIR12, putative	2	0.85		0.94
LOC_Os03g10050	serine acetyltransferase protein, putative	2			1.04

LOC_Os03g12030	3-ketoacyl-CoA synthase, putative	2	0.75		
LOC_Os03g13030	lecithin cholesterol acyltransferase, putative	2			0.83
LOC_Os03g13050	E2F-related protein, putative	2			0.73
LOC_Os03g13140	non-symbiotic hemoglobin 2, putative	2	0.8		0.92
LOC_Os03g13150	non-symbiotic hemoglobin 2, putative	2			0.82
LOC_Os03g14150	expressed protein	2			1.08
LOC_Os03g14170	3-ketoacyl-CoA synthase, putative	2	0.85		
LOC_Os03g15220	DUF292 domain containing protein	2			1.08
LOC_Os03g15340	plastocyanin-like domain containing protein, putative	2	1.59		
LOC_Os03g15460	expressed protein	2			1.1
LOC_Os03g16960	cysteine-rich repeat secretory protein 55 precursor, putative	2		0.83	
LOC_Os03g17350	white-brown complex homolog protein, putative	2	1.04		0.96
LOC_Os03g17470	IN2-1 protein, putative	2	0.91		1.04
LOC_Os03g18110	proteins of unknown function domain containing protein, putative	2			1.1
LOC_Os03g18980	kinesin motor domain containing protein	2	0.89		0.91
LOC_Os03g20120	glycosyl transferase 8 domain containing protein, putative	2	0.81		
LOC_Os03g20330	VQ domain containing protein, putative	2			0.73
LOC_Os03g22020	peroxidase precursor, putative	2	0.71		0.76
LOC_Os03g31480	expansin precursor, putative	2			1.02
LOC_Os03g32330	expressed protein	2			0.83
LOC_Os03g37290	cytochrome P450, putative	2	1.39	1.34	1.21
LOC_Os03g38790	expressed protein	2			0.76
LOC_Os03g39850	glutathione S-transferase, putative	2			0.99
LOC_Os03g40320	conserved hypothetical protein	2	1.34		
LOC_Os03g42130	gibberellin 20 oxidase 2, putative	2		0.8	
LOC_Os03g42600	expressed protein	2	0.85		0.72
LOC_Os03g42630	No apical meristem protein, putative	2			0.96
LOC_Os03g44260	conserved hypothetical protein	2			0.8
LOC_Os03g44320	hypothetical protein	2	0.83		
LOC_Os03g46060	thaumatin family domain containing protein	2			1.17
LOC_Os03g46070	thaumatin, putative	2	1.2		
LOC_Os03g46150	LTPL72 - Protease inhibitor/seed storage/LTP family protein precursor	2			0.73
LOC_Os03g46200	acetyltransferase, GNAT family, putative	2	0.79		0.83
LOC_Os03g50130	microsomal glutathione S-transferase 3, putative	2		0.76	1.27
LOC_Os03g50790	conserved hypothetical protein	2		0.87	0.85
LOC_Os03g51600	tubulin/FtsZ domain containing protein, putative	2			0.81
LOC_Os03g52360	PIII3 - Proteinase inhibitor II family protein precursor, putative	2			2.27
LOC_Os03g52370	PIII4 - Proteinase inhibitor II family protein precursor	2		0.73	1.64
LOC_Os03g52380	PIII5 - Proteinase inhibitor II family protein precursor	2			0.71
LOC_Os03g52390	PIII1 - Proteinase inhibitor II family protein precursor	2	0.71	0.75	2.15
LOC_Os03g52860	lipoxygenase, putative	2	1.38		
LOC_Os03g53360	transferase family protein, putative	2	0.9		
LOC_Os03g53800	periplasmic beta-glucosidase precursor, putative	2			0.73
LOC_Os03g54050	anther-specific proline-rich protein APG precursor, putative	2			0.75
LOC_Os03g55050	UDP-glucoronosyl and UDP-glucosyl transferase domain containing protein	2			1.41
LOC_Os03g55240	cytochrome P450, putative	2		1.51	2.38
LOC_Os03g56540	heat shock protein DnaJ, putative	2	0.74	1.39	1.48
LOC_Os03g57200	glutathione S-transferase, putative	2	1.17		1.51
LOC_Os03g57640	gibberellin receptor GID1L2, putative	2			0.8
LOC_Os03g57970	LTPL73 - Protease inhibitor/seed storage/LTP family protein precursor	2	1.09		0.77
LOC_Os03g57980	LTPL99 - Protease inhibitor/seed storage/LTP family protein precursor	2	0.99		0.72
LOC_Os03g57990	LTPL74 - Protease inhibitor/seed storage/LTP family protein precursor	2	0.88		
LOC_Os03g59210	hypothetical protein	2	0.71		
LOC_Os03g61160	expressed protein	2			0.77

LOC_Os03g62480	anthocyanidin 5,3-O-glucosyltransferase, putative	2	1.51	1.79	2.57
LOC_Os03g62490	prohibitin-2, putative	2		0.71	0.8
LOC_Os03g63370	CRAL/TRIO domain containing protein	2	0.94		
LOC_Os03g64300	THION30 - Plant thionin family protein precursor	2			0.78
LOC_Os04g01140	cytochrome P450 93A2, putative	2	0.74		
LOC_Os04g02530	expressed protein	2			1.34
LOC_Os04g03164	expressed protein	2	0.98		1,00
LOC_Os04g07280	AGAP002737-PA, putative	2	1.23		
LOC_Os04g07890	AGAP002737-PA, putative	2			1.04
LOC_Os04g08370	Leucine Rich Repeat family protein	2			1.02
LOC_Os04g08550	oxidoreductase, aldo/keto reductase family protein, putative	2	0.75		1.69
LOC_Os04g10000	sex determination protein tasselseed-2, putative	2	0.95	0.89	
LOC_Os04g10010	sex determination protein tasselseed-2, putative	2	1.25	0.99	0.81
LOC_Os04g10060	ent-kaurene synthase, chloroplast precursor, putative	2	0.94		0.72
LOC_Os04g10160	cytochrome P450, putative	2	1.25	0.76	0.92
LOC_Os04g11400	expressed protein	2			0.82
LOC_Os04g12669	conserved hypothetical protein	2			0.87
LOC_Os04g12720	indole-3-acetate beta-glucosyltransferase, putative	2			1.21
LOC_Os04g12960	UDP-glucoronosyl/UDP-glucosyl transferase, putative	2		0.8	1.13
LOC_Os04g15690	DSBA-like thioredoxin domain containing protein	2	1.08		0.72
LOC_Os04g15920	dehydrogenase, putative	2	0.96		
LOC_Os04g16450	aquaporin protein, putative	2	0.73		
LOC_Os04g16970	zinc finger, C3HC4 type domain containing protein	2	0.76		
LOC_Os04g17650	sucrose synthase, putative	2			0.99
LOC_Os04g17660	rhodanese-like domain containing protein, putative	2	0.87		0.77
LOC_Os04g22080	retrotransposon protein, putative, unclassified	2	1.11		0.84
LOC_Os04g27060	oxidoreductase, aldo/keto reductase family protein, putative	2	2.06	2.94	3.61
LOC_Os04g28250	cysteine proteinase inhibitor precursor, putative	2			0.91
LOC_Os04g29030	dehydrogenase, putative	2			1.33
LOC_Os04g31520	RALFL21 - Rapid ALkalinization Factor RALF family protein precursor	2	0.81		0.82
LOC_Os04g33570	RCN4 Centroradialis-like1 homologous to TFL1 gene; contains Pfam profile PF01161: Phosphatidylethanolamine-binding protein	2	0.76		
LOC_Os04g37680	alpha/beta hydrolase fold, putative	2	1.11		0.95
LOC_Os04g38530	aldose 1-epimerase, putative	2		0.71	1.4
LOC_Os04g39350	heavy metal associated domain containing protein	2	1.27		
LOC_Os04g40460	cytochrome P450, putative	2			0.81
LOC_Os04g40730	oxidoreductase, short chain dehydrogenase/reductase family, putative	2	0.88		
LOC_Os04g40900	exonuclease, putative	2			0.98
LOC_Os04g41220	transposon protein, putative, unclassified	2			0.76
LOC_Os04g43610	transposon protein, putative, CACTA, En/Spm sub-class	2			0.94
LOC_Os04g45970	glutamate dehydrogenase protein, putative	2			0.73
LOC_Os04g46560	lactate/malate dehydrogenase, putative	2			1.4
LOC_Os04g47300	CAMK_CAMK_like.26 - CAMK includes calcium/calmodulin depedent protein kinases	2			1.01
LOC_Os04g50700	pathogenesis-related Bet v I family protein, putative	2	0.95		1.18
LOC_Os04g50970	seed specific protein Bn15D1B, putative	2	0.76		0.87
LOC_Os04g51150	transposon protein, putative, unclassified	2	0.98	1.58	1.56
LOC_Os04g51160	transposon protein, putative, unclassified	2	1.07	1.05	1.22
LOC_Os04g51980	transferase family domain containing protein	2			0.84
LOC_Os04g51990	transferase family domain containing protein	2	0.8		0.88
LOC_Os04g52590	protein kinase domain containing protein	2			1.25
LOC_Os04g52720	Cupin domain containing protein	2		0.71	
LOC_Os04g52780	leucine-rich repeat receptor protein kinase EXS precursor, putative	2	0.77		0.74
LOC_Os04g52850	OTU-like cysteine protease family protein, putative	2			0.81
LOC_Os04g52880	expressed protein	2	0.75		1.18

LOC_Os04g52900	ABC transporter family protein, putative	2			0.92
LOC_Os04g53520	expressed protein	2			0.72
LOC_Os04g53990	ethylene-responsive protein related, putative	2			1.4
LOC_Os04g54590	expressed protein	2			0.89
LOC_Os04g55150	UBA/TS-N domain containing protein	2			0.75
LOC_Os04g56420	retrotransposon protein, putative, unclassified	2			0.98
LOC_Os04g56470	amino acid transporter, putative	2			0.88
LOC_Os04g57430	uncharacterized protein At4g06744 precursor, putative	2			1,00
LOC_Os04g57760	expressed protein	2			0.78
LOC_Os04g57860	endoglucanase precursor, putative	2	0.72		0.72
LOC_Os04g58090	harpin-induced protein 1 domain containing protein	2	0.87		0.8
LOC_Os05g01256	helix-loop-helix DNA-binding domain containing protein	2			0.76
LOC_Os05g02070	expressed protein	2	0.85		0.77
LOC_Os05g02250	expressed protein	2	0.78		
LOC_Os05g02890	white-brown complex homolog protein 16, putative	2			0.74
LOC_Os05g02900	expressed protein	2	0.73		0.79
LOC_Os05g04410	peroxidase precursor, putative	2			0.82
LOC_Os05g04450	peroxidase precursor, putative	2			0.88
LOC_Os05g04500	peroxidase precursor, putative	2	1.27		
LOC_Os05g05080	expressed protein	2			0.85
LOC_Os05g05680	1-aminocyclopropane-1-carboxylate oxidase, putative	2		0.95	
LOC_Os05g06720	GDSL-like lipase/acylhydrolase, putative	2	0.96		0.81
LOC_Os05g06970	peroxidase precursor, putative	2			0.74
LOC_Os05g10330	HAD superfamily phosphatase, putative	2	0.72		
LOC_Os05g10370	acid phosphatase, putative	2	1.05		
LOC_Os05g12630	expressed protein	2	1.68		1.2
LOC_Os05g13900	retrotransposon protein, putative, unclassified	2			0.85
LOC_Os05g15630	membrane associated DUF588 domain containing protein, putative	2	0.99		1.06
LOC_Os05g15690	expansin precursor, putative	2	0.82		
LOC_Os05g15770	glycosyl hydrolase, putative	2			1.11
LOC_Os05g18540	transposon protein, putative, CACTA, En/Spm sub-class	2			0.78
LOC_Os05g19150	hydrolase, alpha/beta fold family domain containing protein	2			0.73
LOC_Os05g25430	receptor-like protein kinase At3g46290 precursor, putative	2			0.87
LOC_Os05g27490	retrotransposon protein, putative, unclassified	2			0.85
LOC_Os05g30740	C2 domain containing protein, putative	2			0.71
LOC_Os05g31140	glycosyl hydrolases family 17, putative	2			1.23
LOC_Os05g31910	white-brown complex homolog protein 16, putative	2			0.89
LOC_Os05g32860	expressed protein	2			0.82
LOC_Os05g33130	CHIT17 - Chitinase family protein precursor	2	0.73		0.99
LOC_Os05g33140	CHIT5 - Chitinase family protein precursor	2	1.41		0.73
LOC_Os05g33380	fructose-bisphospate aldolase isozyme, putative	2		0.7	
LOC_Os05g38350	glycerol-3-phosphate acyltransferase 8, putative	2	0.77		0.79
LOC_Os05g38390	laccase precursor protein, putative	2			0.76
LOC_Os05g39350	PMR5, putative	2	0.73		
LOC_Os05g41180	peptidase, T1 family, putative	2		0.82	1.14
LOC_Os05g43940	O-methyltransferase, putative	2	2.24		1.09
LOC_Os05g45100	anthocyanidin 5,3-O-glucosyltransferase, putative	2	0.75	1.37	1.54
LOC_Os05g45110	anthocyanidin 5,3-O-glucosyltransferase, putative	2			1.22
LOC_Os05g45180	anthocyanidin 5,3-O-glucosyltransferase, putative	2			1.54
LOC_Os05g45450	DUF584 domain containing protein, putative	2			0.99
LOC_Os05g45950	outer mitochondrial membrane porin, putative	2			0.7
LOC_Os05g48270	auxin-responsive protein, putative	2	0.82		
LOC_Os05g48610	expressed protein	2			0.83
LOC_Os05g49370	DUF1645 domain containing protein, putative	2			0.74

LOC_Os05g50090	oxidoreductase, 2OG-FeII oxygenase domain containing protein, putative	2			2.82
LOC_Os05g50100	expressed protein	2	0.87		
LOC_Os05g51190	protein kinase family protein, putative	2			0.7
LOC_Os06g04169	hydrolase, alpha/beta fold family domain containing protein	2			0.8
LOC_Os06g04510	enolase, putative	2	1.18	2.21	2.58
LOC_Os06g05410	hypothetical protein	2		0.92	1.02
LOC_Os06g06210	expressed protein	2			0.83
LOC_Os06g06550	plant protein of unknown function domain containing protein	2			1.09
LOC_Os06g07932	flavonol synthase/flavanone 3-hydroxylase, putative	2	0.84		0.77
LOC_Os06g08032	flavonol synthase/flavanone 3-hydroxylase, putative	2	0.8		0.8
LOC_Os06g08640	transferase family protein, putative	2	1.68	0.71	
LOC_Os06g09900	expressed protein	2			0.79
LOC_Os06g11330	OsMADS55 - MADS-box family gene with MIKCc type-box	2	0.96		
LOC_Os06g11610	heat shock 22 kDa protein, mitochondrial precursor, putative	2	0.85	1.43	1.8
LOC_Os06g11660	phosphate-induced protein 1 conserved region domain containing protein	2			0.99
LOC_Os06g13190	expressed protein	2	-0.72		0.85
LOC_Os06g13220	expressed protein	2		1.07	1.13
LOC_Os06g14630	GDSL-like lipase/acylhydrolase, putative	2			0.71
LOC_Os06g16350	peroxidase precursor, putative	2	0.93		
LOC_Os06g18140	UDP-glucoronosyl and UDP-glucosyl transferase domain containing protein	2		0.94	1.33
LOC_Os06g18960	embryogenesis transmembrane protein, putative	2	0.78		
LOC_Os06g19180	hypothetical protein	2			0.83
LOC_Os06g19730	HEAT repeat family protein, putative	2		0.74	0.73
LOC_Os06g19800	BURP domain containing protein	2	1.79		1.03
LOC_Os06g20040	aspartic proteinase nepenthesin-2 precursor, putative	2			0.87
LOC_Os06g23114	copper methylamine oxidase precursor, putative	2			0.98
LOC_Os06g24180	cytochrome P450 84A1, putative	2	1.02		0.78
LOC_Os06g24420	transposon protein, putative, CACTA, En/Spm sub-class	2			0.94
LOC_Os06g25010	glycosyl hydrolase, putative	2			0.75
LOC_Os06g30920	hypothetical protein	2	0.73		
LOC_Os06g30950	transporter-related, putative	2	0.8		0.84
LOC_Os06g35520	peroxidase precursor, putative	2			1.26
LOC_Os06g36810	expressed protein	2	0.71		
LOC_Os06g38660	expressed protein	2			1.34
LOC_Os06g38750	proline-rich family protein, putative	2			1.42
LOC_Os06g38960	expressed protein	2	0.88		
LOC_Os06g41070	cytochrome P450, putative	2			1.42
LOC_Os06g43180	cyclase family protein, putative	2			0.71
LOC_Os06g43620	haemolysin-III, putative	2	0.92		
LOC_Os06g45850	OTU-like cysteine protease family protein, putative	2			3.04
LOC_Os06g47130	C2 domain containing protein	2			0.76
LOC_Os06g48200	glycosyl hydrolases family 16, putative	2			0.98
LOC_Os06g49840	OsMADS16 - MADS-box family gene with MIKCc type-box	2	0.88		0.78
LOC_Os07g01370	peroxidase precursor, putative	2	1.1		0.93
LOC_Os07g03288	SCP-like extracellular protein	2		1.1	1.12
LOC_Os07g03377	SCP-like extracellular protein	2		0.86	1.29
LOC_Os07g03418	phototropin, putative	2		0.85	1.34
LOC_Os07g03418	phototropin, putative	2		0.85	1.34
LOC_Os07g03590	SCP-like extracellular protein	2		0.78	
LOC_Os07g03710	SCP-like extracellular protein	2	1.01		
LOC_Os07g03730	SCP-like extracellular protein	2	2.66		
LOC_Os07g03770	Homeobox domain containing protein	2	0.71		
LOC_Os07g04810	protein kinase domain containing protein	2			0.91
LOC_Os07g04930	retrotransposon protein, putative, unclassified	2	0.95		

LOC_Os07g04950	retrotransposon protein, putative, unclassified	2	1.54		1.24
LOC_Os07g04960	app1, putative	2	1.11		0.84
LOC_Os07g08430	indole-3-glycerol phosphate lyase, chloroplast precursor, putative	2			0.74
LOC_Os07g09970	LTPL84 - Protease inhibitor/seed storage/LTP family protein precursor	2	0.86		0.97
LOC_Os07g10440	expressed protein	2	1.21	0.72	0.86
LOC_Os07g11739	cytochrome P450, putative	2	0.72	0.82	1.05
LOC_Os07g13800	cytokinin-N-glucosyltransferase, putative	2	0.74		1.22
LOC_Os07g17270	retrotransposon protein, putative, unclassified	2			1.13
LOC_Os07g18120	aldehyde oxidase, putative	2	0.92		
LOC_Os07g18750	LTPL42 - Protease inhibitor/seed storage/LTP family protein precursor	2			0.72
LOC_Os07g19000	LTPL41 - Protease inhibitor/seed storage/LTP family protein precursor	2			2.53
LOC_Os07g23410	fatty acid desaturase, putative	2	0.71		1.5
LOC_Os07g23430	fatty acid desaturase, putative	2	1.13		
LOC_Os07g24190	CESA3 - cellulose synthase	2	0.71		1.21
LOC_Os07g26630	aquaporin protein, putative	2			2.79
LOC_Os07g29330	serine/threonine-protein kinase CTR1, putative	2			0.73
LOC_Os07g30980	uvrD/REP helicase family protein, putative	2			1.27
LOC_Os07g31884	MATE efflux family protein, putative	2	0.9		
LOC_Os07g31960	UDP-glucoronosyl and UDP-glucosyl transferase domain containing protein	2			0.88
LOC_Os07g32010	UDP-glucoronosyl and UDP-glucosyl transferase domain containing protein	2			0.8
LOC_Os07g32060	UDP-glucoronosyl/UDP-glucosyl transferase, putative	2	0.74		
LOC_Os07g32570	OsAPRL1 adenosine 5'-phosphosulfate reductase-like OsAPRL1	2		1.03	
LOC_Os07g33350	hsp20/alpha crystallin family protein, putative	2			1.12
LOC_Os07g34260	chalcone and stilbene synthases, putative	2	1.03		
LOC_Os07g34750	expressed protein	2			1.58
LOC_Os07g34850	aspartic proteinase nepenthesin, putative	2			2.29
LOC_Os07g35480	glucan endo-1,3-beta-glucosidase precursor, putative	2	1.18		
LOC_Os07g35660	TKL_IRAK_DUF26-lc.21 - DUF26 kinases have homology to DUF26 containing loci	2	0.83		
LOC_Os07g35680	TKL_IRAK_DUF26-lc22 - DUF26 kinases have homology to DUF26 containing loci	2	1.06		0.96
LOC_Os07g39350	transporter family protein, putative	2	1,00		0.96
LOC_Os07g40220	expressed protein	2			0.88
LOC_Os07g40850	retrotransposon protein, putative, unclassified	2	1.18		0.86
LOC_Os07g40860	retrotransposon protein, putative, unclassified	2	1.31		1,00
LOC_Os07g40870	igA FC receptor precursor, putative	2	1.19		1.1
LOC_Os07g40890	igA FC receptor precursor, putative	2	1.04		0.98
LOC_Os07g40910	expressed protein	2	0.99		1.24
LOC_Os07g41410	EGG APPARATUS-1, putative	2	0.76		
LOC_Os07g41460	sulfotransferase domain containing protein	2			1.13
LOC_Os07g43240	SKP1-like protein 1B, putative	2		0.74	0.84
LOC_Os07g43800	EF hand family protein, putative	2	0.72		
LOC_Os07g44070	pectinacetylesterase domain containing protein	2			0.86
LOC_Os07g44140	cytochrome P450 72A1, putative	2			1.01
LOC_Os07g44180	OsRCI2-10 - Hydrophobic protein LTI6A	2			0.79
LOC_Os07g44250	dirigent, putative	2	1.13		
LOC_Os07g44410	WD40-like Beta Propeller Repeat family protein	2		0.71	1.42
LOC_Os07g44450	dirigent, putative	2			0.84
LOC_Os07g44920	expressed protein	2	1.61		
LOC_Os07g46852	sex determination protein tasselseed-2, putative	2			0.76
LOC_Os07g47210	GDSL-like lipase/acylhydrolase, putative	2	1.17		0.8
LOC_Os07g48010	peroxidase precursor, putative	2	1.45		1.01
LOC_Os07g48020	peroxidase precursor, putative	2	1.22		1.26
LOC_Os07g48030	peroxidase precursor, putative	2	1.26		
LOC_Os07g48060	peroxidase precursor, putative	2			0.85
LOC_Os07g48790	SNF1-related protein kinase regulatory subunit beta-1, putative	2			0.82

LOC_Os08g02230	FAD-binding and arabino-lactone oxidase domains containing protein, putative	2			0.95
LOC_Os08g04460	NADPH-dependent FMN reductase domain containing protein	2	0.87		
LOC_Os08g04560	decarboxylase, putative	2	1.2	0.92	0.9
LOC_Os08g05520	myb-like DNA-binding domain containing protein, putative	2	0.73		
LOC_Os08g05770	expressed protein	2			0.79
LOC_Os08g05960	expressed protein	2	1.59	2.24	3.18
LOC_Os08g05980	expressed protein	2	1.49	2.11	2.69
LOC_Os08g06550	acyl CoA binding protein, putative	2	0.86		0.85
LOC_Os08g07730	transferase family protein, putative	2		0.88	1.41
LOC_Os08g07880	phosphopantothenate--cysteine ligase, putative	2			1.18
LOC_Os08g08920	Cupin domain containing protein	2			0.81
LOC_Os08g09010	Cupin domain containing protein	2	1.03	0.78	1.48
LOC_Os08g09060	Cupin domain containing protein	2			1.19
LOC_Os08g09830	BTB and MATH domain containing protein, putative	2			1.21
LOC_Os08g10310	SHR5-receptor-like kinase, putative	2	0.73		
LOC_Os08g13890	exonuclease, putative	2			0.77
LOC_Os08g16810	retrotransposon protein, putative, SINE subclass	2	0.77		
LOC_Os08g16880	sas10/Utp3 family protein	2			0.85
LOC_Os08g20130	flavonol sulfotransferase, putative	2	0.98		
LOC_Os08g23140	expressed protein	2			0.74
LOC_Os08g23150	indole-3-glycerol phosphate synthase, chloroplast precursor, putative	2	0.78		0.91
LOC_Os08g24300	hypothetical protein	2			1.1
LOC_Os08g24750	xyloglucan fucosyltransferase, putative	2			0.9
LOC_Os08g24790	AIR12, putative	2	1.55		
LOC_Os08g26850	plant protein of unknown function domain containing protein	2			1.19
LOC_Os08g27240	ARID/BRIGHT DNA-binding domain containing protein	2			0.89
LOC_Os08g27840	phosphoenolpyruvate carboxylase, putative	2			0.98
LOC_Os08g28790	dirigent, putative	2			0.85
LOC_Os08g28880	patatin, putative	2	0.72		1.04
LOC_Os08g31340	heavy metal-associated domain containing protein	2	0.74		0.89
LOC_Os08g31850	expressed protein	2			0.85
LOC_Os08g31880	conserved hypothetical protein	2	0.87		
LOC_Os08g34984	DUF581 domain containing protein	2			0.72
LOC_Os08g37180	patatin, putative	2	1.29		0.75
LOC_Os08g37210	patatin, putative	2			1.07
LOC_Os08g37250	patatin, putative	2	1.08		1.46
LOC_Os08g37840	phosphate-induced protein 1 conserved region domain containing protein	2			0.75
LOC_Os08g38270	fasciclin domain containing protein	2			0.7
LOC_Os08g38910	caffeoyl-CoA O-methyltransferase, putative	2	1.35		0.72
LOC_Os08g38920	caffeoyl-CoA O-methyltransferase, putative	2	1.06		
LOC_Os08g39330	skin secretory protein xP2 precursor, putative	2			0.97
LOC_Os08g40380	sulfotransferase domain containing protein	2	1.04	-0.95	0.8
LOC_Os08g41500	ubiquitin-associated protein, putative	2			1.33
LOC_Os08g41730	peptidase, T1 family, putative	2		1.31	1.44
LOC_Os08g42010	nodulin, putative	2			0.86
LOC_Os08g42110	expressed protein	2			0.9
LOC_Os08g42189	expressed protein	2			1.34
LOC_Os08g42268	expressed protein	2			1.35
LOC_Os08g42320	expressed protein	2			1.24
LOC_Os08g43390	cytochrome P450, putative	2			1.31
LOC_Os08g43440	cytochrome P450, putative	2	0.75		
LOC_Os08g44750	auxin-induced protein 5NG4, putative	2	0.99		0.71
LOC_Os09g08130	indole-3-glycerol phosphate synthase, chloroplast precursor, putative	2	0.94	0.86	0.91
LOC_Os09g09690	retrotransposon protein, putative, Ty3-gypsy subclass	2	0.75		

LOC_Os09g10010	expressed protein	2	0.93		1.02
LOC_Os09g12150	OsFBX310 - F-box domain containing protein	2		0.85	
LOC_Os09g12970	plant protein of unknown function domain containing protein	2			2.67
LOC_Os09g16280	hydroxyproline-rich glycoprotein family protein, putative	2			0.91
LOC_Os09g17190	OsFBX320 - F-box domain containing protein	2			0.81
LOC_Os09g19970	expressed protein	2		0.7	0.91
LOC_Os09g20220	glutathione S-transferase, putative	2	0.85	2.42	2.76
LOC_Os09g20430	SCO1 protein homolog, mitochondrial precursor, putative	2		0.91	1.33
LOC_Os09g21710	AN1-like zinc finger domain containing protein	2		0.99	1.43
LOC_Os09g27260	plant viral response family protein, putative	2		0.86	1.19
LOC_Os09g27510	cytochrome P450, putative	2		0.83	0.99
LOC_Os09g27730	HVA22, putative	2			1.49
LOC_Os09g28210	bHelix-loop-helix transcription factor, putative	2	0.71		
LOC_Os09g31031	ubiquitin family protein, putative	2			1.27
LOC_Os09g31430	Os9bglu30 - beta-glucosidase, similar to Os4bglu12 exoglucanase	2	1.56		0.77
LOC_Os09g32290	FAD dependent oxidoreductase domain containing protein	2			0.75
LOC_Os09g33490	no apical meristem protein, putative	2	0.74		
LOC_Os09g34890	expressed protein	2	1.05		
LOC_Os09g35940	cytochrome P450, putative	2			1,00
LOC_Os09g37540	uncharacterized protein PA4923, putative	2	1.04		
LOC_Os09g38210	auxin efflux carrier component, putative	2	0.98		0.74
LOC_Os09g39410	male sterility protein, putative	2	0.74		1.05
LOC_Os10g03850	OsFBX352 - F-box domain containing protein	2			1.27
LOC_Os10g04800	hypothetical protein	2	1.56		1.12
LOC_Os10g06030	OsWAK103 - OsWAK receptor-like protein kinase	2	0.92		
LOC_Os10g07210	hsp20/alpha crystallin family protein, putative	2			1.74
LOC_Os10g08640	hypothetical protein	2			1.83
LOC_Os10g09110	cytochrome P450, putative	2			1.38
LOC_Os10g15164	expressed protein	2			2.04
LOC_Os10g16974	cytochrome P450, putative	2			0.72
LOC_Os10g17790	remorin C-terminal domain containing protein, putative	2			1.59
LOC_Os10g20350	MATE efflux family protein, putative	2	0.78		0.97
LOC_Os10g21670	dehydration stress-induced protein, putative	2	0.95		
LOC_Os10g22520	cellulase, putative	2		0.9	
LOC_Os10g25090	STRUBBELIG-RECEPTOR FAMILY 6 precursor, putative	2			0.7
LOC_Os10g25870	dirigent, putative	2			0.72
LOC_Os10g27280	thaumatin, putative	2	0.71		
LOC_Os10g28080	glycosyl hydrolase, putative	2	0.86		0.98
LOC_Os10g28120	glycosyl hydrolase, putative	2	1.39		1.98
LOC_Os10g30180	hsp20/alpha crystallin family protein, putative	2			0.95
LOC_Os10g30410	cytochrome P450 71D7, putative	2	0.88		
LOC_Os10g30690	MYB family transcription factor, putative	2			0.74
LOC_Os10g31530	glycine-rich cell wall protein, putative	2			1.23
LOC_Os10g32700	hypersensitive-induced response protein, putative	2			0.85
LOC_Os10g32980	CESA7 - cellulose synthase	2	0.91		
LOC_Os10g34450	hypothetical protein	2			0.92
LOC_Os10g34480	cytochrome P450, putative	2	1.28		0.98
LOC_Os10g34910	secretory protein, putative	2	1.12		0.83
LOC_Os10g34920	secretory protein, putative	2			0.79
LOC_Os10g34930	secretory protein, putative	2	1.11		
LOC_Os10g35120	hypothetical protein	2	0.88		
LOC_Os10g36650	actin, putative	2	0.73		
LOC_Os10g36960	transposon protein, putative, unclassified	2	0.8		
LOC_Os10g36980	transposon protein, putative, unclassified	2	0.74		

LOC_Os10g37160	transposon protein, putative, unclassified	2	1.38		
LOC_Os10g37400	DUF538 domain containing protein, putative	2	0.84		
LOC_Os10g38160	glutathione S-transferase, putative	2	1.52	1.84	2.71
LOC_Os10g38340	glutathione S-transferase GSTU6, putative	2	1.44	1.73	2.77
LOC_Os10g38360	glutathione S-transferase, putative	2	0.97	1.1	2.27
LOC_Os10g38470	glutathione S-transferase, putative	2			1.01
LOC_Os10g38489	glutathione S-transferase GSTU6, putative	2	1.45	1.89	2.66
LOC_Os10g38600	glutathione S-transferase GSTU6, putative	2	1.26	1.42	2.37
LOC_Os10g38700	glutathione S-transferase, putative	2			0.7
LOC_Os10g38740	glutathione S-transferase, putative	2	0.78		0.92
LOC_Os10g38860	hydrolase, alpha/beta fold family domain containing protein	2	0.78		
LOC_Os10g38880	DUF623 domain containing protein	2	0.75		
LOC_Os10g39300	aspartic proteinase nepenthesin, putative	2			3.07
LOC_Os10g39640	expansin precursor, putative	2			0.79
LOC_Os10g39680	CHIT14 - Chitinase family protein precursor	2	0.89		
LOC_Os10g39700	CHIT15 - Chitinase family protein precursor, putative	2	0.82		
LOC_Os10g39890	POEI31 - Pollen Ole e I allergen and extensin family protein precursor, putative	2	1.02		0.87
LOC_Os10g40510	LTPL144 - Protease inhibitor/seed storage/LTP family protein precursor	2	0.92		0.93
LOC_Os10g40720	expansin precursor, putative	2	1,00		
LOC_Os10g42040	expressed protein	2	1.03		
LOC_Os11g02379	LTPL6 - Protease inhibitor/seed storage/LTP family protein precursor	2			0.82
LOC_Os11g02440	chalcone--flavonone isomerase, putative	2	0.72		
LOC_Os11g03290	nucleoside-triphosphatase, putative	2	1.73		0.92
LOC_Os11g03970	CAMK_KIN1/SNF1/Nim1_like.5 - CAMK includes calcium/calmodulin depedent protein kinases	2			1.2
LOC_Os11g04104	major facilitator superfamily antiporter, putative	2			0.71
LOC_Os11g04290	cytochrome P450, putative	2	0.85	1.31	1.81
LOC_Os11g04560	calmodulin-like protein 1, putative	2	0.8		0.98
LOC_Os11g05290	stress responsive A/B Barrel domain containing protein	2	0.76		
LOC_Os11g06150	basic proline-rich protein precursor, putative	2		0.75	
LOC_Os11g08120	Os11bglu37 - GH1 pseudogene	2	1.01		
LOC_Os11g08380	1-aminocyclopropane-1-carboxylate oxidase, putative	2			0.76
LOC_Os11g09850	expressed protein	2			2.84
LOC_Os11g09940	expressed protein	2			2.17
LOC_Os11g11940	MLA10, putative	2			1.29
LOC_Os11g11950	disease resistance protein RPM1, putative	2			1.35
LOC_Os11g12000	NBS-LRR disease resistance protein, putative	2			1.38
LOC_Os11g12760	O-methyltransferase, putative	2	1.63	-0.84	
LOC_Os11g14910	NADP-dependent oxidoreductase, putative	2			0.8
LOC_Os11g16970	carnitine racemase like protein, putative	2	0.95		
LOC_Os11g17014	NB-ARC domain containing protein	2	0.76		
LOC_Os11g17504	retrotransposon protein, putative, Ty1-copia subclass	2			0.84
LOC_Os11g18980	conserved hypothetical protein	2			1.91
LOC_Os11g20090	O-methyltransferase, putative	2	2.05	-1.01	0.87
LOC_Os11g20160	O-methyltransferase, putative	2	1.37		
LOC_Os11g28430	expressed protein	2			0.75
LOC_Os11g28940	expressed protein	2			0.71
LOC_Os11g30810	sulfotransferase domain containing protein	2	1.05		0.85
LOC_Os11g31090	transferase family protein, putative	2	0.98		
LOC_Os11g31540	BRASSINOSTEROID INSENSITIVE 1-associated receptor kinase 1 precursor, putative	2	1.07	0.77	1.13
LOC_Os11g31940	GDSL-like lipase/acylhydrolase, putative	2	0.77		
LOC_Os11g32650	chalcone synthase, putative	2	0.71		1.03
LOC_Os11g32780	expressed protein	2		0.82	
LOC_Os11g35040	aminotransferase, classes I and II, domain containing protein	2			0.82
LOC_Os11g36790	OsFBO6 - F-box and other domain containing protein	2			0.71

LOC_Os11g37900	3-ketoacyl-CoA synthase, putative	2	0.85		
LOC_Os11g38480	NBS-LRR type disease resistance protein, putative	2			0.81
LOC_Os11g39290	Leucine Rich Repeat family protein	2			1.17
LOC_Os11g39320	LZ-NBS-LRR class, putative	2			0.78
LOC_Os11g39370	BRASSINOSTEROID INSENSITIVE 1-associated receptor kinase 1 precursor, putative	2			0.89
LOC_Os11g39540	14-3-3 protein, putative	2			0.92
LOC_Os11g41410	expressed protein	2			0.78
LOC_Os11g42170	expressed protein	2			0.79
LOC_Os11g42550	dirigent, putative	2			0.75
LOC_Os11g42950	expressed protein	2			1.34
LOC_Os11g42989	exo70 exocyst complex subunit, putative	2		0.78	
LOC_Os11g44970	NBS-LRR disease resistance protein, putative	2			0.98
LOC_Os11g45090	NB-ARC domain containing protein	2			0.81
LOC_Os11g45970	NBS-LRR disease resistance protein, putative	2			1.02
LOC_Os11g48060	laccase-22 precursor, putative	2		0.78	
LOC_Os12g01740	serine/threonine-protein kinase, putative	2	0.75		0.74
LOC_Os12g03810	CAMK_KIN1/SNF1/Nim1_like.37 - CAMK includes calcium/calmodulin depedent protein kinases	2			0.95
LOC_Os12g04100	cytochrome P450, putative	2	0.95	1.16	2.1
LOC_Os12g04150	alpha/beta hydrolase fold, putative	2	0.83		
LOC_Os12g05510	conserved hypothetical protein	2		0.87	1.45
LOC_Os12g05880	Cupin domain containing protein	2			0.84
LOC_Os12g07310	citrate-binding protein precursor, putative	2	1.61		1.18
LOC_Os12g10220	expressed protein	2	0.73		
LOC_Os12g11550	hypothetical protein	2	1.31		
LOC_Os12g11620	hypothetical protein	2	1.9	1.12	0.79
LOC_Os12g11980	hypothetical protein	2	1.16		
LOC_Os12g14540	expressed protein	2			0.88
LOC_Os12g15680	laccase precursor protein, putative	2	0.72	1.33	2.28
LOC_Os12g16010	sex determination protein tasselseed-2, putative	2	0.79		
LOC_Os12g16720	cytochrome P450 71A1, putative	2	0.89	0.87	0.94
LOC_Os12g24650	leucine aminopeptidase, chloroplast precursor, putative	2			0.86
LOC_Os12g25090	expressed protein	2			0.83
LOC_Os12g26290	alpha-DOX2, putative	2	0.86		
LOC_Os12g26510	expressed protein	2	1.38		1.27
LOC_Os12g26960	THION34 - Plant thionin family protein precursor	2	1,00		2.37
LOC_Os12g30824	terpene synthase, putative	2			0.97
LOC_Os12g32640	haemolysin-III, putative	2			0.87
LOC_Os12g36240	inhibitor I family protein, putative	2			0.74
LOC_Os12g36830	pathogenesis-related Bet v I family protein, putative	2	1.28		
LOC_Os12g36840	pathogenesis-related Bet v I family protein, putative	2	0.86		
LOC_Os12g36850	pathogenesis-related Bet v I family protein, putative	2	0.89		
LOC_Os12g36860	pathogenesis-related protein 10, putative	2	1.39		
LOC_Os12g36880	pathogenesis-related Bet v I family protein, putative	2	1.71		
LOC_Os12g38290	metallothionein, putative	2	0.83	-1.06	0.9
LOC_Os12g38670	transposon protein, putative, CACTA, En/Spm sub-class	2		0.74	
LOC_Os12g41860	START domain containing protein	2	0.88		
LOC_Os12g42040	OsWAK126 - OsWAK receptor-like protein kinase	2			1.31
LOC_Os12g43380	thaumatin, putative	2	1.08		
LOC_Os12g43390	thaumatin, putative	2	0.99		
LOC_Os12g43490	thaumatin, putative	2			0.85
LOC_Os01g01840	helix-loop-helix DNA-binding domain containing protein	3			-2.53
LOC_Os01g02430	TAK14, putative	3			-1.5
LOC_Os01g02700	protein kinase domain containing protein	3			-2.41
LOC_Os01g02830	receptor-like kinase ARK1AS, putative	3			-1.17

LOC_Os01g05150	expressed protein	3			-1.97
LOC_Os01g05540	expressed protein	3			-1.19
LOC_Os01g05585	metallothionein, putative	3			-1.5
LOC_Os01g05630	Core histone H2A/H2B/H3/H4 domain containing protein, putative	3			-1.49
LOC_Os01g05650	metallothionein, putative	3			-1.51
LOC_Os01g06310	glycine-rich cell wall structural protein precursor, putative	3			-1.11
LOC_Os01g07770	peroxidase precursor, putative	3			-1.01
LOC_Os01g09030	2-aminoethanethiol dioxygenase, putative	3			-0.86
LOC_Os01g09370	ankyrin repeat domain-containing protein 28, putative	3			-0.87
LOC_Os01g09540	HAD superfamily phosphatase, putative	3			-0.94
LOC_Os01g11570	GDSL-like lipase/acylhydrolase, putative	3			-1.52
LOC_Os01g12000	expressed protein	3			-0.71
LOC_Os01g15340	flowering promoting factor-like 1, putative	3			-1.4
LOC_Os01g15979	gb protein, putative	3			-0.77
LOC_Os01g21590	homeodomain, putative	3	-0.93		-0.72
LOC_Os01g33960	transposon protein, putative, CACTA, En/Spm sub-class	3			-0.99
LOC_Os01g38650	expressed protein	3			-1.95
LOC_Os01g39080	hypothetical protein	3			-2.04
LOC_Os01g42690	OsPOP2 - Putative Prolyl Oligopeptidase homologue	3			-0.73
LOC_Os01g48620	expressed protein	3			-0.7
LOC_Os01g48960	glutamate synthase, chloroplast precursor, putative	3		-1.33	-1.49
LOC_Os01g49640	LTPL149 - Protease inhibitor/seed storage/LTP family protein precursor	3			-1.16
LOC_Os01g49670	cytidylyltransferase domain containing protein	3			-1.27
LOC_Os01g51290	protein kinase family protein, putative	3			-1.37
LOC_Os01g51370	expressed protein	3			-0.8
LOC_Os01g51670	expressed protein	3			-2.6
LOC_Os01g51840	IQ calmodulin-binding motif family protein, putative	3			-0.82
LOC_Os01g52130	sulfate transporter, putative	3			-1.48
LOC_Os01g52140	expressed protein	3			-0.71
LOC_Os01g52250	starch synthase, putative	3			-0.72
LOC_Os01g52260	serine acetyltransferase protein, putative	3			-0.78
LOC_Os01g53800	glutamate carboxypeptidase 2, putative	3			-0.71
LOC_Os01g55240	gibberellin 2-beta-dioxygenase, putative	3			-0.9
LOC_Os01g55540	aminotransferase, classes I and II, domain containing protein	3			-0.87
LOC_Os01g56030	EF hand family protein, putative	3			-0.87
LOC_Os01g56270	transposon protein, putative, CACTA, En/Spm sub-class	3			-2.29
LOC_Os01g56480	expressed protein	3			-1.2
LOC_Os01g57710	membrane protein, putative	3			-0.75
LOC_Os01g59440	BRASSINOSTEROID INSENSITIVE 1-associated receptor kinase 1 precursor, putative	3			-0.72
LOC_Os01g59660	MYB family transcription factor, putative	3			-1.15
LOC_Os01g59930	NADH-cytochrome b5 reductase, putative	3			-1.69
LOC_Os01g60530	transposon protein, putative, CACTA, En/Spm sub-class	3			-1.18
LOC_Os01g61070	heavy metal-associated domain containing protein	3			-1.19
LOC_Os01g61380	lactate/malate dehydrogenase, putative	3			-1.21
LOC_Os01g62100	retrotransposon protein, putative, unclassified	3			-1.59
LOC_Os01g62790	expressed protein	3			-1.39
LOC_Os01g62830	expressed protein	3			-0.85
LOC_Os01g62920	homeodomain protein, putative	3			-0.93
LOC_Os01g63250	josephin, putative	3			-0.81
LOC_Os01g63260	3-oxo-5-alpha-steroid 4-dehydrogenase, putative	3			-1.24
LOC_Os01g63900	WD domain, G-beta repeat domain containing protein	3			-1.83
LOC_Os01g63970	sialyltransferase family domain containing protein	3			-0.89
LOC_Os01g64520	uricase, putative	3	-1.03	-0.77	-1.1
LOC_Os01g64680	bolA, putative	3			-1.06

LOC_Os01g64690	LSM domain containing protein	3			-0.86
LOC_Os01g65169	proton-dependent oligopeptide transport, putative	3			-1.47
LOC_Os01g65260	amidophosphoribosyltransferase, chloroplast precursor, putative	3			-0.98
LOC_Os01g65310	DUF803 domain containing, putative	3			-0.71
LOC_Os01g65692	expressed protein	3			-1.07
LOC_Os01g65830	acyl-desaturase, chloroplast precursor, putative	3			-0.87
LOC_Os01g65986	DUF803 domain containing, putative	3			-0.75
LOC_Os01g66180	cytochrome c, putative	3			-0.83
LOC_Os01g66890	BTBZ1 - Bric-a-Brac, Tramtrack, and Broad Complex BTB domain with TAZ zinc finger and Calmodulin-binding domains	3			-0.72
LOC_Os01g66900	expressed protein	3			-0.82
LOC_Os01g67360	methyltransferase, putative	3	-0.81		-1.02
LOC_Os01g70730	flowering promoting factor-like 1, putative	3			-0.74
LOC_Os01g73670	expressed protein	3			-0.73
LOC_Os01g74450	aquaporin protein, putative	3			-0.88
LOC_Os02g03150	inhibitor I family protein, putative	3	-0.95		-0.71
LOC_Os02g06580	formin, putative	3			-1.28
LOC_Os02g06770	expressed protein	3			-0.87
LOC_Os02g07700	expressed protein	3			-0.88
LOC_Os02g12150	expressed protein	3			-0.86
LOC_Os02g12760	integral membrane protein, putative	3			-0.71
LOC_Os02g13100	protein phosphatase 2C, putative	3			-0.77
LOC_Os02g13510	receptor-like protein kinase 5 precursor, putative	3	0.72		-0.72
LOC_Os02g18690	BURP domain containing protein	3			-1.59
LOC_Os02g18750	expressed protein	3			-1.42
LOC_Os02g20330	conserved hypothetical protein	3		-0.86	-1.02
LOC_Os02g21430	AML1, putative	3			-0.84
LOC_Os02g26790	expressed protein	3			-3.36
LOC_Os02g28030	hypothetical protein	3			-1.09
LOC_Os02g28074	XRN 5-3 exonuclease N-terminus family protein	3			-1.7
LOC_Os02g28465	expressed protein	3			-1.18
LOC_Os02g30310	ThiF family domain containing protein, putative	3			-0.8
LOC_Os02g30470	expressed protein	3			-1.88
LOC_Os02g30530	transposon protein, putative, unclassified	3			-0.76
LOC_Os02g30620	dnaJ domain containing protein	3			-0.73
LOC_Os02g30640	basic salivary proline-rich protein 2 precursor, putative	3			-0.93
LOC_Os02g30790	SAR DNA-binding protein-like, putative	3			-1.34
LOC_Os02g30810	OsSAUR10 - Auxin-responsive SAUR gene family member	3			-1.14
LOC_Os02g32009	expressed protein	3			-0.98
LOC_Os02g32660	1,4-alpha-glucan-branching enzyme, chloroplast precursor, putative	3			-0.7
LOC_Os02g32670	retrotransposon protein, putative, unclassified	3			-1.06
LOC_Os02g34700	conserved hypothetical protein	3			-1.01
LOC_Os02g34760	retrotransposon protein, putative, unclassified	3			-0.76
LOC_Os02g37090	hydrolase, alpha/beta fold family domain containing protein	3			-1.11
LOC_Os02g39620	ATOZI1, putative	3			-1.38
LOC_Os02g42200	transcription factor-related, putative	3			-0.74
LOC_Os02g43540	retrotransposon protein, putative, unclassified	3			-1.3
LOC_Os02g44880	expressed protein	3			-0.81
LOC_Os02g48150	expressed protein	3			-0.92
LOC_Os02g52360	peptidyl-prolyl cis-trans isomerase CYP40, putative	3			-0.87
LOC_Os02g52930	integral membrane protein DUF6 containing protein	3			-0.77
LOC_Os02g54880	cytochrome c oxidase subunit, putative	3	-0.76		-1.18
LOC_Os02g55890	inorganic H+ pyrophosphatase, putative	3			-1.32
LOC_Os02g56250	GATA zinc finger domain containing protein	3			-0.78
LOC_Os03g01700	expressed protein	3			-0.76

LOC_Os03g02939	peroxidase precursor, putative	3		-0.74	-0.94
LOC_Os03g03260	homeobox domain containing protein	3			-0.77
LOC_Os03g03390	G-patch domain containing protein	3			-2.18
LOC_Os03g03490	expressed protein	3			-2.09
LOC_Os03g03500	heavy metal-associated domain containing protein	3			-1.15
LOC_Os03g07750	expressed protein	3			-2.23
LOC_Os03g08020	elongation factor Tu, putative	3			-4.29
LOC_Os03g08490	AP2 domain containing protein	3			-2.73
LOC_Os03g08720	transferase family protein, putative	3			-1.07
LOC_Os03g11734	MATE efflux protein, putative	3			-0.9
LOC_Os03g12790	MATE efflux family protein, putative	3			-1.03
LOC_Os03g13210	peroxidase precursor, putative	3			-0.78
LOC_Os03g19370	CPuORF4 - conserved peptide uORF-containing transcript	3			-0.7
LOC_Os03g21050	retrotransposon protein, putative, Ty1-copia subclass	3			-1.16
LOC_Os03g22170	AP2 domain containing protein	3			-1.13
LOC_Os03g31750	pyruvate, phosphate dikinase, chloroplast precursor, putative	3			-1.52
LOC_Os03g33570	calcineurin B-like protein 8, putative	3			-0.86
LOC_Os03g40070	transposon protein, putative, unclassified	3			-0.77
LOC_Os03g41229	expressed protein	3			-1.17
LOC_Os03g42235	expressed protein	3			-2.25
LOC_Os03g43100	expressed protein	3			-3.23
LOC_Os03g48430	1-aminocyclopropane-1-carboxylate oxidase homolog 4, putative	3			-0.85
LOC_Os03g51020	expressed protein	3		-0.73	-0.89
LOC_Os03g52180	4-hydroxy-3-methylbut-2-enyl diphosphate reductase, putative	3			-0.7
LOC_Os03g52690	CBS domain containing membrane protein, putative	3			-0.75
LOC_Os03g61780	glucan endo-1,3-beta-glucosidase-related, putative	3			-0.8
LOC_Os04g03579	protein kinase, putative	3			-1.28
LOC_Os04g13140	vignain precursor, putative	3			-1.25
LOC_Os04g25540	ribosomal protein L27, putative	3			-1.39
LOC_Os04g33150	desiccation-related protein PCC13-62 precursor, putative	3			-1.78
LOC_Os04g36740	potassium channel SKOR, putative	3			-1.19
LOC_Os04g36750	hsp20/alpha crystallin family protein, putative	3			-1.48
LOC_Os04g37990	transporter family protein, putative	3	-0.71		-1.14
LOC_Os04g44060	aquaporin protein, putative	3			-0.85
LOC_Os04g45510	expressed protein	3			-0.81
LOC_Os04g45520	integral membrane protein, putative	3			-1.75
LOC_Os04g47810	ethylene-responsive protein related, putative	3			-0.95
LOC_Os04g49194	naringenin,2-oxoglutarate 3-dioxygenase, putative	3			-0.7
LOC_Os04g51030	wall-associated kinase 1, putative	3			-0.92
LOC_Os04g51809	expressed protein	3			-1.81
LOC_Os04g52640	SHR5-receptor-like kinase, putative	3			-1.79
LOC_Os04g52660	expressed protein	3			-1.11
LOC_Os04g53060	NBS-LRR disease resistance protein, putative	3			-1.02
LOC_Os04g53830	3-beta hydroxysteroid dehydrogenase/isomerase family protein, putative	3			-0.93
LOC_Os04g53850	leucoanthocyanidin reductase, putative	3			-2.76
LOC_Os04g54310	conserved hypothetical protein	3			-0.81
LOC_Os04g54820	programmed cell death 2 C-terminal domain-containing protein, putative	3			-1.48
LOC_Os04g55500	expressed protein	3			-1.34
LOC_Os04g55740	peroxidase precursor, putative	3			-0.84
LOC_Os04g55810	expressed protein	3			-0.78
LOC_Os04g59020	integral membrane protein, putative	3			-0.99
LOC_Os05g03820	glutamate--cysteine ligase, chloroplast precursor, putative	3			-0.95
LOC_Os05g05060	expressed protein	3	-0.81		-0.75
LOC_Os05g05640	pectinesterase inhibitor domain containing protein, putative	3			-0.89

LOC_Os05g07810	universal stress protein domain containing protein, putative	3			-1.18
LOC_Os05g09480	OsIAA16 - Auxin-responsive Aux/IAA gene family member	3			-0.94
LOC_Os05g11130	cytochrome P450, putative	3			-0.75
LOC_Os05g12280	clumping factor A precursor, putative	3			-0.8
LOC_Os05g29790	pectinesterase, putative	3			-0.78
LOC_Os05g30680	leucine zipper protein-like, putative	3			-0.77
LOC_Os05g36260	soluble inorganic pyrophosphatase, putative	3			-0.71
LOC_Os05g39310	thiamine pyrophosphate enzyme, C-terminal TPP binding domain containing protein	3			-0.8
LOC_Os05g39320	thiamine pyrophosphate enzyme, C-terminal TPP binding domain containing protein	3			-2.16
LOC_Os05g40820	ribosomal protein L24, putative	3			-1.24
LOC_Os05g46630	expressed protein	3			-1.62
LOC_Os05g46720	phosphatidylinositol transfer, putative	3			-1.96
LOC_Os05g46800	hypothetical protein	3			-2.82
LOC_Os05g46845	conserved hypothetical protein	3			-1.14
LOC_Os05g46890	expressed protein	3			-4.13
LOC_Os05g46950	expressed protein	3			-1.58
LOC_Os05g46954	expressed protein	3			-3.78
LOC_Os05g48740	expressed protein	3	-1.37		-1.31
LOC_Os05g48810	dnaJ domain containing protein	3			-0.84
LOC_Os05g48940	expressed protein	3			-0.73
LOC_Os05g49540	retrotransposon protein, putative, Ty1-copia subclass	3			-1.34
LOC_Os05g49880	lactate/malate dehydrogenase, putative	3			-0.7
LOC_Os05g50750	AAA family ATPase, putative	3	-0.8	-0.85	-1.42
LOC_Os06g04070	pyridoxal-dependent decarboxylase protein, putative	3	-0.77		-0.85
LOC_Os06g07040	OsIAA20 - Auxin-responsive Aux/IAA gene family member	3			-0.79
LOC_Os06g08154	receptor-like protein kinase 5 precursor, putative	3			-0.7
LOC_Os06g10870	retrotransposon protein, putative, unclassified	3	-1.02		-1.09
LOC_Os06g11440	transposon protein, putative, Mutator sub-class	3			-0.71
LOC_Os06g11812	wound/stress protein, putative	3			-0.86
LOC_Os06g13140	WD domain, G-beta repeat domain containing protein	3			-0.73
LOC_Os06g13810	pyrophosphate--fructose 6-phosphate 1-phosphotransferase subunit beta, putative	3			-0.95
LOC_Os06g15380	acylphosphatase, putative	3	-0.73		-0.79
LOC_Os06g31220	mitogen-activated protein kinase 17, putative	3			-2.71
LOC_Os06g31800	THION2 - Plant thionin family protein precursor	3		-1.89	-3,00
LOC_Os06g32160	THION7 - Plant thionin family protein precursor	3		-1.54	-4.88
LOC_Os06g32240	THION9 - Plant thionin family protein precursor	3		-1.46	-3.91
LOC_Os06g33180	expressed protein	3			-0.74
LOC_Os06g33549	protein phosphatase 2C, putative	3			-0.93
LOC_Os06g34330	expressed protein	3			-0.74
LOC_Os06g35060	heavy metal-associated domain containing protein	3			-0.76
LOC_Os06g35160	CAMK_KIN1/SNF1/Nim1_like.26 - CAMK includes calcium/calmodulin depedent protein kinases	3			-0.86
LOC_Os06g35530	CGMC_GSK.8 - CGMC includes CDA, MAPK, GSK3, and CLKC kinases	3			-1.81
LOC_Os06g35560	reticuline oxidase-like protein precursor, putative	3			-0.89
LOC_Os06g36450	ferroportin1 domain containing protein	3			-0.7
LOC_Os06g37080	L-ascorbate oxidase precursor, putative	3			-0.82
LOC_Os06g39120	expressed protein	3			-1.22
LOC_Os06g40060	glycosyltransferase family protein, putative	3			-0.95
LOC_Os06g41120	expressed protein	3			-3.73
LOC_Os06g41240	expressed protein	3			-1.93
LOC_Os06g45860	molybdenum cofactor sulfurase, putative	3			-0.84
LOC_Os06g46754	RIC10, putative	3			-0.86

LOC_Os06g48010	peroxidase precursor, putative	3			-0.99
LOC_Os06g50480	bZIP transcription factor domain containing protein	3			-1.07
LOC_Os06g50600	bZIP transcription factor domain containing protein	3			-1.11
LOC_Os06g50630	conserved hypothetical protein	3			-1.56
LOC_Os07g03200	phytosulfokines precursor, putative	3			-0.76
LOC_Os07g06930	hypothetical protein	3			-1.06
LOC_Os07g09914	expressed protein	3			-0.84
LOC_Os07g12390	expressed protein	3			-1.34
LOC_Os07g15370	metal transporter Nramp6, putative	3			-1.19
LOC_Os07g16600	expressed protein	3			-4.73
LOC_Os07g17130	FYVE zinc finger domain containing protein	3			-1.1
LOC_Os07g17260	retrotransposon protein, putative, unclassified	3			-0.71
LOC_Os07g17970	AMP-binding domain containing protein	3			-1.52
LOC_Os07g18162	aldehyde oxidase, putative	3			-0.84
LOC_Os07g18250	expressed protein	3			-0.96
LOC_Os07g18930	expressed protein	3			-1.96
LOC_Os07g18990	LTPL40 - Protease inhibitor/seed storage/LTP family protein precursor	3			-1.57
LOC_Os07g20164	expressed protein	3			-4.74
LOC_Os07g22350	glucose-6-phosphate 1-dehydrogenase, chloroplast precursor, putative	3			-1.4
LOC_Os07g22390	ankyrin repeat domain containing protein	3			-2.61
LOC_Os07g22494	expressed protein	3			-1.1
LOC_Os07g22720	2-oxo acid dehydrogenases acyltransferase domain containing protein	3			-1.11
LOC_Os07g22950	adenylate kinase isoenzyme 6, putative	3			-0.81
LOC_Os07g23640	retrotransposon protein, putative, Ty3-gypsy subclass	3			-1.6
LOC_Os07g23660	retrotransposon protein, putative, unclassified	3			-2.23
LOC_Os07g24820	thionin-like peptide, putative	3		-0.9	-1.87
LOC_Os07g24830	thionin-like peptide, putative	3			-5.4
LOC_Os07g25150	myb-related protein 306, putative	3		-0.72	-1.39
LOC_Os07g25410	peptidase, M24 family protein, putative	3			-1.19
LOC_Os07g28890	ethylene-responsive protein related, putative	3			-0.85
LOC_Os07g31450	CHR4/MI-2-LIKE, putative	3		-1.1	-1.59
LOC_Os07g32430	pre-mRNA-splicing factor ATP-dependent RNA helicase PRP16, putative	3			-1.5
LOC_Os07g32650	retrotransposon protein, putative, unclassified	3			-1.15
LOC_Os07g32950	dnaJ C terminal region family protein	3			-0.95
LOC_Os07g33480	cytochrome P450 domain containing protein	3			-2.25
LOC_Os07g33680	succinate dehydrogenase subunit 3, putative	3			-4.49
LOC_Os07g33690	NBS-LRR type disease resistance protein Hom-F, putative	3			-1.04
LOC_Os07g33780	pleiotropic drug resistance protein 5, putative	3	0.85		-1.37
LOC_Os07g33790	glutamate receptor 3.4 precursor, putative	3			-1.09
LOC_Os07g41350	B12D protein, putative	3			-0.78
LOC_Os08g02110	peroxidase precursor, putative	3			-1.04
LOC_Os08g02420	OsCML7 - Calmodulin-related calcium sensor protein	3			-0.71
LOC_Os08g03030	retrotransposon protein, putative, Ty1-copia subclass	3			-1.1
LOC_Os08g06170	berberine and berberine like domain containing protein	3			-1.16
LOC_Os08g07010	ABC-2 type transporter domain containing protein	3			-0.79
LOC_Os08g07790	CRS2-associated factor 2, mitochondrial precursor, putative	3			-0.81
LOC_Os08g07840	PolI-like DNA polymerase, putative	3			-0.83
LOC_Os08g07890	NB-ARC domain containing protein	3			-0.72
LOC_Os08g07970	transcription factor, putative	3			-0.71
LOC_Os08g08360	expressed protein	3			-1.05
LOC_Os08g08570	expressed protein	3			-3.64
LOC_Os08g08592	expressed protein	3			-1.32
LOC_Os08g08650	expressed protein	3			-1.09
LOC_Os08g08700	ubiquitin family protein, putative	3			-1.83

LOC_Os08g09210	phosphoribosylamine--glycine ligase, putative	3		-1.74
LOC_Os08g09610	expressed protein	3		-1.08
LOC_Os08g09900	transposon protein, putative, unclassified	3		-1.29
LOC_Os08g10100	expressed protein	3		-1.4
LOC_Os08g10150	SHR5-receptor-like kinase, putative	3		-1.14
LOC_Os08g10300	SHR5-receptor-like kinase, putative	3		-1.17
LOC_Os08g10320	SHR5-receptor-like kinase, putative	3		-2.05
LOC_Os08g10510	aminotransferase, putative	3		-0.99
LOC_Os08g10550	potassium transporter, putative	3		-0.98
LOC_Os08g13060	MBTB22 - Bric-a-Brac, Tramtrack, Broad Complex BTB domain with Meprin and TRAF Homology MATH domain	3		-0.84
LOC_Os08g13280	expressed protein	3		-0.87
LOC_Os08g13469	expressed protein	3		-2.27
LOC_Os08g13640	ankyrin, putative	3		-1.33
LOC_Os08g13690	60S ribosomal protein L7, putative	3		-0.77
LOC_Os08g13699	expressed protein	3		-0.75
LOC_Os08g13710	expressed protein	3		-1.77
LOC_Os08g13840	OsWRKY44 - Superfamily of TFs having WRKY and zinc finger domains	3		-3.1
LOC_Os08g13920	glycosyl hydrolases family 16, putative	3		-2.17
LOC_Os08g14020	retrotransposon protein, putative, Ty1-copia subclass	3	-0.8	-1.82
LOC_Os08g14050	expressed protein	3		-0.79
LOC_Os08g14640	syntaxin 6, N-terminal domain containing protein	3		-1.13
LOC_Os08g14810	NB-ARC domain containing protein	3		-3.15
LOC_Os08g14860	cytochrome b-c1 complex subunit 7, putative	3		-1.22
LOC_Os08g14880	transposon protein, putative, unclassified	3		-3.47
LOC_Os08g14880	transposon protein, putative, unclassified	3		-1.25
LOC_Os08g15204	thioredoxin domain-containing protein 9, putative	3		-1.63
LOC_Os08g15444	expressed protein	3		-3.13
LOC_Os08g15590	Leucine rich repeat N-terminal domain containing protein, putative	3		-0.71
LOC_Os08g16050	tetraspanin family protein, putative	3		-1.33
LOC_Os08g16130	fiber protein Fb34, putative	3		-0.74
LOC_Os08g16350	expressed protein	3		-1.72
LOC_Os08g16720	retrotransposon protein, putative, unclassified	3		-1.04
LOC_Os08g17060	expressed protein	3		-1.08
LOC_Os08g17370	transmembrane 9 superfamily member, putative	3		-1.53
LOC_Os08g19250	retrotransposon protein, putative, Ty1-copia subclass	3		-1.09
LOC_Os08g19320	aberrant root formation protein 4, putative	3		-0.8
LOC_Os08g19370	expressed protein	3		-0.71
LOC_Os08g19374	expressed protein	3		-3.05
LOC_Os08g20200	male sterility protein, putative	3	-0.85	-0.72
LOC_Os08g20660	sucrose-phosphate synthase, putative	3		-1.14
LOC_Os08g20680	expressed protein	3		-2.95
LOC_Os08g22149	CBS domain containing membrane protein, putative	3		-1.67
LOC_Os08g23680	FHA domain containing protein, putative	3		-0.77
LOC_Os08g23870	late embryogenesis abundant group 1, putative	3		-0.83
LOC_Os08g25050	PIF-like orf1, putative	3		-1.03
LOC_Os08g25624	phosphate/phosphate translocator, putative	3		-0.94
LOC_Os08g25720	pyrophosphate--fructose 6-phosphate 1-phosphotransferase subunit alpha, putative	3		-0.81
LOC_Os08g26700	expressed protein	3		-1.11
LOC_Os08g27540	expressed protein	3		-2.48
LOC_Os08g27580	expressed protein	3		-2.1
LOC_Os08g28030	retrotransposon protein, putative, Ty1-copia subclass	3		-1.16
LOC_Os08g28670	pathogenesis-related Bet v I family protein, putative	3		-2.56
LOC_Os08g28890	protein kinase family protein, putative	3		-1.84

LOC_Os08g29100	SAM domain family protein	3			-1.04
LOC_Os08g31219	ribosomal protein L27, putative	3			-1.43
LOC_Os08g31228	ribosomal protein L27, putative	3			-1.71
LOC_Os08g33750	myb-like DNA-binding domain containing protein	3			-0.76
LOC_Os08g34700	GDU1, putative	3			-0.82
LOC_Os08g36040	plant viral response family protein, putative	3			-1.11
LOC_Os08g42000	nuclear transport factor, putative	3			-0.83
LOC_Os08g44910	DNA binding protein, putative	3			-0.71
LOC_Os09g13940	AP2 domain containing protein	3			-1.44
LOC_Os09g14019	expressed protein	3			-1.05
LOC_Os09g14410	expressed protein	3			-1.6
LOC_Os09g14670	phosphoenolpyruvate carboxylase, putative	3			-1.38
LOC_Os09g15820	aldose 1-epimerase, putative	3			-0.95
LOC_Os09g15850	Leucine Rich Repeat family protein	3			-1.03
LOC_Os09g16760	ankyrin repeat family protein, putative	3			-0.75
LOC_Os09g16780	hypothetical protein	3			-1.79
LOC_Os09g20350	ethylene-responsive transcription factor, putative	3	-0.88	-1.15	-1.6
LOC_Os09g24190	hypothetical protein	3			-0.73
LOC_Os09g24620	expressed protein	3	-0.79		-1.48
LOC_Os09g26370	DUF581 domain containing protein	3			-1.42
LOC_Os09g28370	retrotransposon protein, putative, unclassified	3			-1.26
LOC_Os09g28510	EF hand family protein, putative	3			-0.71
LOC_Os09g29480	2-aminoethanethiol dioxygenase, putative	3			-0.91
LOC_Os09g36090	plus-3 domain containing protein	3		-1.02	-1.08
LOC_Os10g02070	peroxidase precursor, putative	3			-0.88
LOC_Os10g02880	O-methyltransferase, putative	3	1.01		-1.28
LOC_Os10g09620	OsWAK108 - OsWAK receptor-like protein kinase	3			-0.75
LOC_Os10g12500	integral membrane protein DUF6 containing protein	3		-1.03	-1.3
LOC_Os10g14920	integral membrane protein DUF6 containing protein	3			-0.75
LOC_Os10g15310	expressed protein	3			-0.73
LOC_Os10g22050	expressed protein	3			-0.86
LOC_Os10g30450	heavy-metal-associated domain-containing protein, putative	3	-1.16		-0.9
LOC_Os10g32348	PsbP, putative	3			-0.77
LOC_Os10g32400	PsbP, putative	3			-0.7
LOC_Os10g37290	DEFL6 - Defensin and Defensin-like DEFL family	3			-1.09
LOC_Os10g39980	expressed protein	3			-0.95
LOC_Os11g01380	clathrin heavy chain, putative	3			-1.16
LOC_Os11g02100	peroxidase precursor, putative	3			-1.01
LOC_Os11g04954	DNA repair protein Rad51, putative	3			-1.56
LOC_Os11g05190	phytosulfokines precursor, putative	3			-0.82
LOC_Os11g07600	ABC-2 type transporter domain containing protein	3			-0.84
LOC_Os11g08940	RNA polymerases N 8 kDa subunit, putative	3			-2.23
LOC_Os11g09310	zinc finger CCHC domain-containing protein 10, putative	3			-0.86
LOC_Os11g09979	expressed protein	3			-1.27
LOC_Os11g10510	dehydrogenase, putative	3			-1.05
LOC_Os11g10540	expressed protein	3			-0.72
LOC_Os11g14050	leucine-rich repeat family protein, putative	3			-0.72
LOC_Os11g17480	retrotransposon protein, putative, Ty1-copia subclass	3	-0.83		-1.72
LOC_Os11g23930	expressed protein	3			-1.07
LOC_Os11g24140	plastocyanin-like domain containing protein, putative	3			-1.74
LOC_Os11g25700	tropinone reductase, putative	3	-0.71		-0.86
LOC_Os11g30500	HVA22, putative	3			-0.7
LOC_Os11g34110	heparan-alpha-glucosaminide N-acetyltransferase, putative	3			-0.73
LOC_Os11g36070	expressed protein	3			-1.59

LOC_Os11g37040	expressed protein	3			-1,00
LOC_Os11g37060	OsFBDUF52 - F-box and DUF domain containing protein	3			-0.86
LOC_Os11g37490	expressed protein	3			-1.79
LOC_Os11g37759	stripe rust resistance protein Yr10, putative	3			-3.1
LOC_Os11g37860	stripe rust resistance protein Yr10, putative	3			-1.12
LOC_Os11g37950	WIP3 - Wound-induced protein precursor	3	1.16		-0.78
LOC_Os11g37970	WIP5 - Wound-induced protein precursor	3			-0.82
LOC_Os11g38140	OsFBDUF58 - F-box and DUF domain containing protein	3			-0.82
LOC_Os11g38170	notchless protein, putative	3			-1.49
LOC_Os11g38330	ZOS11-06 - C2H2 zinc finger protein	3			-0.94
LOC_Os11g38500	OsFBDUF62 - F-box and DUF domain containing protein	3			-0.78
LOC_Os11g38520	expressed protein	3			-1.08
LOC_Os11g38790	expressed protein	3			-1.92
LOC_Os11g39190	NB-ARC domain containing protein, putative	3			-1.25
LOC_Os11g39209	expressed protein	3			-0.81
LOC_Os11g39254	hypothetical protein	3			-1.06
LOC_Os11g39280	disease resistance protein, putative	3			-1,00
LOC_Os11g39650	WD domain, G-beta repeat domain containing protein	3			-1,00
LOC_Os11g40140	peptidase, T1 family, putative	3			-1.7
LOC_Os11g40410	expressed protein	3			-1.98
LOC_Os11g41034	expressed protein	3			-1.94
LOC_Os11g41560	OsFBX427 - F-box domain containing protein	3			-0.92
LOC_Os11g41650	adenylyl-sulfate kinase, putative	3			-0.92
LOC_Os11g42790	transporter, monovalent cation:proton antiporter-2 family, putative	3			-0.97
LOC_Os11g43420	LZ-NBS-LRR class RGA, putative	3			-1.63
LOC_Os11g44420	expressed protein	3			-0.84
LOC_Os11g44430	protein kinase, putative	3			-2.75
LOC_Os11g44630	calmodulin binding protein, putative	3		-0.89	-1.02
LOC_Os11g44800	expressed protein	3			-0.91
LOC_Os11g45130	pollen signalling protein with adenylyl cyclase activity, putative	3		-0.84	-2.06
LOC_Os11g45280	protein kinase family protein, putative	3			-0.74
LOC_Os11g45990	von Willebrand factor type A domain containing protein, putative	3	1.5		-1.5
LOC_Os11g46860	wall-associated receptor kinase-like 4 precursor, putative	3		-0.98	-1.87
LOC_Os11g47140	OsWAK123 - OsWAK receptor-like protein kinase	3			-2.84
LOC_Os11g47460	MYB family transcription factor, putative	3			-0.71
LOC_Os11g47830	RNA recognition motif containing protein	3			-1.16
LOC_Os11g48030	skp1 family, tetramerisation domain containing protein	3			-1.25
LOC_Os12g02060	peroxidase precursor, putative	3			-0.9
LOC_Os12g05690	expressed protein	3			-0.89
LOC_Os12g09300	amino acid transporter, putative	3			-0.9
LOC_Os12g09540	phosphoribosylamine--glycine ligase, putative	3			-0.71
LOC_Os12g10570	ATP synthase subunit beta, putative	3	-0.72		-0.8
LOC_Os12g10580	ribulose bisphosphate carboxylase large chain precursor, putative	3		-0.79	-0.89
LOC_Os12g30610	expressed protein	3			-1.24
LOC_Os12g37970	MYB family transcription factor, putative	3			-0.83
LOC_Os12g38660	hypothetical protein	3			-1.62
LOC_Os12g38720	expressed protein	3			-1.55
LOC_Os12g41930	SRP40, C-terminal domain containing protein	3			-0.84
LOC_Os12g43660	receptor-like protein kinase HAIKU2 precursor, putative	3			-0.74
LOC_Os12g44360	sodium/hydrogen exchanger 7, putative	3			-1.1
