# Supplementary material for: Physiological Basis and Transcriptional Profiling of Three Salt-Tolerant Mutant Lines of Rice
Source: Front Plant Sci. 2016 Sep 28;7:1462. doi: 10.3389/fpls.2016.01462 (PMC5039197; doi:10.3389/fpls.2016.01462)
Supplement: Supplementary file 4 [file Table4.PDF]

Supplementary table S4.- Selection of differentially regulated genes classified in different categories according to the Gene Ontology classification: LPT family, lipid metabolism, oxidation-reduction process, secondary metabolic process, response to abiotic stimulus and defense response and generation of precursor metabolites and energy.

Locus	Putative Function	SaT58	SaS62	SaT20
**LTP family**				
LOC_Os02g44310	LTPL112 - Protease inhibitor/seed storage/LTP family protein precursor			0,93
LOC_Os03g01300	LTPL114 - Protease inhibitor/seed storage/LTP family protein precursor	1,29		
LOC_Os03g01320	LTPL116 - Protease inhibitor/seed storage/LTP family protein precursor	0,79		1,63
LOC_Os04g46820	LTPL121 - Protease inhibitor/seed storage/LTP family protein precursor	-0,70	-1,76	
LOC_Os10g40430	LTPL139 - Protease inhibitor/seed storage/LTP family protein precursor		-0,72	
LOC_Os10g40480	LTPL143 - Protease inhibitor/seed storage/LTP family protein precursor		-0,84	
LOC_Os10g40510	LTPL144 - Protease inhibitor/seed storage/LTP family protein precursor	0,92		0,93
LOC_Os10g40520	LTPL145 - Protease inhibitor/seed storage/LTP family protein precursor		-0,82	
LOC_Os01g49640	LTPL149 - Protease inhibitor/seed storage/LTP family protein precursor			-1,16
LOC_Os07g18990	LTPL4 - Protease inhibitor/seed storage/LTP family protein precursor			-1,57
LOC_Os07g19000	LTPL41 - Protease inhibitor/seed storage/LTP family protein precursor			2,53
LOC_Os07g18750	LTPL42 - Protease inhibitor/seed storage/LTP family protein precursor			0,72
LOC_Os03g46150	LTPL72 - Protease inhibitor/seed storage/LTP family protein precursor			0,73
LOC_Os03g57970	LTPL73 - Protease inhibitor/seed storage/LTP family protein precursor	1,93		0,77
LOC_Os03g57990	LTPL74 - Protease inhibitor/seed storage/LTP family protein precursor	0,88		
LOC_Os07g07860	LTPL76 - Protease inhibitor/seed storage/LTP family protein precursor		-1,00	
LOC_Os07g09970	LTPL84 - Protease inhibitor/seed storage/LTP family protein precursor	0,86		0,97
LOC_Os03g57980	LTPL99 - Protease inhibitor/seed storage/LTP family protein precursor	0,99		0,72
**lipid metabolic process**				
LOC_Os01g07760	phospholipase D, putative		-0,84	
LOC_Os05g49840	phospholipase, putative		-0,76	
LOC_Os01g27230	12-oxophytodienoate reductase, putative	0,81		0,97
LOC_Os01g01710	1-deoxy-D-xylulose 5-phosphate reductoisomerase, chloroplast precursor, putative	0,85		
LOC_Os03g12030	3-ketoacyl-CoA synthase, putative	0,75		
LOC_Os03g14170	3-ketoacyl-CoA synthase, putative	0,85		
LOC_Os02g57260	3-ketoacyl-CoA thiolase, peroxisomal precursor, putative		-1,57	
LOC_Os01g63260	3-oxo-5-alpha-steroid 4-dehydrogenase, putative			-1,24
LOC_Os03g52180	4-hydroxy-3-methylbut-2-enyl diphosphate reductase, putative			-0,70
LOC_Os01g65830	acyl-desaturase, chloroplast precursor, putative			-0,87
LOC_Os07g18120	aldehyde oxidase, putative	0,92		
LOC_Os07g18162	aldehyde oxidase, putative			-0,84
LOC_Os12g26290	alpha-DOX2, putative	0,86		
LOC_Os07g17970	AMP-binding domain containing protein			-1,52
LOC_Os01g38580	beta,beta-carotene 9,10-dioxygenase, putative	-0,76		
LOC_Os11g16960	carnitine racemase like protein, putative		-0,71	
LOC_Os01g63540	cytochrome P450, putative			1,82
LOC_Os02g44654	cytochrome P450, putative	0,73		
LOC_Os05g11130	cytochrome P450, putative			-0,75
LOC_Os10g34480	cytochrome P450, putative	1,28		0,98
LOC_Os02g01760	diphosphomevalonate decarboxylase family protein		-1,03	
LOC_Os05g41120	endoplasmic reticulum-Golgi intermediate compartment protein 3, putative		-0,81	
LOC_Os02g36210	ent-kaurene synthase, chloroplast precursor, putative	1,04	0,74	1,07
LOC_Os04g10060	ent-kaurene synthase, chloroplast precursor, putative	0,94		0,72
LOC_Os01g70860	esterase, putative		0,71	
LOC_Os03g15460	expressed protein			1,10
LOC_Os07g23410	fatty acid desaturase, putative	0,71		1,50

LOC_Os07g23430	fatty acid desaturase, putative	1,13		
LOC_Os01g11570	GDSL-like lipase/acylhydrolase, putative			-1,52
LOC_Os01g11620	GDSL-like lipase/acylhydrolase, putative	1,00		
LOC_Os02g50000	GDSL-like lipase/acylhydrolase, putative			1,25
LOC_Os03g25030	GDSL-like lipase/acylhydrolase, putative	-0,75	-1,54	-0,87
LOC_Os05g06720	GDSL-like lipase/acylhydrolase, putative	0,96		0,81
LOC_Os06g14630	GDSL-like lipase/acylhydrolase, putative			0,71
LOC_Os07g44780	GDSL-like lipase/acylhydrolase, putative		-0,89	
LOC_Os07g47210	GDSL-like lipase/acylhydrolase, putative	1,17		0,80
LOC_Os11g31940	GDSL-like lipase/acylhydrolase, putative	0,77		
LOC_Os01g55240	gibberellin 2-beta-dioxygenase, putative			-0,90
LOC_Os01g06600	glutaryl-CoA dehydrogenase, mitochondrial precursor, putative			2,58
LOC_Os05g38350	glycerol-3-phosphate acyltransferase 8, putative	0,77		0,79
LOC_Os02g02340	glycerol-3-phosphate acyltransferase, putative	0,81		
LOC_Os02g39160	hydroxymethylbutenyl 4-diphosphate synthase, putative		-0,72	
LOC_Os01g13680	hypothetical protein	-0,95		
LOC_Os03g13030	lecithin cholesterol acyltransferase, putative			0,83
LOC_Os01g46370	lipase class 3 family protein, putative		-1,14	
LOC_Os03g52860	lipoxygenase, putative	1,38		
LOC_Os08g20200	male sterility protein, putative	-0,85		-0,72
LOC_Os09g39410	male sterility protein, putative	0,74		1,05
LOC_Os03g50130	microsomal glutathione S-transferase 3, putative		0,76	1,27
LOC_Os06g45860	molybdenum cofactor sulfurase, putative			-0,84
LOC_Os07g25150	myb-related protein 306, putative		-0,72	-1,39
LOC_Os02g43090	myristoyl-acyl carrier protein thioesterase, chloroplast precursor, putative		-0,78	
LOC_Os04g40730	oxidoreductase, short chain dehydrogenase/reductase family, putative	0,88		
LOC_Os08g28880	patatin, putative	0,72		1,04
LOC_Os08g37180	patatin, putative	1,29		0,75
LOC_Os08g37210	patatin, putative			1,07
LOC_Os08g37250	patatin, putative	1,08		1,46
LOC_Os04g59630	prenylcysteine oxidase 1 precursor, putative	-0,78	-0,98	
LOC_Os07g41460	sulfotransferase domain containing protein			1,13
LOC_Os08g40380	sulfotransferase domain containing protein	1,04	-0,95	0,80
LOC_Os11g30810	sulfotransferase domain containing protein	1,05		0,85
LOC_Os02g36140	terpene synthase, putative	1,61	1,06	1,00
LOC_Os12g30824	terpene synthase, putative			0,97
LOC_Os11g31090	transferase family protein, putative	0,98		
LOC_Os07g09190	transketolase, putative	0,70		

oxidation-reduction process

LOC_Os05g38150	amino acid kinase, putative (P5CS)		-1,39	
LOC_Os01g27230	12-oxophytodienoate reductase, putative	0,81		0,97
LOC_Os03g48430	1-aminocyclopropane-1-carboxylate oxidase homolog 4, putative			-0,85
LOC_Os11g08380	1-aminocyclopropane-1-carboxylate oxidase, putative			0,76
LOC_Os01g01710	1-deoxy-D-xylulose 5-phosphate reductoisomerase, chloroplast precursor, putative	0,85		
LOC_Os01g09030	2-aminoethanethiol dioxygenase, putative			-0,86
LOC_Os09g29480	2-aminoethanethiol dioxygenase, putative			-0,98
LOC_Os04g53830	3-beta hydroxysteroid dehydrogenase/isomerase family protein, putative			-0,94
LOC_Os03g52180	4-hydroxy-3-methylbut-2-enyl diphosphate reductase, putative			-0,76
LOC_Os01g65830	acyl-desaturase, chloroplast precursor, putative			-0,87
LOC_Os07g22950	adenylate kinase isoenzyme 6, putative			-0,81
LOC_Os08g32870	aldehyde dehydrogenase, putative		-1,00	
LOC_Os07g18120	aldehyde oxidase, putative	0,92		
LOC_Os07g18162	aldehyde oxidase, putative			-0,84
LOC_Os12g26290	alpha-DOX2, putative	0,86		

LOC_Os08g06170	berberine and berberine like domain containing protein			-1,16
LOC_Os01g38580	beta,beta-carotene 9,1-dioxygenase, putative	-0,76		
LOC_Os02g02400	catalase isozyme A, putative	1,44		
LOC_Os02g56720	cinnamoyl CoA reductase, putative		-0,74	
LOC_Os06g23114	copper methylamine oxidase precursor, putative			0,99
LOC_Os01g66180	cytochrome c, putative			-0,83
LOC_Os12g16720	cytochrome P450 71A1, putative	0,89	0,87	0,94
LOC_Os01g43710	cytochrome P450 72A1, putative		0,84	1,46
LOC_Os07g44140	cytochrome P450 72A1, putative			1,64
LOC_Os06g24180	cytochrome P450 84A1, putative	1,24		0,78
LOC_Os04g01140	cytochrome P450 93A2, putative	0,74		
LOC_Os07g33480	cytochrome P450 domain containing protein			-2,25
LOC_Os11g04290	cytochrome P450, putative	0,85	1,31	1,90
LOC_Os10g16974	cytochrome P450, putative			0,72
LOC_Os10g34480	cytochrome P450, putative	1,28		0,98
LOC_Os10g38090	cytochrome P450, putative		-0,84	
LOC_Os01g63540	cytochrome P450, putative			1,82
LOC_Os10g08319	cytochrome P450, putative	1,19	-0,87	
LOC_Os10g09110	cytochrome P450, putative			1,38
LOC_Os02g30100	cytochrome P450, putative			0,82
LOC_Os02g32770	cytochrome P450, putative		-0,86	
LOC_Os02g36110	cytochrome P450, putative	1,25	0,87	1,39
LOC_Os02g36190	cytochrome P450, putative	1,39	1,23	1,14
LOC_Os02g36280	cytochrome P450, putative			0,82
LOC_Os02g44654	cytochrome P450, putative	0,73		
LOC_Os02g09220	cytochrome P450, putative		-0,88	
LOC_Os03g37290	cytochrome P450, putative	1,39	1,34	1,21
LOC_Os03g55240	cytochrome P450, putative		1,56	2,38
LOC_Os04g10160	cytochrome P450, putative	1,25	0,76	0,92
LOC_Os05g11130	cytochrome P450, putative			-0,75
LOC_Os06g41070	cytochrome P450, putative			1,42
LOC_Os06g43410	cytochrome P450, putative		-1,61	
LOC_Os07g11739	cytochrome P450, putative	0,72	0,82	1,52
LOC_Os07g33560	cytochrome P450, putative		-0,85	
LOC_Os08g43390	cytochrome P450, putative			1,40
LOC_Os08g43440	cytochrome P450, putative	0,75		
LOC_Os09g27510	cytochrome P450, putative		0,83	1,00
LOC_Os09g35940	cytochrome P450, putative			1,26
LOC_Os08g35860	cytokinin dehydrogenase precursor, putative	-0,85		
LOC_Os11g10480	dehydrogenase, putative		-1,17	
LOC_Os11g10510	dehydrogenase, putative			-1,48
LOC_Os03g09020	dehydrogenase, putative	1,23		
LOC_Os04g15920	dehydrogenase, putative	0,96		
LOC_Os04g29030	dehydrogenase, putative			1,33
LOC_Os09g32290	FAD dependent oxidoreductase domain containing protein			0,75
LOC_Os08g02230	FAD-binding and arabino-lactone oxidase domains containing protein, putative			0,95
LOC_Os07g23410	fatty acid desaturase, putative	0,71		1,50
LOC_Os07g23430	fatty acid desaturase, putative	1,13		
LOC_Os07g02140	flavin-containing monooxygenase family protein, putative	-0,88		
LOC_Os06g07932	flavonol synthase/flavanone 3-hydroxylase, putative	0,84		0,77
LOC_Os03g42130	gibberellin 2 oxidase 2, putative		0,84	
LOC_Os01g55240	gibberellin 2-beta-dioxygenase, putative			-0,90
LOC_Os07g22350	glucose-6-phosphate 1-dehydrogenase, chloroplast precursor, putative			-1,43
LOC_Os04g45970	glutamate dehydrogenase protein, putative			0,73
LOC_Os01g48960	glutamate synthase, chloroplast precursor, putative		-1,33	-1,49
LOC_Os01g06600	glutaryl-CoA dehydrogenase, mitochondrial precursor, putative			2,58

LOC_Os02g56850	glutathione reductase, putative		-1,28	
LOC_Os01g58740	glycerol-3-phosphate dehydrogenase, putative		-1,41	
LOC_Os01g51410	glycine dehydrogenase, putative		-1,72	
LOC_Os02g39160	hydroxymethylbutenyl 4-diphosphate synthase, putative		-0,72	
LOC_Os03g13560	hydroxyproline-rich glycoprotein family protein, putative		-0,74	
LOC_Os05g38390	laccase precursor protein, putative			0,76
LOC_Os11g48060	laccase-22 precursor, putative		0,78	
LOC_Os01g61380	lactate/malate dehydrogenase, putative			-1,28
LOC_Os04g46560	lactate/malate dehydrogenase, putative			1,44
LOC_Os05g49880	lactate/malate dehydrogenase, putative			-0,79
LOC_Os06g37080	L-ascorbate oxidase precursor, putative			-0,82
LOC_Os06g43670	Leucine Rich Repeat family protein		-0,77	
LOC_Os04g53850	leucoanthocyanidin reductase, putative			-2,76
LOC_Os03g52860	lipoxygenase, putative	1,38		
LOC_Os02g47790	monodehydroascorbate reductase, putative			0,76
LOC_Os08g44340	monodehydroascorbate reductase, putative		-0,74	
LOC_Os01g03630	multicopper oxidase domain containing protein			2,47
LOC_Os01g03640	multicopper oxidase domain containing protein	0,89		
LOC_Os01g59930	NADH-cytochrome b5 reductase, putative			-1,69
LOC_Os11g14910	NADP-dependent oxidoreductase, putative			0,80
LOC_Os08g04460	NADPH-dependent FMN reductase domain containing protein	0,87		
LOC_Os02g53130	nitrate reductase, putative		0,88	
LOC_Os07g32570	OsAPRL1 adenosine 5'-phosphosulfate reductase-like OsAPRL1		1,34	
LOC_Os05g50090	oxidoreductase, 2OG-FeII oxygenase domain containing protein, putative			2,83
LOC_Os01g62870	oxidoreductase, aldo/keto reductase family protein, putative			0,96
LOC_Os04g27060	oxidoreductase, aldo/keto reductase family protein, putative	2,60	2,95	3,61
LOC_Os04g08550	oxidoreductase, aldo/keto reductase family protein, putative	0,75		1,69
LOC_Os04g40730	oxidoreductase, short chain dehydrogenase/reductase family, putative	0,88		
LOC_Os11g02100	peroxidase precursor, putative			-1,92
LOC_Os10g02070	peroxidase precursor, putative			-0,88
LOC_Os01g36240	peroxidase precursor, putative		0,76	
LOC_Os03g13210	peroxidase precursor, putative			-0,78
LOC_Os03g22020	peroxidase precursor, putative	0,71		0,76
LOC_Os03g25330	peroxidase precursor, putative		-0,86	
LOC_Os03g02939	peroxidase precursor, putative		-0,74	-0,94
LOC_Os04g55740	peroxidase precursor, putative			-0,84
LOC_Os05g04410	peroxidase precursor, putative			0,82
LOC_Os05g04450	peroxidase precursor, putative			0,88
LOC_Os05g04500	peroxidase precursor, putative	1,27		
LOC_Os05g06970	peroxidase precursor, putative			0,74
LOC_Os06g16350	peroxidase precursor, putative	0,93		
LOC_Os06g35520	peroxidase precursor, putative			1,26
LOC_Os07g01370	peroxidase precursor, putative	1,13		0,93
LOC_Os07g48010	peroxidase precursor, putative	1,45		1,80
LOC_Os07g48020	peroxidase precursor, putative	1,22		1,26
LOC_Os07g48030	peroxidase precursor, putative	1,26		
LOC_Os07g48060	peroxidase precursor, putative			0,85
LOC_Os08g02110	peroxidase precursor, putative			-1,40
LOC_Os10g17790	remorin C-terminal domain containing protein, putative			1,59
LOC_Os06g35560	reticuline oxidase-like protein precursor, putative			-0,89
LOC_Os02g30310	ThiF family domain containing protein, putative			-0,80
LOC_Os11g25700	tropinone reductase, putative	-0,72		-0,86
LOC_Os01g64520	uricase, putative	-1,31	-0,78	-1,19

<u>Secondary metabolic process</u>

LOC_Os01g01710	1-deoxy-D-xylulose 5-phosphate reductoisomerase, chloroplast precursor,	0,85		

	putative			
LOC_Os04g53830	3-beta hydroxysteroid dehydrogenase/isomerase family protein, putative			-0,93
LOC_Os07g18162	aldehyde oxidase, putative			-0,84
LOC_Os08g38910	caffeoyl-CoA O-methyltransferase, putative	1,35		0,72
LOC_Os08g38920	caffeoyl-CoA O-methyltransferase, putative	1,06		
LOC_Os07g34260	chalcone and stilbene synthases, putative	1,03		
LOC_Os11g32650	chalcone synthase, putative	0,71		1,03
LOC_Os02g56720	cinnamoyl CoA reductase, putative		-0,74	
LOC_Os01g43710	cytochrome P450 72A1, putative		0,84	1,46
LOC_Os01g63540	cytochrome P450, putative			1,82
LOC_Os02g36110	cytochrome P450, putative	1,25	0,87	1,39
LOC_Os03g37290	cytochrome P450, putative	1,39	1,34	1,21
LOC_Os03g55240	cytochrome P450, putative		1,51	2,38
LOC_Os06g24180	cytochrome P450 84A1, putative	1,02		0,78
LOC_Os09g27510	cytochrome P450, putative		0,83	0,99
LOC_Os10g08319	cytochrome P450, putative	1,11	-0,87	
LOC_Os10g09110	cytochrome P450, putative			1,38
LOC_Os10g16974	cytochrome P450, putative			0,72
LOC_Os10g34480	cytochrome P450, putative	1,28		0,98
LOC_Os10g38090	cytochrome P450, putative		-0,84	
LOC_Os04g15920	dehydrogenase, putative	0,96		
LOC_Os07g44450	dirigent, putative			0,84
LOC_Os11g42550	dirigent, putative			0,75
LOC_Os05g03820	glutamate--cysteine ligase, chloroplast precursor, putative			-0,95
LOC_Os10g38340	glutathione S-transferase GSTU6, putative	1,44	1,73	2,77
LOC_Os10g38489	glutathione S-transferase GSTU6, putative	1,45	1,89	2,66
LOC_Os10g38600	glutathione S-transferase GSTU6, putative	1,26	1,42	2,37
LOC_Os10g38314	glutathione S-transferase, N-terminal domain containing protein		-1,69	-1,12
LOC_Os01g25100	glutathione S-transferase, putative			0,79
LOC_Os01g49710	glutathione S-transferase, putative		1,43	1,76
LOC_Os01g49720	glutathione S-transferase, putative	1,03	2,00	2,20
LOC_Os01g72140	glutathione S-transferase, putative			0,71
LOC_Os01g72150	glutathione S-transferase, putative		0,91	1,60
LOC_Os03g04240	glutathione S-transferase, putative	0,85		
LOC_Os03g39850	glutathione S-transferase, putative			0,99
LOC_Os03g57200	glutathione S-transferase, putative	1,17		1,51
LOC_Os09g20220	glutathione S-transferase, putative	0,85	2,42	2,76
LOC_Os10g38360	glutathione S-transferase, putative	0,97	1,10	2,27
LOC_Os10g38470	glutathione S-transferase, putative			1,01
LOC_Os10g38700	glutathione S-transferase, putative			0,70
LOC_Os10g38740	glutathione S-transferase, putative	0,78		0,92
LOC_Os05g38350	glycerol-3-phosphate acyltransferase 8, putative	0,77		0,79
LOC_Os02g39160	hydroxymethylbutenyl 4-diphosphate synthase, putative		-0,72	
LOC_Os05g38390	laccase precursor protein, putative			0,76
LOC_Os11g48060	laccase-22 precursor, putative		0,78	
LOC_Os04g53850	leucoanthocyanidin reductase, putative			-2,76
LOC_Os08g20200	male sterility protein, putative	-0,85		-0,72
LOC_Os09g39410	male sterility protein, putative	0,74		1,05
LOC_Os12g16220	nmrA-like family domain containing protein		-0,86	
LOC_Os01g42380	pleiotropic drug resistance protein, putative	0,70		0,71
LOC_Os07g43670	ribonuclease T2 family domain containing protein		-0,96	
LOC_Os04g10000	sex determination protein tasselseed-2, putative	0,95	0,89	
LOC_Os06g08640	transferase family protein, putative	1,68	0,71	
LOC_Os11g31090	transferase family protein, putative	0,98		
LOC_Os06g18140	UDP-glucoronosyl and UDP-glucosyl transferase domain containing protein		0,94	1,33

<u>response to abiotic stimulus</u>

LOC_Os03g46060	thaumatin family domain containing protein			1,17
LOC_Os12g43390	thaumatin, putative	0,99		
LOC_Os03g46070	thaumatin, putative	1,23		
LOC_Os12g43490	thaumatin, putative			0,85
LOC_Os03g12030	3-ketoacyl-CoA synthase, putative	0,76		
LOC_Os03g14170	3-ketoacyl-CoA synthase, putative	0,85		
LOC_Os11g37900	3-ketoacyl-CoA synthase, putative	0,86		
LOC_Os01g63260	3-oxo-5-alpha-steroid 4-dehydrogenase, putative			-1,24
LOC_Os01g50100	ABC transporter, ATP-binding protein, putative	0,84	0,76	1,69
LOC_Os06g19960	aconitate hydratase protein, putative		-1,00	
LOC_Os10g36650	actin, putative	0,73		
LOC_Os08g06550	acyl CoA binding protein, putative	0,86		0,85
LOC_Os08g32870	aldehyde dehydrogenase, putative		-1,00	
LOC_Os01g63270	alpha-glucan phosphorylast isozyme, putative			0,72
LOC_Os05g38150	amino acid kinase, putative		-1,39	
LOC_Os12g09300	amino acid transporter, putative			-0,93
LOC_Os07g22390	ankyrin repeat domain containing protein			-2,61
LOC_Os06g47590	AP2 domain containing protein	-1,37		
LOC_Os04g44060	aquaporin protein, putative			-0,85
LOC_Os07g26630	aquaporin protein, putative			2,79
LOC_Os03g47830	argonaute, putative		-1,66	
LOC_Os12g10570	ATP synthase subunit beta, putative	-0,72		-0,83
LOC_Os06g48950	auxin response factor 19, putative		-0,79	
LOC_Os08g09830	BTB and MATH domain containing protein, putative			1,21
LOC_Os02g38120	BTBN3 - Bric-a-Brac, Tramtrack, Broad Complex BTB domain with non-phototropic hypocotyl 3 NPH3 domain	0,74		
LOC_Os01g66890	BTBZ1 - Bric-a-Brac, Tramtrack, and Broad Complex BTB domain with TAZ zinc finger and Calmodulin-binding domains			-0,72
LOC_Os02g18690	BURP domain containing protein			-1,59
LOC_Os06g19800	BURP domain containing protein	1,79		1,29
LOC_Os06g50600	bZIP transcription factor domain containing protein			-1,11
LOC_Os01g64000	bZIP transcription factor, putative	0,73		
LOC_Os01g51420	calcineurin B, putative	-0,99	-0,89	
LOC_Os10g41510	calcineurin B, putative		-0,87	
LOC_Os03g33570	calcineurin B-like protein 8, putative			-0,87
LOC_Os12g03810	CAMK_KIN1/SNF1/Nim1_like.37 - CAMK includes calcium/calmodulin depedent protein kinases			0,95
LOC_Os11g03970	CAMK_KIN1/SNF1/Nim1_like.5 - CAMK includes calcium/calmodulin depedent protein kinases			1,21
LOC_Os02g02400	catalase isozyme A, putative	1,44		
LOC_Os03g52690	CBS domain containing membrane protein, putative			-0,75
LOC_Os07g34260	chalcone and stilbene synthases, putative	1,29		
LOC_Os11g32650	chalcone synthase, putative	0,78		1,28
LOC_Os11g02440	chalcone--flavonone isomerase, putative	0,72		
LOC_Os02g08490	chaperone protein clpB 1, putative			0,97
LOC_Os02g56720	cinnamoyl CoA reductase, putative		-0,74	
LOC_Os12g05880	Cupin domain containing protein			0,84
LOC_Os03g16960	cysteine-rich repeat secretory protein 55 precursor, putative		0,83	
LOC_Os02g54880	cytochrome c oxidase subunit, putative	-0,76		-1,18
LOC_Os12g16720	cytochrome P450 71A1, putative	0,89	0,87	0,94
LOC_Os06g24180	cytochrome P450 84A1, putative	1,24		0,78
LOC_Os10g16974	cytochrome P450, putative			0,72
LOC_Os11g10480	dehydrogenase, putative		-1,17	
LOC_Os11g10510	dehydrogenase, putative			-1,48
LOC_Os06g04510	enolase, putative	1,18	2,25	2,58
LOC_Os09g20350	ethylene-responsive transcription factor, putative	-0,88	-1,15	-1,60

LOC_Os03g08580	expressed protein			0,78
LOC_Os05g12630	expressed protein	1,68		1,20
LOC_Os05g33380	fructose-bisphospate aldolase isozyme, putative		0,73	
LOC_Os01g55240	gibberellin 2-beta-dioxygenase, putative			-0,90
LOC_Os04g45970	glutamate dehydrogenase protein, putative			0,73
LOC_Os07g33790	glutamate receptor 3.4 precursor, putative			-1,88
LOC_Os05g03820	glutamate--cysteine ligase, chloroplast precursor, putative			-0,95
LOC_Os10g38340	glutathione S-transferase GSTU6, putative	1,44	1,73	2,77
LOC_Os10g38489	glutathione S-transferase GSTU6, putative	1,45	1,89	2,66
LOC_Os10g38314	glutathione S-transferase, N-terminal domain containing protein		-1,69	-1,12
LOC_Os03g57200	glutathione S-transferase, putative	1,17		1,51
LOC_Os10g38700	glutathione S-transferase, putative			0,75
LOC_Os10g38740	glutathione S-transferase, putative	0,78		0,92
LOC_Os01g58740	glycerol-3-phosphate dehydrogenase, putative		-1,41	
LOC_Os01g49320	glycosyl hydrolase, putative	0,77		
LOC_Os01g64100	glycosyl hydrolase, putative			1,64
LOC_Os04g27980	glycosyl hydrolase, putative		-1,12	
LOC_Os05g15770	glycosyl hydrolase, putative			1,11
LOC_Os06g25010	glycosyl hydrolase, putative			0,75
LOC_Os01g51570	glycosyl hydrolases family 17, putative	1,56		
LOC_Os05g31140	glycosyl hydrolases family 17, putative			1,24
LOC_Os03g20120	glycosyl transferase 8 domain containing protein, putative	0,87		
LOC_Os06g43620	haemolysin-III, putative	0,92		
LOC_Os02g52150	heat shock 22 kDa protein, mitochondrial precursor, putative		1,16	1,94
LOC_Os06g11610	heat shock 22 kDa protein, mitochondrial precursor, putative	0,85	1,43	1,80
LOC_Os10g36370	heat shock protein DnaJ, putative		-1,23	
LOC_Os10g42439	heat shock protein DnaJ, putative		-0,86	
LOC_Os07g38910	histidine kinase, putative		-1,65	
LOC_Os03g06170	hsp20/alpha crystallin family protein, putative			1,49
LOC_Os04g36750	hsp20/alpha crystallin family protein, putative			-1,48
LOC_Os07g33350	hsp20/alpha crystallin family protein, putative			1,12
LOC_Os10g07210	hsp20/alpha crystallin family protein, putative			1,74
LOC_Os10g30180	hsp20/alpha crystallin family protein, putative			0,96
LOC_Os09g27730	HVA22, putative			1,49
LOC_Os11g30500	HVA22, putative			-0,72
LOC_Os02g55890	inorganic H+ pyrophosphatase, putative			-1,32
LOC_Os01g61380	lactate/malate dehydrogenase, putative			-1,28
LOC_Os05g49880	lactate/malate dehydrogenase, putative			-0,79
LOC_Os08g23870	late embryogenesis abundant group 1, putative			-0,83
LOC_Os01g21250	late embryogenesis abundant protein, putative	0,76		
LOC_Os01g46370	lipase class 3 family protein, putative		-1,14	
LOC_Os11g04104	major facilitator superfamily antiporter, putative			0,71
LOC_Os08g20200	male sterility protein, putative	-0,85		-0,72
LOC_Os09g39410	male sterility protein, putative	0,74		1,53
LOC_Os08g13060	MBTB22 - Bric-a-Brac, Tramtrack, Broad Complex BTB domain with Meprin and TRAF Homology MATH domain			-0,85
LOC_Os02g37000	mitochondrial prohibitin complex protein 1, putative		0,78	0,85
LOC_Os06g45860	molybdenum cofactor sulfurase, putative			-0,84
LOC_Os08g44340	monodehydroascorbate reductase, putative		-0,74	
LOC_Os01g03630	multicopper oxidase domain containing protein			2,47
LOC_Os01g03640	multicopper oxidase domain containing protein	0,89		
LOC_Os02g53130	nitrate reductase, putative		0,88	
LOC_Os07g48780	OsCam1-2 - Calmodulin		-1,66	
LOC_Os06g11330	OsMADS55 - MADS-box family gene with MIKCc type-box	0,96		
LOC_Os07g44180	OsRCI2-10 - Hydrophobic protein LTI6A			0,79
LOC_Os05g45950	outer mitochondrial membrane porin, putative			0,74

LOC_Os04g08550	oxidoreductase, aldo/keto reductase family protein, putative	0,75		1,69
LOC_Os05g41180	peptidase, T1 family, putative		0,82	1,14
LOC_Os01g38229	peptidyl-prolyl isomerase, putative	0,77		
LOC_Os01g22336	peroxidase precursor, putative			1,13
LOC_Os03g02939	peroxidase precursor, putative		-0,74	-0,94
LOC_Os05g04500	peroxidase precursor, putative	1,27		
LOC_Os05g06970	peroxidase precursor, putative			0,74
LOC_Os07g01370	peroxidase precursor, putative	1,13		0,93
LOC_Os07g03418	phototropin, putative		0,85	1,34
LOC_Os05g39350	PMR5, putative	0,73		
LOC_Os01g45990	potassium channel AKT1, putative	0,78		
LOC_Os04g37904	protein phosphatase 2C, putative		-1,58	
LOC_Os06g04070	pyridoxal-dependent decarboxylase protein, putative	-0,77		-0,86
LOC_Os10g42710	RCD1, putative		-1,92	
LOC_Os06g47580	REV1, putative		-0,79	
LOC_Os01g10940	S-adenosylmethionine synthetase 2, putative	-0,82		
LOC_Os04g49920	sensitivity to red light reduced protein 1, putative		-0,77	
LOC_Os03g10050	serine acetyltransferase protein, putative			1,37
LOC_Os12g44360	sodium/hydrogen exchanger 7, putative			-1,12
LOC_Os01g63210	SOUL heme-binding protein, putative	1,51	1,70	2,16
LOC_Os07g41460	sulfotransferase domain containing protein			1,13
LOC_Os11g30810	sulfotransferase domain containing protein	1,59		0,85
LOC_Os03g01910	transcription factor BTF3, putative		-0,74	
LOC_Os08g07970	transcription factor, putative			-0,71
LOC_Os01g62420	triosephosphate isomerase, cytosolic, putative		0,92	0,96
LOC_Os03g11970	tubulin/FtsZ domain containing protein, putative	-1,29	-0,76	
LOC_Os03g51600	tubulin/FtsZ domain containing protein, putative			0,87
LOC_Os11g14220	tubulin/FtsZ domain containing protein, putative	-0,87		
LOC_Os04g12960	UDP-glucoronosyl/UDP-glucosyl transferase, putative		0,80	1,13
LOC_Os05g07810	universal stress protein domain containing protein, putative			-1,18
LOC_Os01g63900	WD domain, G-beta repeat domain containing protein			-1,84
LOC_Os11g37950	WIP3 - Wound-induced protein precursor	1,17		-0,78
LOC_Os11g37970	WIP5 - Wound-induced protein precursor			-0,82
LOC_Os02g28074	XRN 5-3 exonuclease N-terminus family protein			-1,70
LOC_Os04g16970	zinc finger, C3HC4 type domain containing protein	0,76		
LOC_Os05g03810	trehalose phosphatase, putative		-1,00	
LOC_Os12g32130	trehalose phosphatase, putative		-1,02	
LOC_Os09g20990	trehalose-6-phosphate synthase, putative		-2,37	

defense response

LOC_Os10g39680	CHIT14 - Chitinase family protein precursor	0,89		
LOC_Os03g04060	CHIT16 - Chitinase family protein precursor	0,81		
LOC_Os05g33130	CHIT17 - Chitinase family protein precursor	0,73		0,99
LOC_Os01g56420	ctr copper transporter family protein, putative		-0,77	
LOC_Os04g28250	cysteine proteinase inhibitor precursor, putative			0,91
LOC_Os04g10160	cytochrome P45, putative	1,25	0,76	0,92
LOC_Os02g41904	DEF7 - Defensin and Defensin-like DEFL family		-0,96	
LOC_Os01g33810	disease resistance protein RPM1, putative		-0,78	
LOC_Os08g07774	disease resistance protein RPM1, putative		-1,33	
LOC_Os11g11950	disease resistance protein RPM1, putative			1,35
LOC_Os04g43340	disease resistance RPP13-like protein 1, putative		-0,71	
LOC_Os02g36210	ent-kaurene synthase, chloroplast precursor, putative	1,40	0,74	1,68
LOC_Os04g10060	ent-kaurene synthase, chloroplast precursor, putative	0,94		0,72
LOC_Os05g05060	expressed protein	-0,81		-0,75
LOC_Os09g14410	expressed protein			-1,61

LOC_Os06g43670	Leucine Rich Repeat family protein		-0,77	
LOC_Os09g15850	Leucine Rich Repeat family protein			-1,35
LOC_Os11g39290	Leucine Rich Repeat family protein			1,17
LOC_Os11g39320	LZ-NBS-LRR class, putative			0,78
LOC_Os01g05650	metallothionein, putative			-1,57
LOC_Os11g11940	MLA1, putative			1,29
LOC_Os08g07890	NB-ARC domain containing protein			-0,73
LOC_Os11g17014	NB-ARC domain containing protein	0,76		
LOC_Os11g39190	NB-ARC domain containing protein, putative			-1,25
LOC_Os11g12300	NBS-LRR disease resistance protein, putative		-0,82	
LOC_Os11g44970	NBS-LRR disease resistance protein, putative			0,98
LOC_Os04g53060	NBS-LRR disease resistance protein, putative			-1,20
LOC_Os11g12000	NBS-LRR disease resistance protein, putative			1,38
LOC_Os11g45970	NBS-LRR disease resistance protein, putative			1,22
LOC_Os07g33690	NBS-LRR type disease resistance protein Hom-F, putative			-1,35
LOC_Os08g28670	pathogenesis-related Bet v I family protein, putative			-2,56
LOC_Os12g36830	pathogenesis-related Bet v I family protein, putative	1,28		
LOC_Os12g36840	pathogenesis-related Bet v I family protein, putative	0,86		
LOC_Os12g36850	pathogenesis-related Bet v I family protein, putative	0,90		
LOC_Os12g36880	pathogenesis-related Bet v I family protein, putative	1,80		
LOC_Os12g36860	pathogenesis-related protein 1, putative	1,39		
LOC_Os11g45130	pollen signalling protein with adenylyl cyclase activity, putative		-0,84	-2,68
LOC_Os11g37759	stripe rust resistance protein Yr1, putative			-3,12
LOC_Os11g37860	stripe rust resistance protein Yr1, putative			-1,12
LOC_Os02g36140	terpene synthase, putative	1,68	1,56	1,35
LOC_Os12g30824	terpene synthase, putative			0,97
LOC_Os02g30310	ThiF family domain containing protein, putative			-0,80
LOC_Os03g17350	white-brown complex homolog protein, putative	1,42		0,97

generation of precursor metabolites and energy

LOC_Os01g55540	aminotransferase, classes I and II, domain containing protein			-0,87
LOC_Os12g10570	ATP synthase subunit beta, putative	-0,72		-0,80
LOC_Os05g47980	ATP synthase, putative		-2,24	
LOC_Os10g38276	chloroplast ATP synthase a chain precursor, putative		-1,65	-1,13
LOC_Os08g14860	cytochrome b-c1 complex subunit 7, putative			-1,22
LOC_Os02g54880	cytochrome c oxidase subunit, putative	-0,76		-1,18
LOC_Os01g66180	cytochrome c, putative			-0,83
LOC_Os01g70960	cytochrome c1-1, heme protein, mitochondrial precursor, putative		-1,35	
LOC_Os01g43710	cytochrome P450 72A1, putative		0,84	1,46
LOC_Os03g55240	cytochrome P450, putative		1,51	2,38
LOC_Os03g37290	cytochrome P450, putative	1,39	1,34	1,21
LOC_Os10g38090	cytochrome P450, putative		-0,84	
LOC_Os10g08319	cytochrome P450, putative	1,11	-0,87	
LOC_Os09g27510	cytochrome P450, putative		0,83	0,99
LOC_Os10g09110	cytochrome P450, putative			1,38
LOC_Os02g36110	cytochrome P450, putative	1,25	0,87	1,39
LOC_Os06g04510	enolase, putative	1,18	2,21	2,58
LOC_Os11g09850	expressed protein			2,84
LOC_Os05g33380	fructose-bisphospate aldolase isozyme, putative		0,70	
LOC_Os01g02880	fructose-bisphospate aldolase isozyme, putative	-0,84	-1,51	
LOC_Os01g48960	glutamate synthase, chloroplast precursor, putative		-1,33	-1,49
LOC_Os02g56850	glutathione reductase, putative		-1,28	
LOC_Os05g49880	lactate/malate dehydrogenase, putative			-0,70
LOC_Os01g61380	lactate/malate dehydrogenase, putative			-1,21
LOC_Os03g52860	lipoxygenase, putative	1,38		

LOC_Os08g44340	monodehydroascorbate reductase, putative	-0,74	
LOC_Os02g47790	monodehydroascorbate reductase, putative		0,76
LOC_Os02g53130	nitrate reductase, putative	0,88	
LOC_Os08g27840	phosphoenolpyruvate carboxylase, putative		0,98
LOC_Os09g14670	phosphoenolpyruvate carboxylase, putative		-1,38
LOC_Os01g57880	plastocyanin-like domain containing protein, putative		0,83
LOC_Os08g25720	pyrophosphate--fructose 6-phosphate 1-phosphotransferase subunit alpha, putative		-0,81
LOC_Os06g13810	pyrophosphate--fructose 6-phosphate 1-phosphotransferase subunit beta, putative		-0,95
LOC_Os01g16960	pyruvate kinase, putative		0,71
LOC_Os07g33680	succinate dehydrogenase subunit 3, putative		-4,49
LOC_Os11g06890	vacuolar ATP synthase, putative	-1,21	
