# Supplementary material for: Physiological Basis and Transcriptional Profiling of Three Salt-Tolerant Mutant Lines of Rice
Source: Front Plant Sci. 2016 Sep 28;7:1462. doi: 10.3389/fpls.2016.01462 (PMC5039197; doi:10.3389/fpls.2016.01462)
Supplement: Supplementary file 5 [file Table5.PDF]

Supplementary table S5.- Sequences of primers used in quantitative RT-PCR analysis.

**gene**	**Locus**	**primers Forward / Reverse (5' – 3')**
monovalent cation: oxidoreductase - aldo/keto reductase family protein	LOC_Os04g27060	GATGGGTGAGCTGAAGAAGC / CAATGCCAAGTTCTCTGCAA
proton antiporter-2	LOC_Os11g42790	ATTGCATTGGCCTACAACAAG / CAGGATCAGTGGATCAAGGAA
Thionin-7	LOC_Os06g32160	CAGTAGCCTTTCAGCCCTCTT / GCAACAACTCTTTGCTTCCAC
enolase	LOC_Os06g04510	CTTGCAATGTGGACCGTAACT / TGCCAAGGTAAACGACAAATC
jasmonate O-methyltransferase	LOC_Os06g21760	CGGCTTACAACAAGGGGAAG / CCGACGAAGACCAGGAACAT
Peroxidase precursor	LOC_Os07g48010	TCGACAACGCCTACTACAGCA / CTTTGGAGCAGCTAAGCCTGA
tyrosine protein kinase domain containing protein	LOC_Os03g06330	CCGTTGCTGTAAAGGATGTGT / CAGAAGGAACAAAACGCAGAG
OsRCI2-7	LOC_Os05g03130	CTGGATCAGTGGAGCTAGTCG / GTGATCATACCCACGGAAGAA
