# Supplementary material for: Physiological Basis and Transcriptional Profiling of Three Salt-Tolerant Mutant Lines of Rice
Source: Front Plant Sci. 2016 Sep 28;7:1462. doi: 10.3389/fpls.2016.01462 (PMC5039197; doi:10.3389/fpls.2016.01462)
Supplement: Supplementary file 6 [file Image1.PDF]

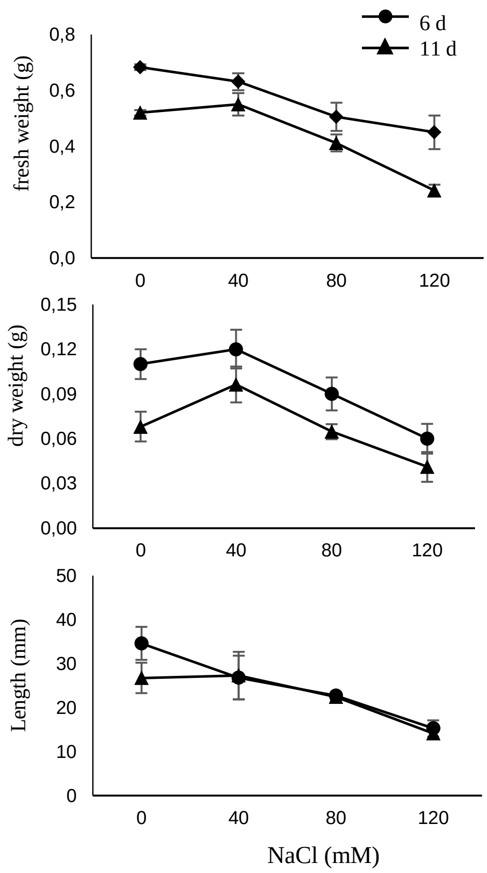

Domingo et al.

Supplementary figure S1.- Salinity effect on Bahia seedling growth. Plants were grown in hydroponic culture for 6 days and 0, 40, 80 and 120 mM NaCl were added to the culture medium. After 6 and 11 days, fresh weight, dry weight and culm length were measured. Standard deviations are shown
