# Supplementary material for: Physiological Basis and Transcriptional Profiling of Three Salt-Tolerant Mutant Lines of Rice
Source: Front Plant Sci. 2016 Sep 28;7:1462. doi: 10.3389/fpls.2016.01462 (PMC5039197; doi:10.3389/fpls.2016.01462)
Supplement: Supplementary file 7 [file Image2.PDF]

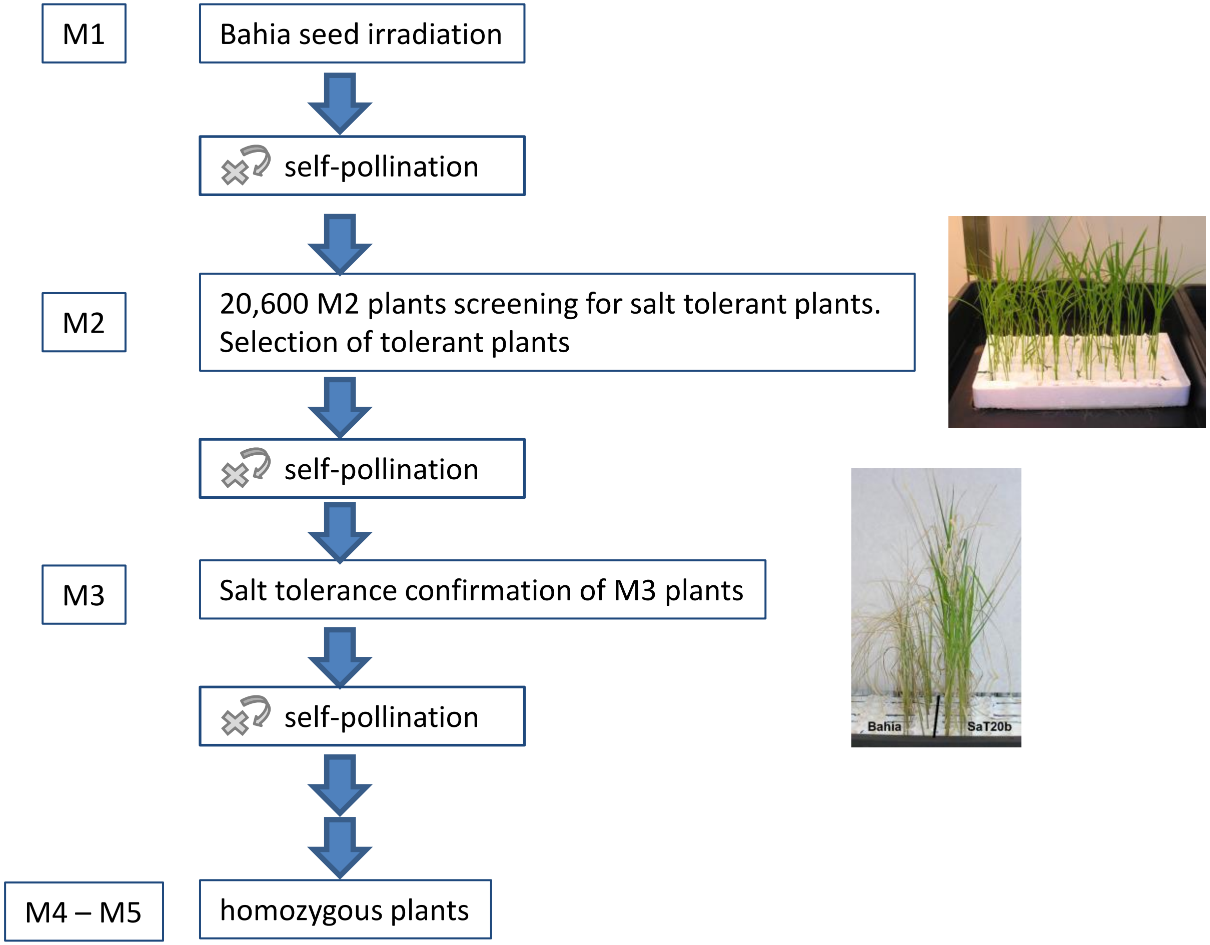

Supplementary Figure S2.- Diagram of the identification and selection process of salt tolerant plants. M2 mutant lines of rice were screened at seedling stage in hydroponic culture in the presence of 120 mM NaCl. Plants that survived after 4 weeks of culture were selected and salt tolerance phenotypes were confirmed in the subsequent generation. The highest tolerant lines were finally selected for further characterization and homozygous plants were obtained by self-pollination
