# Supplementary material for: Physiological Basis and Transcriptional Profiling of Three Salt-Tolerant Mutant Lines of Rice
Source: Front Plant Sci. 2016 Sep 28;7:1462. doi: 10.3389/fpls.2016.01462 (PMC5039197; doi:10.3389/fpls.2016.01462)
Supplement: Supplementary file 8 [file Image3.PDF]

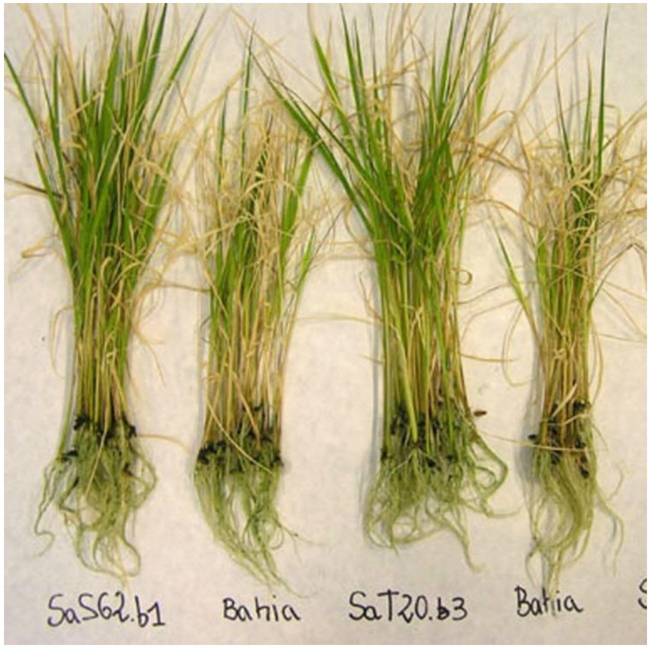

Domingo et al.
Supplemental Figure S3.- *SaS62* and *SaT20* plants (M4) grown in hydroponic culture in the presence of 120 mM NaCl during 4 weeks compared to Bahia plants. Plants were grown under the same growth conditions in the same cultures tray.
