# Supplementary material for: Physiological Basis and Transcriptional Profiling of Three Salt-Tolerant Mutant Lines of Rice
Source: Front Plant Sci. 2016 Sep 28;7:1462. doi: 10.3389/fpls.2016.01462 (PMC5039197; doi:10.3389/fpls.2016.01462)
Supplement: Supplementary file 9 [file Image4.PDF]

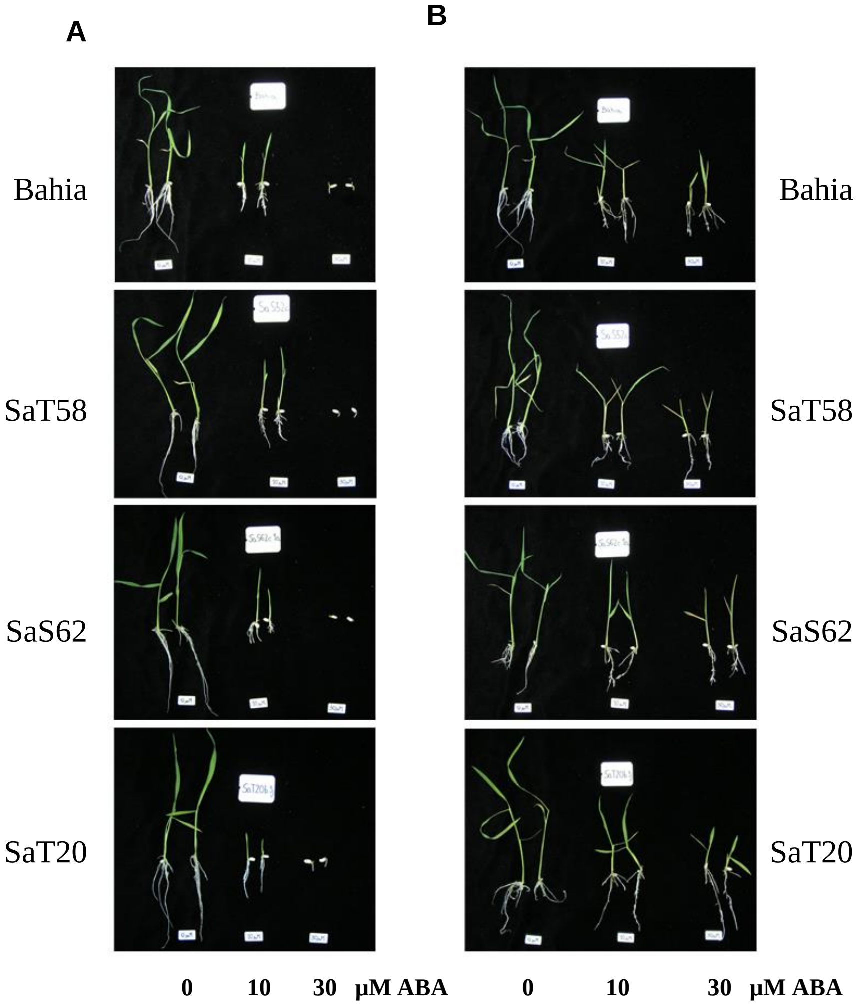

Domingo et al.

Supplementary Figure S4.- ABA sensitivity assay on SaT58, SaS62, SaT20 and Bahia seedlings. (A) Seeds were germinated and grown on MS medium and transferred to MS medium plus 0, 10 or 30 µM ABA. (B) seeds were germinated in the presence of 0, 10 or 30 µM ABA and grown under the same conditions. After 7 d of treatment, the phenotype was recorded
