# Supplementary material for: Physiological Basis and Transcriptional Profiling of Three Salt-Tolerant Mutant Lines of Rice
Source: Front Plant Sci. 2016 Sep 28;7:1462. doi: 10.3389/fpls.2016.01462 (PMC5039197; doi:10.3389/fpls.2016.01462)
Supplement: Supplementary file 10 [file Image5.PDF]

A

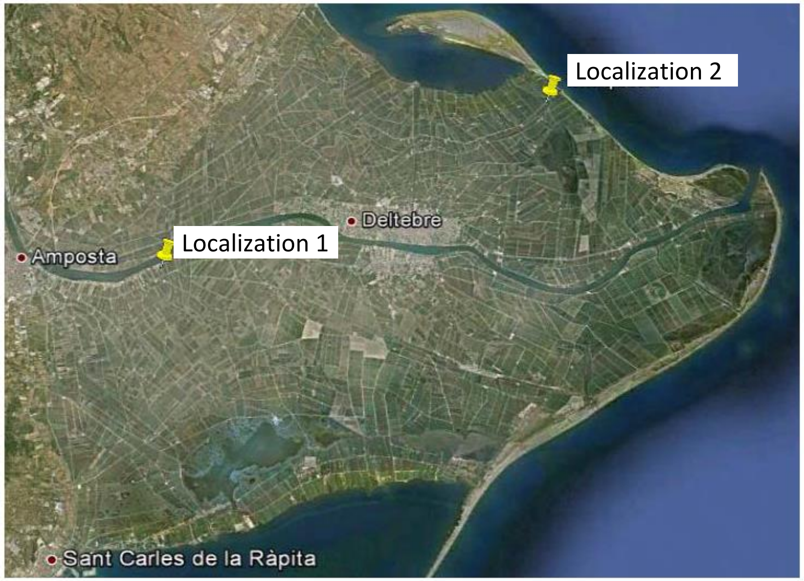

B

	Localization 1 (non-affected land)	Localization 2 (salt-affected land)
**C.E. (saturated extract)**	4,79 dS/m	41,10 dS/m
**S.A.R.**	5,11	47,20
**E.S.P.**	5,86	39,63
**pH**	7,90	8,15
**texture**	Siltyclay loam	Silty loam

Domingo et al.

Supplementary Figure S5.- (a) Localization of field where agronomical evaluations were performed (Google Earth, Deltebre 40˚43’10’’N 0˚42’30’’E). Non-salt conditions were due at IRTA and salt affected land was located at Marquesa fields, next to the sea line (indicated by yellow markers). (b) Soil characteristics in both locations. Mean values from 2011 and 2012 are shown. C.E: electric conductivity of soil saturated extract, S.A.R.: Sodium Adsorption Rate; E.S.P.: Exchangeable Sodium Percentage
